# Radical-like reactivity for dihydrogen activation by coinage metal–aluminyl complexes: computational evidence inspired by experimental main group chemistry†

**DOI:** 10.1039/d2sc05815d

**Published:** 2022-12-15

**Authors:** Diego Sorbelli, Leonardo Belpassi, Paola Belanzoni

**Affiliations:** a Department of Chemistry, Biology and Biotechnology, University of Perugia Via Elce di Sotto 8 – 06123 Perugia Italy diegosorbelli00@gmail.com paola.belanzoni@unipg.it; b CNR Institute of Chemical Science and Technologies “Giulio Natta” (CNR-SCITEC) Via Elce di Sotto 8 – 06123 Perugia Italy leonardo.belpassi@cnr.it

## Abstract

The computational study of an unprecedented reactivity of coinage metal–aluminyl complexes with dihydrogen is reported. In close resemblance to group 14 dimetallenes and dimetallynes, the complexes are predicted to activate H_2_ under mild conditions. Two different reaction pathways are found disclosing a common driving force, *i.e.*, the nucleophilic behavior of the electron-sharing M–Al (M = Cu, Ag, Au) bond, which enables a cooperative and diradical-like mechanism. This mode of chemical reactivity emerges as a new paradigm for dihydrogen activation and calls for experimental feedback.

## Abstract 2

Digermynes activate H_2_ under mild experimental conditions. Coinage metal–aluminyl complexes, featuring electron-sharing M–Al bonds similar to Ge–Ge, are predicted to activate H_2_, with potential application in hydrogenation catalysis.

## Graphical abstract

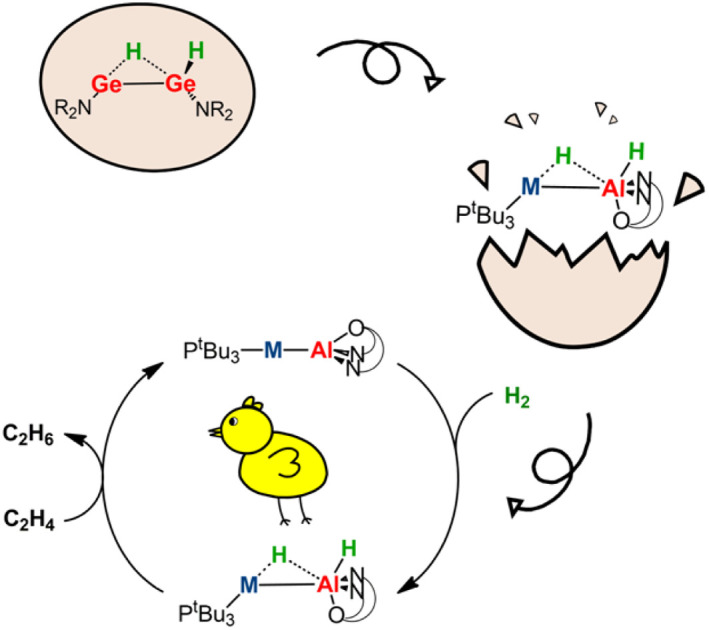

## Introduction

**Scheme 1:**
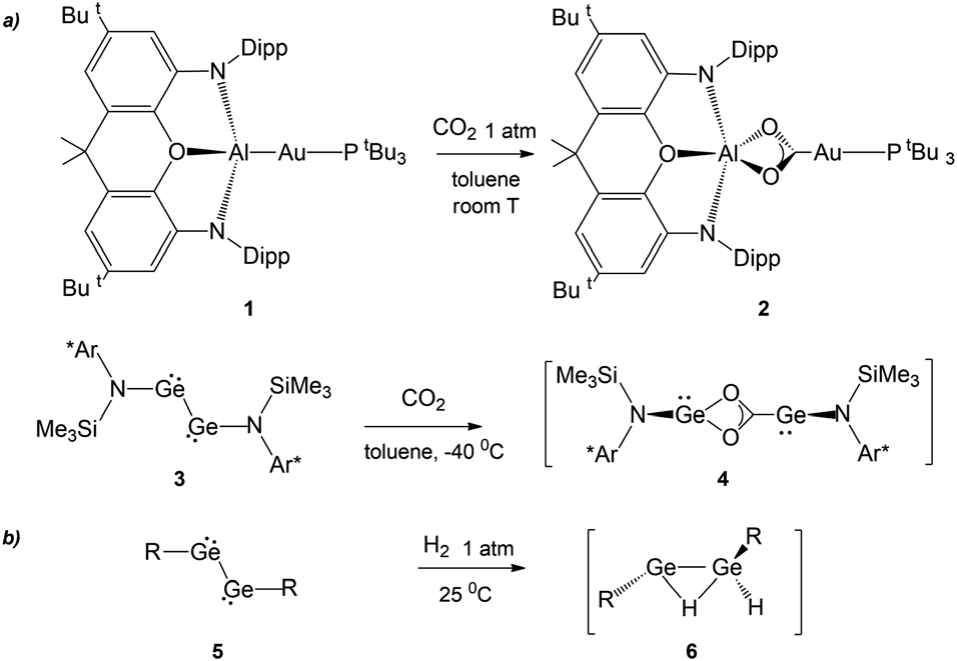
(a) Similar reactivity of [^*t*^Bu_3_PAuAl(NON)] (NON = 4,5-bis(2,6-diisopropylanilido)-2,7-di-*tert*-butyl-9,9-dimethylxanthene) complex 1 and (Ar*)(SiMe_3_)GeGe(Ar*)(SiMe_3_) (Ar* = C_6_H_2_{C(H)PH_2_}_2_Me-2,6,4) complex 3 with CO_2_.^7^ (b) First step in the mechanism of the reaction of several aryl- and amido-digermyne compounds 5 with H_2_. (R = [C_6_H_3_-2,6(C_6_H_3_-2,6^i^Pr_2_)_2_],^8^ [N(SiMe_3_)(C_6_H_2_Me{C(H)Ph_2_}_2_)],^9^ and [N(Si^i^Pr_3_)(2,6-[C(H)Ph_2_]2-4-^i^PrC_6_H_2_)]^10^).

The recent synthesis of the gold–aluminyl complex 1 (Scheme 1) and its outstanding reactivity in capturing CO_2_ under mild experimental conditions represent a unique case in the framework of gold chemistry.^1^ We have thoroughly discussed that the driving force of this reactivity is the presence of an electron-sharing, weakly polarized Au–Al bond acting as a nucleophilic site inducing a cooperative diradical-like reactivity.^2–5^ Shortly after this, analogous reactivity was reported to occur for copper– and silver–aluminyl complexes, for which, however, the reaction proceeds towards the formation of carbonate complexes with CO extrusion.^6^

A surprising feature concerning the reaction of 1 with carbon dioxide is that it bears strict analogies with amido-digermyne compound 3 (Scheme 1), which, in reducing CO_2_ to CO, proceeds through an intermediate 4 that is structurally very similar to insertion product 2.^7^ Particularly intriguing, aryl- and amido-digermynes RGeGeR (R = [C_6_H_3_-2,6(C_6_H_3_-2,6^i^Pr_2_)_2_],^8^ [N(SiMe_3_)(C_6_H_2_Me{C(H)Ph_2_}_2_)],^9^ and [N(Si^i^Pr_3_)(2,6-[C(H)Ph_2_]2-4-^i^PrC_6_H_2_)]^10^) 5 (Scheme 1) are reported to easily activate H_2_ under mild experimental conditions both in solution^8,10^ and the solid state at temperatures as low as −10 °C.^9^ In all cases, experiments and theoretical investigations outline a reaction mechanism that proceeds *via* a singly bridged intermediate [RGe(μ-H)GeHR] species 6, which subsequently, upon isomerization, yields different hydrogenation products, which are experimentally revealed depending on the steric hindrance of the substituents.^11,12^ Notably, the facile reactivity with H_2_ has also been reported for other group 14 dimetallenes and dimetallynes.^13^ The digermyne is suggested to possess substantial diradical character and therefore to react through H atom abstraction from H_2_, followed by recombination of the resultant radical pair.^8^

**Scheme 2:**
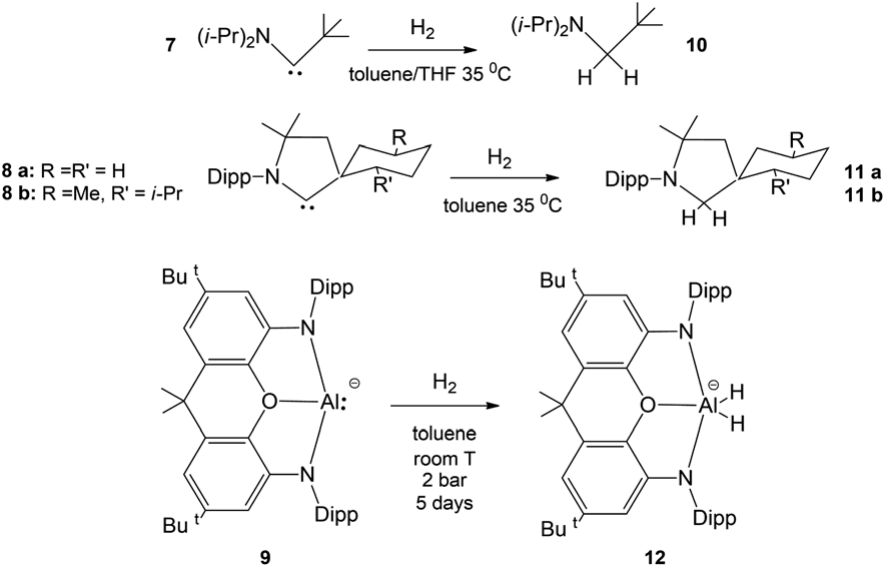
Similar reactivity of (alkyl)(amino)carbenes 7 and 8a, b and aluminyl 9 with H_2_.

As bare ligands, aluminyls resemble singlet carbenes in possessing an electron lone pair and an accessible vacant p orbital, thus potentially showing a similar reactivity. Interestingly, it has been reported that acyclic and cyclic (alkyl)(amino) carbenes 7 and 8a, b can activate H_2_ under mild conditions, by behaving as nucleophiles (Scheme 2).^14^ In analogy, the ability of [M{Al(NON)}]_2_ (M = Li, Na, K) species to react with H_2_ has been also reported (Scheme 2, complex 9)^15–17^ together with detailed mechanistic studies.^18^ Despite these similarities, when used as coordination ligands, aluminyls and carbenes have a strikingly different chemical behaviour. In a recent work, we have shown that gold–aluminyl complexes, unlike gold carbene analogues featuring a dative Au–C bond, are able to react with CO_2_.^4^

In this scenario the interest in exploring the reactivity of gold–aluminyl complexes towards H_2_ naturally arises. The electron-sharing nature of the Au–Al bond in complex 1 allowed the rationalization of its reactivity with CO_2_,^2^ in close resemblance with that of digermyne complex 3, and thus, we reasoned that the same bonding model may also favor the reaction of complex 1 with H_2_ analogous to that of complexes 5. The possibility for H_2_ activation to occur with aluminyl complexes is very attractive, especially in light of potential applications in hydrogen storage and catalysis, beside H_2_ being vital in several industrial processes, organic synthesis and also in biological functions.^19,20^

In addition, given the similar reactivity of gold- copper- and silver–aluminyl complexes with CO_2_, a possible H_2_ activation by copper– and silver–aluminyl complexes is certainly worth exploring.

Herein we report that [^*t*^Bu_3_PMAl(NON)] (M = Cu, Ag, Au) complexes should indeed react with dihydrogen, with the Cu–Al complex featuring the most kinetically favored and exergonic reaction with H_2_. The calculations predict the experimentally accessible formation of a singly bridged [^*t*^Bu_3_PM(μ-H)Al(H)(NON)] species for all the metal–aluminyl complexes and an additional doubly bridged [^*t*^Bu_3_PM(μ-H)_2_Al(NON)] product for copper and silver. Detailed electronic structure calculations highlight the central role of the electron-sharing M–Al bond in inducing a diradical-like reactivity towards H_2_, in close resemblance to digermynes, as surmised.

## Results and discussion

**Fig. 1:**
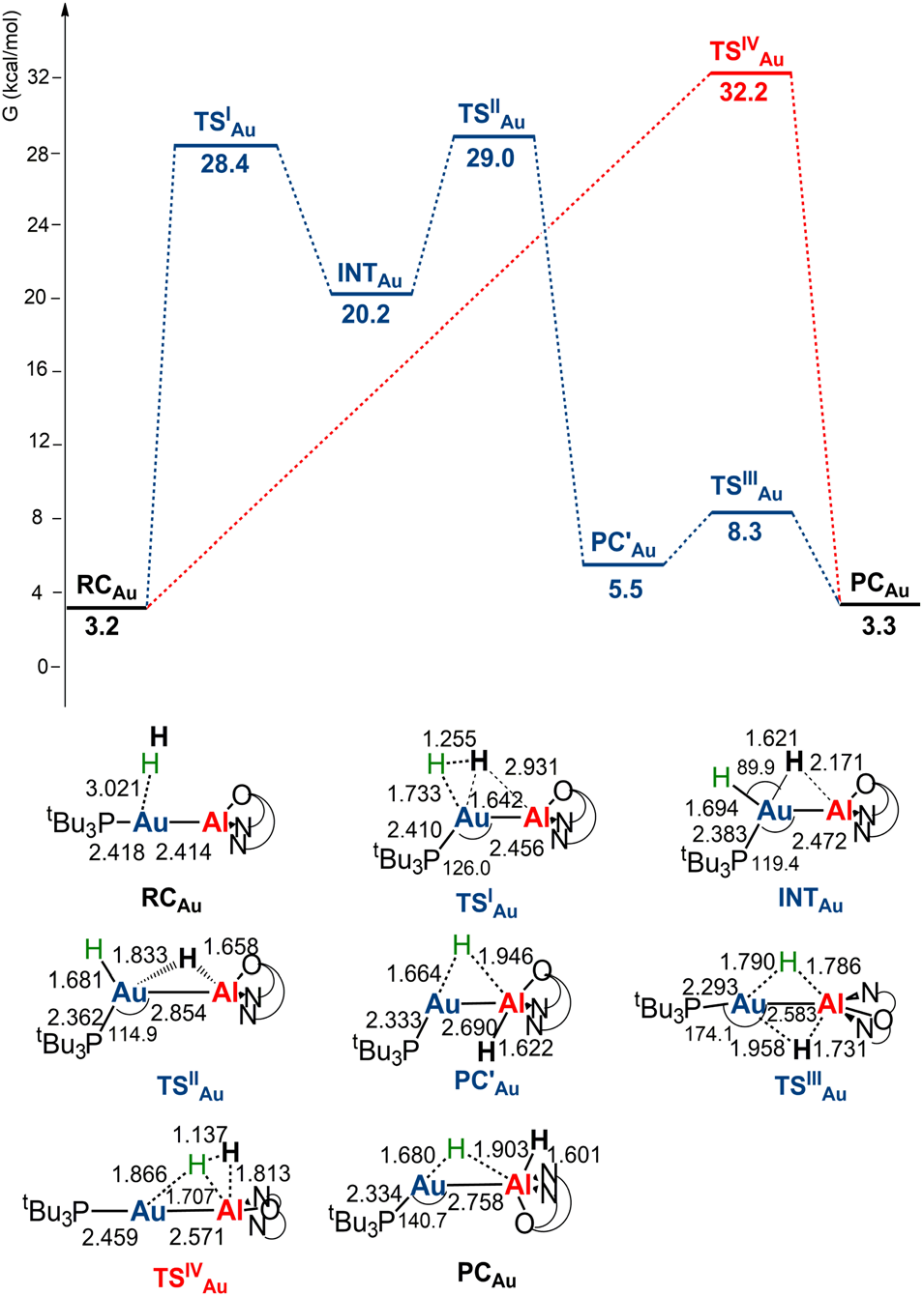
Free energy profile for the reaction of [^*t*^Bu_3_PAuAl(NON)] with H_2_ at the Au (blue line) and the Al (red line) sites. Δ*G* values refer to the energy of the separated reactants taken as zero. Selected interatomic distances (Å) and bond angles (degrees) are shown with all the stationary point structures.

The free energy profiles for the reaction of [^*t*^Bu_3_PMAl(NON)] complexes with H_2_ have been calculated using the same computational protocol employed in ref. 2 for the reaction of [^*t*^Bu_3_PAuAl(NON)] with CO_2_ (see Computational details). For [^*t*^Bu_3_PAuAl(NON)], the free energy profile is depicted in Fig. 1 together with the schematic structure of all stationary points. The optimized structures of all the stationary points in Fig. 1 are reported in the ESI (Fig. S1).†

Starting from the reactant complex (RC_Au_), H_2_ activation can occur both at the Au (blue line profile, Δ*G* = 28.4 kcal mol^−1^) and the Al (red line profile, Δ*G* = 32.2 kcal mol^−1^) sites, with activation at Au being favoured. From the Al site, the reaction leads directly to the formation of the *cis*-[^*t*^Bu_3_PAu(μ-H)Al(H)(NON)] singly bridged species PC_Au_*via* the concerted transition state TS^IV^_Au_. From the Au site, a first step (formally a H_2_ “oxidative addition” to Au *via* TS^I^_Au_) yields an unstable intermediate species INT_Au_ which rearranges (*via* TS^II^_Au_, Δ*G* = 29.0 kcal mol^−1^) by a hydrogen transfer to Al, leading to the singly bridged *trans*-[^*t*^Bu_3_PAu(μ-H)Al(H)(NON)] species 
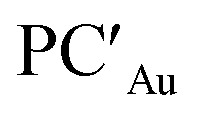
 which is in equilibrium with the more stable PC_Au_ (*via* TS^III^_Au_, Δ*G* = 8.3 kcal mol^−1^). Interestingly, the overall RC_Au_-to-PC_Au_ conversion is practically thermoneutral, suggesting a possibly reversible reaction and a virtually ideal condition for the use of complex 1 as a hydrogen-transfer catalyst. The free energy activation barriers for the two paths roughly fit in the 25–30 kcal mol^−1^ range, typically observed for experimental H_2_ activation processes under mild conditions (see ref. 18 and 21–24 for some recent examples).

The structural analogy between the gold–aluminyl PC_Au_ and digermyne 6 (Scheme 1) singly bridged species is striking.

Notably, at variance with digermyne 6, PC_Au_ is not expected to easily undergo isomerization (see Scheme S1 and Discussion in the ESI†) and, in particular, dissociation into two separate hydride complexes (*i.e.* [^*t*^Bu_3_PAuH] and [HAl(NON)]) (PC_Au__HH, see Fig. S1†).

**Fig. 2:**
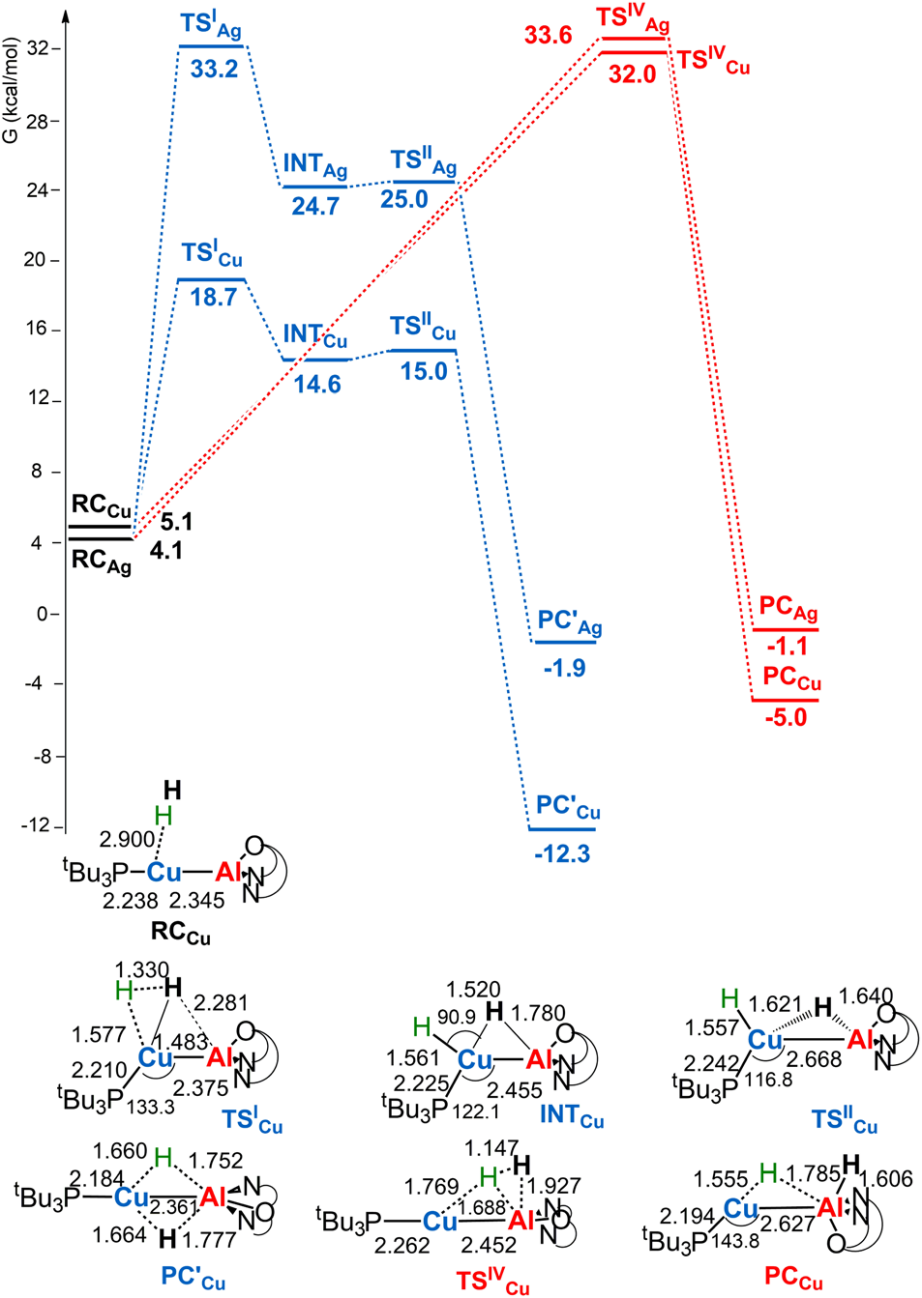
Free energy profile for the reaction of [^*t*^Bu_3_PMAl(NON)] (M = Cu, Ag) with H_2_ at the M (blue line) and the Al (red line) sites. Δ*G* values refer to the energy of the separated reactants taken as zero. Selected interatomic distances (Å) and bond angles (degrees) are shown with all the stationary point structures for the [^*t*^Bu_3_PCuAl(NON)] complex (for the [^*t*^Bu_3_PAgAl(NON)] complex see Fig. S3 in the ESI†).

Given the thermoneutrality of this reaction, experimental evidence for H_2_ cleavage could not be straightforward. In light of the reported more efficient reaction of copper– and silver–aluminyl complexes with carbon dioxide, it is interesting at this point to investigate the reactivity of [^*t*^Bu_3_PMAl(NON)] (M = Cu, Ag) complexes with H_2._ The reaction profiles are depicted in Fig. 2. The structures of all stationary points for both copper– and silver–aluminyl complexes are shown in Fig. S2–S4 in the ESI.†

For the silver– and copper–aluminyls, H_2_ activation can occur symmetrically at the Ag (blue line profile, Δ*G* = 33.2 kcal mol^−1^) and the Al (red line profile, Δ*G* = 33.6 kcal mol^−1^) sites, and remarkably unsymmetrically at the Cu (blue line profile, Δ*G* = 18.7 kcal mol^−1^) and the Al (red line profile, Δ*G* = 32.0 kcal mol^−1^) sites. From the Al site, the reaction leads directly to the formation of the singly bridged aluminyl species PC_Cu_/PC_Ag_ for both copper and silver complexes *via* the concerted transition state TS^IV^_Cu_/TS^IV^_Ag_. Although both are found to have similar energy to that of TS^IV^_Au_, the respective products are found to be more stable. In particular, the formation of PC_Ag_ is slightly exergonic (−1.1 kcal mol^−1^) and the formation of PC_Cu_ is even more exergonic (−5.0 kcal mol^−1^).

The alternative path, where H_2_ approaches the complexes closer to the metal site (blue paths in Fig. 2), highlights appreciable differences between lighter coinage metals and gold, instead. Qualitatively, the first step is analogous in all cases: H_2_ approaches the metal site, yielding intermediates INT_Cu_/INT_Ag_. In the second step, however, copper and silver complexes remarkably differ from gold, since the intermediate is converted unequivocally to a stable doubly bridged product [^*t*^Bu_3_PM(μ-H)_2_Al(NON)] (M = Cu, Ag) in both cases 
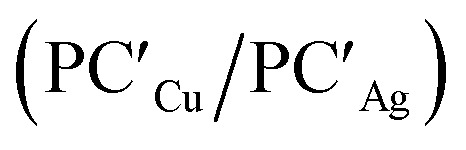
.

From a quantitative perspective, differences between the three metals are even more pronounced. Indeed, the silver–aluminyl complex displays a higher activation barrier for TS^I^_Ag_ (33.2 kcal mol^−1^) and a slightly exergonic formation of 
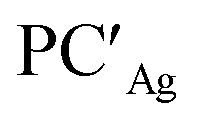
 (Δ*G* = −1.9 kcal mol^−1^) *via* isomerization from the unstable INT_Ag_. On the other hand, the path for the copper–aluminyl complex shows much more favorable energetics overall with respect to silver and gold complexes. In the first step, a more stable intermediate (INT_Cu_) is formed *via* a much lower activation barrier at TSI_Cu_ (Δ*G* = 18.7 kcal mol^−1^). Subsequently, the INT_Cu_ isomerization leads to the highly stable doubly bridged product 
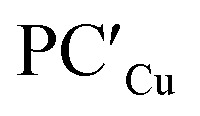
 (Δ*G* = −12.3 kcal mol^−1^). Remarkably, 
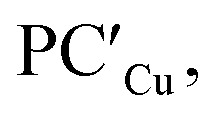

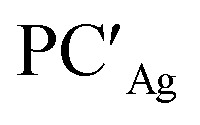
 and TS^III^_Au_ structures are similar, even though for gold the doubly bridged species is not a minimum energy point. Thus, preferential formation of singly bridged aluminyl species with the second hydrogen bound to Al is found for gold.

The reaction profiles described above clearly indicate that coinage metal–aluminyl complexes can activate H_2_, and a particularly facile H_2_ cleavage is predicted with the copper–aluminyl complex. Based on the 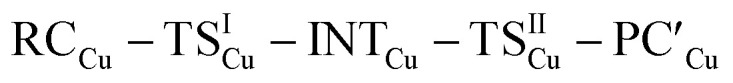 path, due to the relatively low activation barrier and high exergonicity, dihydrogen activation could be experimentally demonstrated more easily. Under suitable conditions, isolation and characterization of the doubly bridged copper species should be achievable. Furthermore, as reported extensively in the literature,^25–30^ this type of reaction may be affected by the presence of tunnel effects that contribute to the lowering of the activation barriers. With the aim of assessing if such effects may be important in this context, we used transition state theory (TST)^31^ and the Eckart tunneling correction^32^ (see Computational details) to estimate the tunnel effect on the Eyring fitted activation barrier associated with TS^I^_M_. The results show that, indeed, a moderate tunnel-related lowering of the activation barriers associated with TS^I^_M_ may be expected (in the range of 1.3–3.7 kcal mol^−1^, see Table S1†). A proper assessment of this effect is beyond the scope of this work, but this estimate suggests that the tunnel effects may impact, to some extent, the reactivity reported here.

A common feature of the reaction profiles in Fig. 1 and 2 is the possible H_2_ activation at two different sites (M and Al), apparently suggesting that the two distinct paths are initiated by interactions of a different nature when H_2_ approaches the complexes (transition states TS^I^_M_ and TS^IV^_M_). To investigate the nature of the interaction between H_2_ and [^*t*^Bu_3_PMAl(NON)] (M = Cu, Ag, Au) at the corresponding TS^I^_M_ and TS^IV^_M_, natural orbitals for chemical valence (NOCV) analysis,^33,34^ energy decomposition analysis (EDA)^35–37^ and charge displacement (CD)^38–40^ approaches have been applied (see Table S2 and Fig. S5–S16 in the ESI†).

**Fig. 3:**
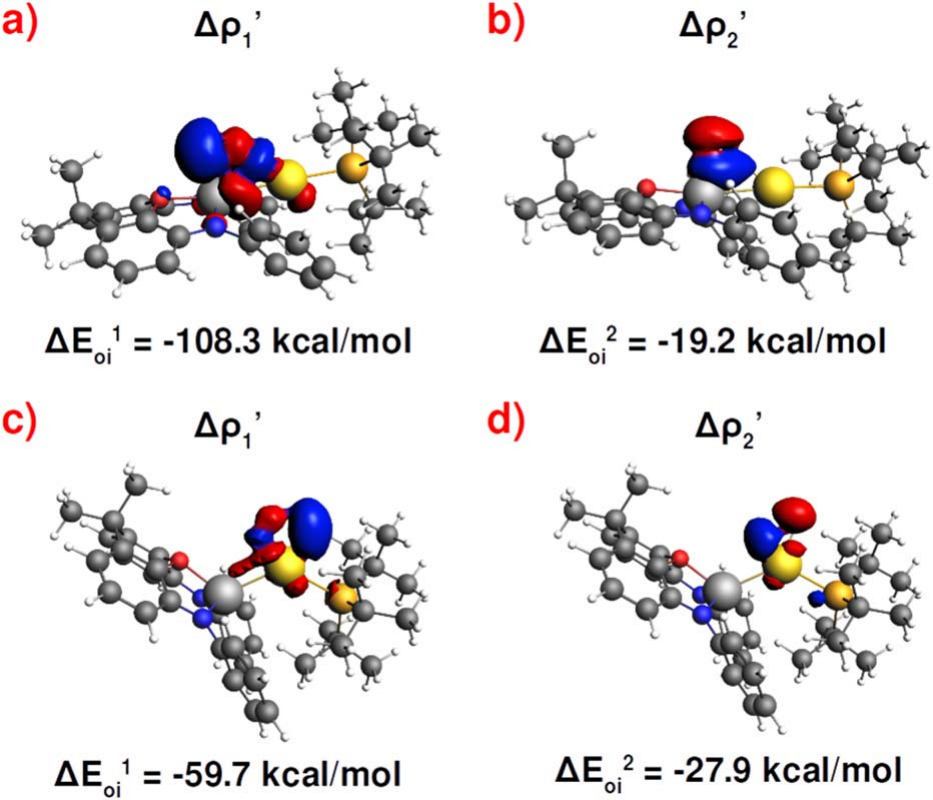
Results of the NOCV analysis of the [H_2_]–[^*t*^Bu_3_PAuAl(NON)] interaction at TS^IV^ (top) and TS^I^ (bottom). The isosurfaces for the 
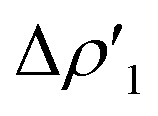
 and 
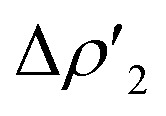
 components for TS^IV^ ((a) and (b), respectively) and TS^I^ ((c) and (d), respectively) are reported together with the associated orbital interaction energy. The charge flux is red → blue. The isovalue is 3*me*/*a*_0_^3^ for all isosurfaces. See Fig. S5–S8 in the ESI† for the complete NOCV analysis.

As shown in Fig. 3, the NOCV analysis at TS^IV^_Au_ for the gold–aluminyl complex reveals a main component 
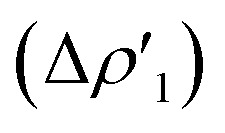
 of the H_2_–[^*t*^Bu_3_PAuAl(NON)] interaction, which can be depicted, upon decomposition of the corresponding donor and acceptor NOCVs (see Fig. S5 and S6 in the ESI†), as mainly dominated by a charge transfer from the HOMO of the complex (a σ MO representing the electron-sharing Au–Al bond) towards the LUMO of H_2_ (Fig. 2a and S5†). A second non-negligible component can be envisaged (
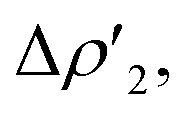
Fig. 2b and S6†), representing a charge transfer from the H_2_ HOMO to the [^*t*^Bu_3_PAuAl(NON)] LUMO, mainly composed of the empty 3p_*z*_ orbital of Al. Notably, this NOCV description of the H_2_–[^*t*^Bu_3_PAuAl(NON)] interaction is strongly reminiscent of both the CO_2_–[^*t*^Bu_3_PAuAl(NON)] interaction (see ref. 2–5) and the H_2_–[RGeGeR] interaction reported by Frenking and coworkers, where the digermyne, by employing the σ Ge–Ge HOMO and the 4p_*z*_-centred LUMO, interacts with the H_2_ LUMO and HOMO, respectively.^12^

Surprisingly, despite remarkable structural and energetic differences, the H_2_–[^*t*^Bu_3_PAuAl(NON)] interactions at TS^I^_Au_ are qualitatively similar to those at TS^IV^_Au_. As shown in Fig. 2c and d (Fig. S7 and S8 in the ESI†), the NOCV analysis analogously reveals two dominant components: 
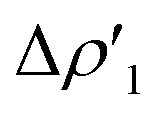
 consisting of a charge transfer from the σ Au–Al HOMO of the complex towards the LUMO of H_2_, and 
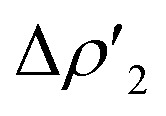
 featuring a charge transfer from the H_2_ HOMO towards the LUMO of the complex.

Notably, analogous results arise upon NOCV analysis of the H_2_-complex interaction at TS^IV^_M_ and TS^I^_M_ for copper– and silver–aluminyl complexes. As can be inferred from the results reported in Fig. S9–S16 in the ESI,† at both TS^I^_Cu_/TS^I^_Ag_ and TS^IV^_Cu_/TS^IV^_Ag_ the main component of the H_2_-complex interaction is represented by a charge transfer from the σ bonding M–Al molecular orbital towards the H_2_ antibonding LUMO. This finding is fully consistent with the nature of the M–Al bond. As previously reported for the Cu–Al bond^2^ and based on Fig. S17, S18 and Table S3 in the ESI and Discussion therein,† despite small changes in the bond polarization, in these complexes the M–Al (M = Cu, Al, Au) is an electron-sharing bond, acting as a nucleophilic site for the activation of H_2_.

Furthermore, similar to the gold complex, both copper– and silver–aluminyl complexes feature a second driving force in the interaction with H_2_ at the two TSs, that is a charge transfer from the H_2_ HOMO towards the LUMO of the complex. So apparently, for all the metals and at both TSs, the nature of the H_2_-complex interaction is qualitatively analogous.

Differences between TS^IV^_M_ and TS^I^_M_ and between copper and its heavier homologues silver and gold become evident on a quantitative ground. In particular, the ETS-NOCV and CD analyses reveal that 
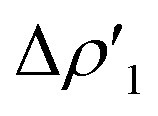
 and 
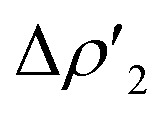
 have different relative weights in the H_2_–[^*t*^Bu_3_PMAl(NON)] interaction. Indeed, for the gold complex, the associated Δ*E*_oi_^1^/Δ*E*_oi_^2^ and CT_1_/CT_2_ values are respectively −108.3/−19.2 kcal mol^−1^ and 0.18/0.07 e at TS^IV^_Au_, while these values are −59.7/−27.9 kcal mol^−1^ and 0.18/0.12 for TS^I^_Au_ (Table S2 in the ESI†). An analogous trend is also observed for the Δ*E*_oi_^1^/Δ*E*_oi_^2^ (CT_1_/CT_2_) values associated with TS^IV^_M_ and TS^I^_M_ (M = Cu, Ag), where the weight of the H_2_-to-complex charge transfer increases at TS^I^_M_ (Table S2 in the ESI†).

This result clearly shows that (i) the H_2_-complex main interaction is quantitatively similar upon changing the coinage metal and (ii) the relative weight of 
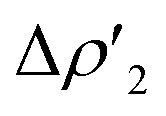
 at TS^I^_M_ is larger for all the complexes, which can be explained based on the frontier molecular orbitals (FMO) analyses. From the M and Al atomic orbital (AO) contributions to the LUMO of the [^*t*^Bu_3_PMAl(NON)] complex at the corresponding TS^IV^_M_ and TS^I^_M_ structures (Table S4 in the ESI†), differences become evident. The LUMO at TS^IV^_M_ displays a smaller coinage metal contribution compared to Al (see for instance 17.7% Cu *vs.* 23.5% Al at TS^IV^_Cu_). On the other hand, at TS^I^_M_ the metal contribution is significantly increased (see for instance 43.9% Cu at TS^I^_Cu_), with contributions from Al remaining almost unchanged. These increased metal contributions to the LUMO complex at TS^I^_M_ are responsible for a larger 
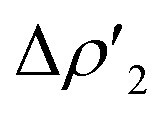
 (*i.e.* an enhanced electrophilicity of the complexes), which should arise from the highly bent P–M–Al angle at all the TS^I^_M_, favouring the LUMO delocalization over the metal and also explaining the preferred H_2_ interaction at the metal site (and the corresponding lower activation barrier).

The extent of the P–M–Al bending also rationalizes the more energetically favourable pathway for the copper complex with respect to its silver and gold analogues. While the P–M–Al angle is below 130° for both TS^I^_Ag_ and TS^I^_Au_ (129.1° and 126.0°, respectively), the Cu–Al complex features a larger P–Cu–Al angle at TS^I^_Cu_ (133.0°). This apparently marginal difference has a significant effect on the activation barrier, as shown by the activation strain model analysis^41–43^ (ASM, see Tables S5–S7 in the ESI and Discussion therein†) of the reaction pathways. The less bent copper structure actually reduces the distortion penalty associated with the complex rearrangement from 18.7 kcal mol^−1^ at TS^I^_Au_ to 5.2 kcal mol^−1^ at TS^I^_Cu_, thus lowering the activation barrier. The analysis of the variation of the metal AO contributions to the LUMO of the complex at different P–M–Al angles (see Fig. S19 and Table S8 in the ESI†) shows that the bending required to efficiently enhance the metal contribution to the LUMO is significantly lower for copper with respect to that of silver and gold, thus rationalizing the reduced bending deviation and therefore the lower distortion penalty for the copper–aluminyl system.

The coinage metal–aluminyl complex reactivity described here is very different from that of general transition metal–Lewis acid (TM–LA) compounds. For TM–LA, the presence of dative polarized TM(δ−)–LA(δ+) bonds usually favours the polarization of H_2_ and, thus, its dissociation and oxidative addition to the metal center.^44^ A quantitative assessment of the different reactivities of M–Al (M = Cu, Ag, Au) *vs.* TM–LA is given in the ESI (see Fig. S20, Table S9 and Discussion therein),† where a platinum–aluminium complex featuring a dative Pt→Al bond, reported by Bourissou and coworkers^21^ to experimentally activate H_2_, has been selected by us as a test case.

**Fig. 4:**
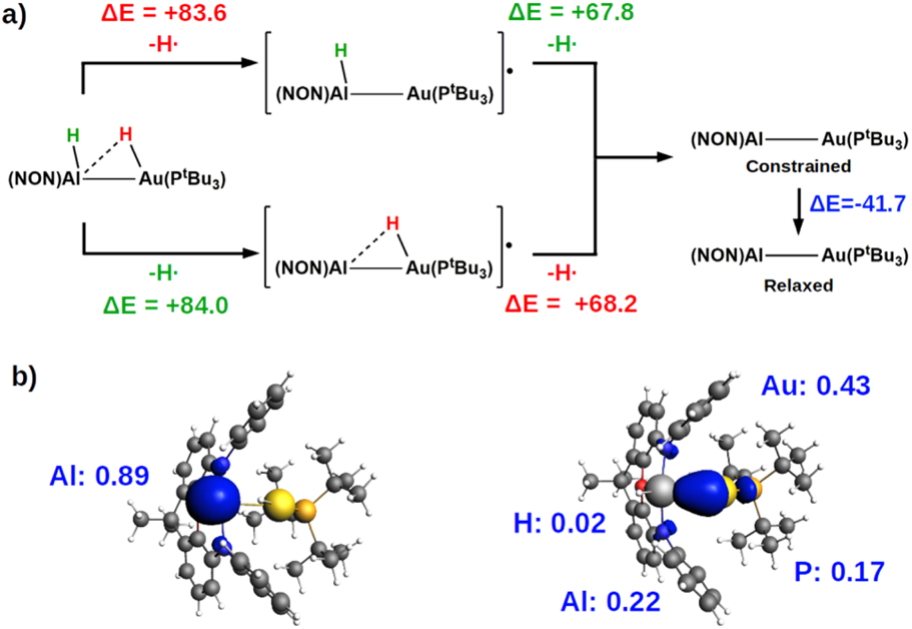
(a) Pathways for the homolytic dissociation of the two H-substrate bonds in the singly bridged product [^*t*^Bu_3_PAu(μ-H)AlH(NON)] (PC_Au_) with relative associated energies (kcal mol^−1^). (b) Spin density (in blue) associated with the [^*t*^Bu_3_PAu(μ-H)Al(NON)]˙ (left) and [^*t*^Bu_3_PAuAlH(NON)]˙ (right) radicals. The isovalue for the surface is 5*me*/*a*_0_^3^. The most relevant atomic spin polarization values are reported.

On the other hand, further analogies between gold–aluminyl (and copper– and silver–aluminyl) and digermyne compounds can be inferred by investigating the radical pair recombination mechanism for the formation of product PC_Au_ in comparison with that of 6. As mentioned earlier, this mechanism (radical H˙ abstraction followed by radical pair recombination) has been proposed for digermynes.^8^ As shown in Fig. S21 in the ESI† and in Fig. 4, we modelled homolytic and heterolytic breaking of the two substrate – H bonds in PC_Au_ for probing which of these two mechanisms is the most favourable in this case.

The most suitable fragmentation (*i.e.* the one featuring the lowest associated energy for the dissociation of the substrate – H bonds) is the radical one (see Fig. 4 and S21 in the ESI†), meaning that the most favorable mechanism would be H˙ abstraction followed by a radical pair recombination (the associated Δ*E* values are 83.6 and 84.6 kcal mol^−1^, while Δ*E* values of 362.4/179.9 kcal mol^−1^ and 195.2/388.8 kcal mol^−1^ are calculated for hydride/proton abstraction and recombination). Interestingly, the dissociation of the remaining hydrogen from the radicals to yield the gold–aluminyl complex still features positive dissociation energies (Fig. 4a), suggesting that the complex is able to stabilize the radical pair. Such an ability is preserved even when accounting for the geometrical relaxation of the gold–aluminyl complex, which is, in absolute value, smaller than the dissociation energies.

Even more interestingly, this model indirectly reveals the diradical-like nature of this mechanism. By inspection of the spin densities on the radical [^*t*^Bu_3_PAu(μ-H)Al(NON)]˙ and [^*t*^Bu_3_PAuAlH(NON)]˙ fragments (Fig. 4b), no spin polarization is observed on the bound hydrogen, with the spin density being mostly localized either on Al (spin polarization of 0.89 e) or in the region between Au and Al atoms, (spin polarization values of 0.43 and 0.22 e for Au and Al, respectively). This finding demonstrates that the radical-like reactivity of these fragments has a fundamental role in favoring the activation of H_2_*via* homolytic H–H dissociation and the consequent formation of the thermodynamically accessible PC_Au_. This picture is strongly supported by the analogous dissociation paths and spin densities (Fig. S22 in the ESI†) observed for the model digermyne reported in ref. 12.

Analogously, the same model applied for studying the formation of PC_Cu_/PC_Ag_ and 
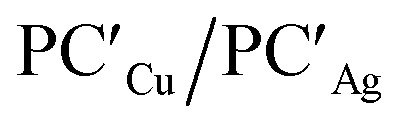
 supports a similar radical-pair stabilization mechanism for both copper– and silver–aluminyl complexes. As displayed in Fig. S23–S26 in the ESI,† H˙ abstraction followed by radical pair recombination is favored for both PC_Cu_ and PC_Ag_ (the associated Δ*E* values are 88.7/85.9 and 83.3/85.0 kcal mol^−1^ respectively, see Fig. S24†), while the Δ*E* values associated with the hydride/proton abstraction and recombination mechanism are much higher (see Fig. S23†). Analogous values have also been calculated for the same mechanisms in the case of 
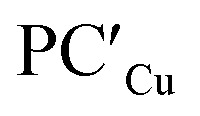
 and 
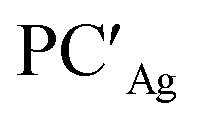
 (see Fig. S25 and S26†).

Notably, monitoring the charge migration along the reaction paths with the evolution of Voronoi deformation density (VDD)^45^ atomic charges on the metal (*q*_M_) and aluminium (*q*_Al_) atoms, as well as on the two hydrogen atoms of H_2_ (*q*_H_1__ and *q*_H_2__), indirectly confirms this mechanistic picture. As shown in Table S10 in the ESI,† both *q*_M_ and *q*_Al_ become increasingly positive along the reaction paths (indicating charge depletion), while *q*_H_1__ and *q*_H_2__ become increasingly negative (indicating charge accumulation on H_2_). Remarkably, both increases of *q*_M_/*q*_Al_ and *q*_H_1__/*q*_H_2__ are almost symmetrical for all the stationary points, indicating the absence of H_2_ polarization and further confirming the bimetallic cooperative radical pair stabilization mechanism. Furthermore, the VDD atomic charge evolution along the reaction profiles suggests that, starting from what we expect to be a formal M(0) species in the initial complexes based on previous results on [^*t*^Bu_3_PAuAl(NON)],^46^ the oxidation state at M (and Al) remains formally unaltered.

**Scheme 3:**
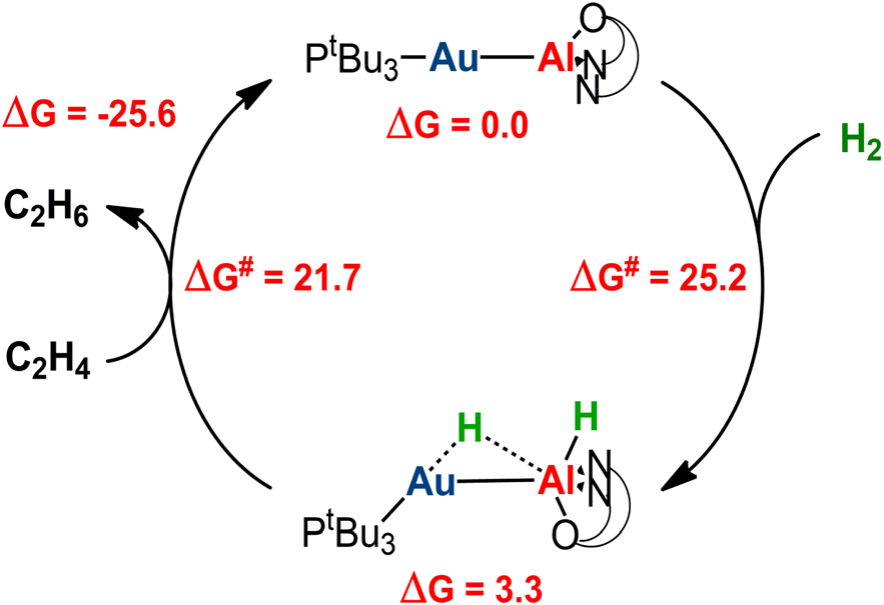
Schematic computational catalytic cycle for the hydrogenation of ethylene catalyzed by the [^*t*^Bu_3_PAuAl(NON)] complex 1. Relative free energies and free energy activation barriers are given in kcal mol^−1^.

Catalytic application of the hydrogenated 
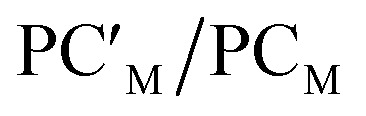
 species is an exciting prospect. In particular, the almost thermoneutral formation of PC_Au_ is virtually an ideal condition for the [^*t*^Bu_3_PAuAl(NON)] complex to be used as a catalyst in hydrogenation reactions.^47^ Selecting ethylene as a substrate, we modeled the alkene hydrogenation process catalyzed by [^*t*^Bu_3_PAuAl(NON)], as shown in Scheme 3 (the full path and structures are reported in Fig. S27 and S28 in the ESI†).

Starting from PC_Au_, hydrogenation of ethylene occurs in a single, exergonic step (−25.6 kcal mol^−1^), *via* a concerted transition state (TS^cat^_Au_, see Fig. S28 in the ESI†), where both hydrogens are simultaneously transferred to the substrate, forming ethane and regenerating the gold–aluminyl catalyst, with a kinetically accessible barrier (21.7 kcal mol^−1^). Given the enhanced stability of PC_Cu_ and PC_Ag_ (thus representing larger thermodynamic sinks), this catalytic mechanism is predicted to be less efficient for copper– and silver–aluminyl complexes. Indeed, as shown in Fig. S27,† while the ethane formation is found to be exergonic for both species (Δ*G* = −15.7 and −20.3 kcal mol^−1^ for Cu and Ag, respectively), the activation barrier is higher for both metals (29.4 and 26.8 kcal mol^−1^ for Cu and Ag, respectively), thus reducing the possible catalytic performance of the copper– and silver–aluminyl complexes.

## Conclusions

In conclusion, computational evidence for the activation of H_2_ by coinage metal–aluminyl complexes *via* a radical pair stabilization mechanism is provided, in close resemblance with experimentally observed dihydrogen activation with group 14 dimetallenes and dimetallynes, and at a variance with the reactivity of general TM–LA complexes. This offers an alternative paradigm for small molecule activation by electron-rich and highly covalent metal–metal bonds which are able to induce a radical-like reactivity. The kinetically accessible and almost thermoneutral formation of product species appears certainly as an ideal condition for the use of the [^*t*^Bu_3_PAuAl(NON)] complex as a catalyst for hydrogenation of, for instance, unsaturated C–C bonds.^47^

This work falls within the sustainable “catalysis by design” as a green key technology in continuing the search for innovative strategies for small molecule activation, and strongly calls for systematic experimental feedback.^48^

## Computational details

Complexes [^*t*^Bu_3_PMAl(NON)] (M = Cu, Ag, Au) have been slightly simplified at the NON site by replacing the two *tert*-butyl groups at the peripheral positions of the dimethylxanthene moiety with hydrogen atoms and the two Dipp substituents on the nitrogen atoms with phenyl groups. The effect of modelling on this class of complexes has been extensively evaluated in ref. 2 and 4 where the same computational set up as that used in the present work was applied. Good agreement with experimental data was found for geometries, and in general, both the reaction mechanism and the electronic structure calculations show negligible deviations due to the structural simplifications used.

All geometry optimizations and frequency calculations on optimized structures (minima with zero imaginary frequencies and transition states with one imaginary frequency) for the H_2_ reaction with the [^*t*^Bu_3_PMAl(NON)] (M = Cu, Ag, Au) complexes have been carried out using the Amsterdam density functional (ADF) code^49,50^ in combination with the related Quantum-regions Interconnected by Local Description (QUILD) program.^51^ The PBE^52^ GGA exchange–correlation (XC) functional, the TZ2P basis set with a small frozen core approximation for all atoms, the ZORA Hamiltonian^53–55^ for treating scalar relativistic effects and the Grimme's D3-BJ dispersion correction were used.^56,57^ Solvent effects were modelled employing the conductor-like screening model (COSMO) with the default parameters for toluene as implemented in the QUILD code.^58^ Effects of the exchange–correlation functional and solvation in this framework have been recently evaluated and are found, overall, to negligibly affect the results.^4^ The effect of different exchange–correlation functionals on the reaction energetics has been also investigated in this work, highlighting an overall marginal impact on both the kinetics and thermodynamics of the reported reactions (see Table S11 and Fig. S29 in the ESI†).

The same computational setup has also been used for the AMS, EDA, CD-NOCV and ETS-NOCV analyses, as well as for the calculation of Voronoi deformation density (VDD)^45^ charges along the reaction paths. This computational protocol has been used in ref. 1 and 2 to study the [^*t*^Bu_3_PAuAl(NON)] and [^*t*^Bu_3_PAuCO_2_Al(NON)] complexes and to investigate the mechanisms of the CO_2_ insertion reaction in similar compounds featuring gold and group 13 elements.^3–5^

The analysis of the transition state TS2 for the reaction of the aluminium–platinum complex with H_2_ has been carried out with the same computational protocol on the geometry optimized in the original work.^21^ Similarly, the radical recombination pathways for the model digermyne compounds have been investigated at the same computational level using the geometries provided in ref. 12.

The transition state theory (TST)^31^ calculations for the evaluation of tunnel effects have been carried out with the kinetic and statistical thermodynamic package (KiSThelP)^59^ computer code. Activation energies have been computed from the rate constant with the three-parameter fitted Arrhenius equation. The non-corrected activation energy (*E*^TST^_a_) has been obtained from fitting of the conventional TST-calculated rate constant, while the tunneling-corrected activation energy (*E*^TST-Eckart^_a_) has been obtained *via* fitting of the TST rate constant corrected with the Eckart tunneling correction.^32^

## Data availability

All the relevant data (including methodology description, and mechanistic, CD-NOCV, ASM, EDA, VDD and TST data discussed in the manuscript) are provided within the ESI.†

## Author contributions

D. S., L. B. and P. B. conceived the project, D. S., L. B. and P. B. performed the research, and D. S., L. B. and P. B. wrote the manuscript.

## Conflicts of interest

There are no conflicts to declare.

## Supplementary Material

SC-014-D2SC05815D-s001
