# Supplementary material for: Radical-like reactivity for dihydrogen activation by coinage metal–aluminyl complexes: computational evidence inspired by experimental main group chemistry
Source: Chem Sci. 2022 Dec 15;14(4):889–96. doi: 10.1039/d2sc05815d (PMC9890964; doi:10.1039/d2sc05815d)
Supplement: SC-014-D2SC05815D-s001 [file SC-014-D2SC05815D-s001.pdf]

Electronic Supplementary Material (ESI) for Chemical Science.
This journal is © The Royal Society of Chemistry 2022

Electronic Supporting Information

Radical-like reactivity for dihydrogen activation by coinage metal-aluminyl complexes: computational evidence inspired by experimental main group chemistry

Diego Sorbelli,[*ab] Leonardo Belpassi,[*b] and Paola Belanzoni[*ab]

[a]*Department of Chemistry, Biology and Biotechnology, University of Perugia, Via Elce di Sotto, 8 – 06123, Perugia, Italy*

[b]*CNR Institute of Chemical Science and Technologies "Giulio Natta" (CNR-SCITEC), Via Elce di Sotto, 8 – 06123, Perugia, Italy*

Contents

Methodology **2**
Figures S1-S4. Optimized structures **7**
Table S1. Tunneling effects **13**
Table S2. EDA results **14**
Figures S5-S16. NOCV breakdown at all TSs **15**
M-Al bond analysis **27**
  Figures S17-S18. NOCV analysis of the Ag-Al bond **27**
  Table S3. CD-NOCV results for the M-Al bond analysis **29**
Table S4. Comparative analysis of the LUMO nature at TS^{I}/TS^{IV} **31**
ASM analysis **32**
  Tables S5-S7. ASM results **32**
Table S8 and Figure S19. Metal contribution to the LUMO of metal-aluminyl complex **34**
Comparative analysis of H_2 activation with the bimetallic Pt-Al complex **35**
  Figure S20. NOCV analysis at TS2 for the Pt-Al complex **35**
  Table S9. Donor MO composition comparison between Au-Al and Pt-Al complexes **36**
Isomerization products **38**
  Scheme S1. Comparative analysis of isomerization products between digermyne and gold-aluminyl compounds **38**
Figures S21-S26. Pathways for heterolytic/homolytic H-H dissociation on products **41**
Table S10. VDD charges evolution **47**
Figure S27. Free energy profile for the catalytic hydrigenation of ethylene **48**
Figure S28. Optimized structures for the catalytic hydrogenation of ethylene **51**
Figure S29 and Table S11. DFT benchmark on reaction energies **52**
References **54**
xyz geometries **55**

Methodology

- **Natural Orbitals for Chemical Valence and Charge Displacement analysis**

Natural Orbitals for Chemical Valence (NOCV)[1,2] represents a suitable approach for describing the chemical bond. This approach is based on the rearrangement of the electron density occurring when a chemical bond is formed and such rearrangement can be expressed as electron density difference between the formed adduct (AB) and sum of the densities of the two non-interacting fragments (A and B) frozen in their adduct geometry.

This deformation density can be brought into diagonal contributions in terms of NOCVs. In the NOCV scheme, the charge rearrangement taking place upon bond formation is obtained from the occupied orbitals of the two fragments suitably orthogonalized to each other and renormalized (*promolecule*). The resulting electron density rearrangement ($\Delta\rho'$) can be expressed in terms of NOCV pairs which are defined as the eigenfunctions of the so-called "valence operator"[3,4] as follows:

$$\Delta\rho' = \sum_k \nu_k(|\phi_{+k}|^2 - |\phi_{-k}|^2) = \sum_k \Delta\rho'_k \qquad \text{[S1]}$$

where ϕ_{+k} and ϕ_{-k} are the NOCV pairs orbitals and $\nu_{\pm k}$ are the corresponding eigenvalues. Upon formation of the adduct from the promolecule, a fraction ν_k of electrons is transferred from the ϕ_{-k} to the ϕ_{-k} orbital (donor and acceptor orbitals, respectively).

The NOCV scheme can be coupled with the framework of the Charge Displacement (CD)[5] analysis. The CD analysis allows to quantify the amount of electronic charge that is transferred between the two fragments upon the formation of the A-B bond. The Charge Displacement function (Δq) can be defined as the partial progressive integration on a suitable z-axis of the deformation density $\Delta\rho'$:[6]

$$\Delta q(z) = \int_{-\infty}^{z} dz' \int_{-\infty}^{+\infty}\int_{-\infty}^{+\infty} \Delta\rho'(x,y,z')\,dx\,dy \qquad \text{[S2]}$$

The CD function, $\Delta q(z)$, quantifies at each point of the chosen z-axis (which usually corresponds to the bond axis) the exact amount of electron charge that, upon formation of the bond, is transferred from the right to the left across a plane perpendicular to the bond axis through z.

When coupled with the NOCV scheme, the density rearrangement due to the bond formation between two fragments, ($\Delta\rho'$), is partitioned in different NOCV deformation densities ($\Delta\rho'_k$) and therefore one is able to quantify the charge transfer (CT) associated to the components. Note that only few of the

NOCV pairs contributes to the chemical bond. Therefore, when the CD-NOCV analysis is carried out, usually only the first $\Delta\rho_k'$ components are investigated in order to understand which significant chemical contribution to the bond they represent.

Usually we choose to evaluate the charge transfer between A and B by taking the CD value at the "isodensity boundary", i.e. the z-point where equally valued isodensity surfaces of the isolated fragments become tangent.[6,7]

When we apply this scheme at TSI with [^{t}Bu$_3$PAuAl(NON)] and [H_2] as fragments, such approach becomes complicated, since the two fragments display multiple interactions with multiple atomic centres and thus it is clearly impossible to define a unique bond axis and it is very hard to rely on the isodensity boundary for the estimation of the charge transfer. In order to avoid any ambiguity in the definition of the z-axis, we recall an approach that may be useful for evaluating the charge transferred between the [^{t}Bu$_3$PAuAl(NON)] and [H_2] fragments at TSI.[8]

Within this approach, the electron density rearrangement ($\Delta\rho'$), which typically shows charge accumulation regions (positive values) and charge depletion regions (negative values), defines two different positive functions, $\Delta\rho^+$ and $\Delta\rho^-$, each equal to the magnitude of the appropriate portion, *i.e.*:

$$\Delta\rho^{+/-}(r) = max[\pm\Delta\rho(r)', 0] \quad \text{[S3]}$$

so that

$$\Delta\rho(r)' = \Delta\rho^+(r) - \Delta\rho^-(r) \quad \text{[S4]}$$

By defining two arbitrary regions that are associated with the interacting fragments, we can evaluate the charge transfer as follows:

$$CT = \int_A \Delta\,\rho(r)'dr = -\int_B \Delta\,\rho(r)'dr \quad \text{[S5]}$$

By combining Eqs. [S4] and [S5], CT can also be expressed as:

$$CT = \int_A \Delta\,\rho^+(r)dr - \int_A \Delta\,\rho^-(r)dr = -\int_B \Delta\,\rho^+(r)dr + \int_B \Delta\,\rho^-(r) \quad \text{[S6]}$$

Ultimately, this approach can also be expressed in the CD-NOCV framework. By combining Equations [S1] and [S5], we can use to this approach for calculating the charge transfer associated to each NOCV deformation density as follows:

$$CT_k = \int_A \Delta\, \rho_k(r)' dr = -\int_B \Delta\, \rho_k(r)' dr \quad [S7]$$

Despite the spatial regions associated to the two interacting fragments being defined arbitrarily, this approach is particularly suitable for the analysis of the interaction between the [^{t}Bu$_3$PAuAl(NON)] and [H_2] fragments at TSI, being the two fragments well-separated in space.

- **Energy Decomposition Analysis and ETS-NOCV approach**

The Energy Decomposition Analysis (EDA)[9–11] has been used in this work to get additional and complementary insights into the interaction between dihydrogen and the [^{t}Bu$_3$PAuAl(NON)] complex in the transition state TSI. With this approach, the interaction energy between the fragments can be decomposed in different contributions as follows:

$$\Delta E_{\text{int}} = \Delta E^{Pauli} + \Delta V_{elst} + \Delta E_{oi} + \Delta E_{disp} \quad [S8]$$

where ΔE^{Pauli} corresponds the Pauli repulsion interaction between occupied orbitals on the two fragments, ΔV_{elst} represents the quasiclassical electrostatic interaction between the unperturbed charge distribution of the fragments at their final positions, ΔE_{disp} takes into account the dispersion contribution and ΔE_{oi} is the orbital interaction, which arises from the orbital relaxation and the orbital mixing between the fragments, and accounts for electron pair bonding, charge transfer, and polarization.

The orbital interaction term ΔE_{oi} can be further decomposed within the ETS-NOCV[12] scheme into NOCV pairwise orbital contributions ($\Delta E_{oi} = \sum_k \Delta\, E_{oi}^k$) which associates an energy contribution (E_{oi}^k) to each NOCV deformation density ($\Delta\rho_k$).

- **Activation Strain Model**

The Activation Strain Model (ASM)[13–15] allows to decompose the relative energy (ΔE) of each stationary point along the reaction path in two contributions: a penalty arising from the distortion of

the reactants from their relaxed geometry to their in-adduct ones (ΔE_{dist}) and a (usually) stabilizing interaction contributions arising from the interaction between reacting fragments (ΔE_{int}). Thus, ΔE can be expressed as follows:

$$\Delta E(\xi) = \Delta E_{\mathrm{dist}}(\xi) + \Delta E_{\mathrm{int}}(\xi) \quad \text{[S9]}$$

where ξ represents the reaction coordinate. In the case of the two reactants (and interacting fragments) being the gold-aluminyl complex and H_2, the distortion penalty can be expressed as:

$$\begin{gathered} \Delta E_{dist}(\xi) = \Delta E_{\mathrm{dist}}^{H_2}(\xi) + \Delta E_{dist}^{complex}(\xi) \\ \Delta E_{\mathrm{dist}}^{H_2}(\xi) = E^{H_2}(\xi) - E_{\mathrm{relaxed}}^{H_2} \\ \Delta E_{dist}^{complex}(\xi) = E^{complex}(\xi) - E_{\mathrm{relaxed}}^{complex} \end{gathered} \quad \text{[S10]}$$

where the "$E^{H2}(\xi)$" and "$E^{complex}(\xi)$" terms represent the energy of the two fragments evaluated at the geometry assumed by the fragments at selected points along the reaction coordinate, which in this work will be the stationary points (*i.e.* RC/RC', TS/TS' and PC). Similarly, the interaction contribution can be expressed as:

$$\Delta E_{\mathrm{int}}(\xi) = E(\xi) - E^{complex}(\xi) - E^{H_2}(\xi) \quad \text{[S11]}$$

where "$E\,(\xi)$" represents the energy of the stationary point under study.

Clearly, in the case of the interaction between the complex and NH_3, an analogous formalism applies for the analysis of the related reaction path.

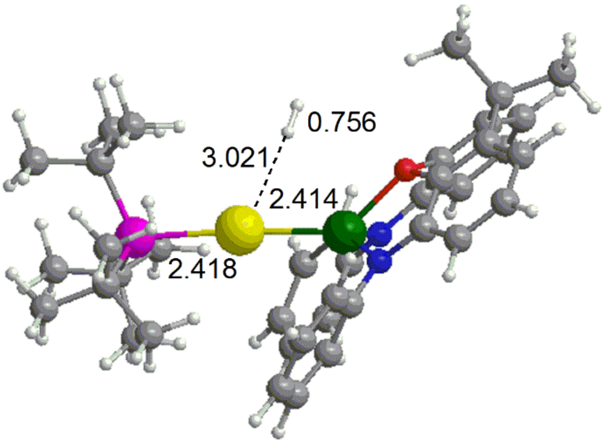

RC_{Au}

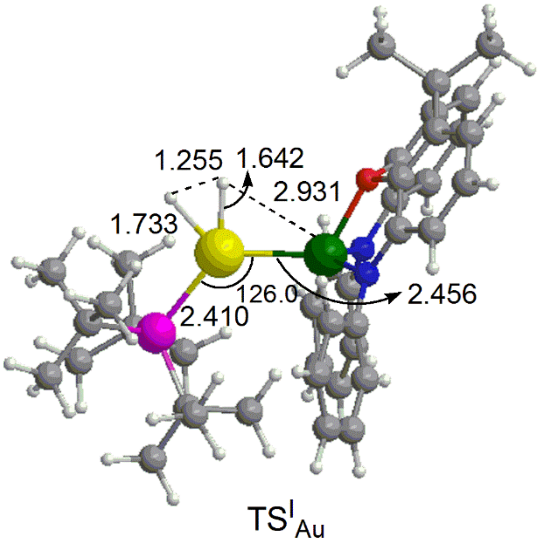

TS^{I}_{Au}

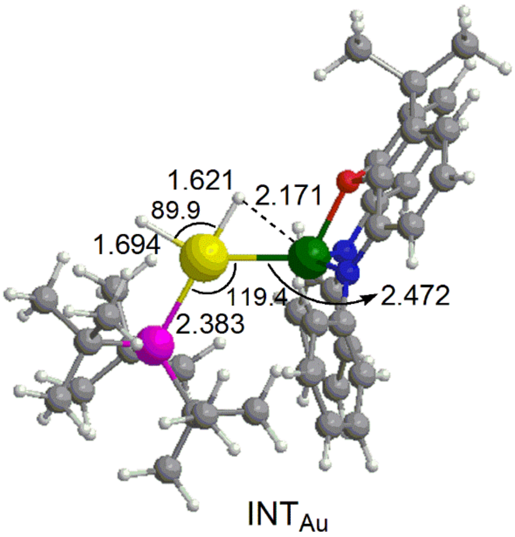

INT_{Au}

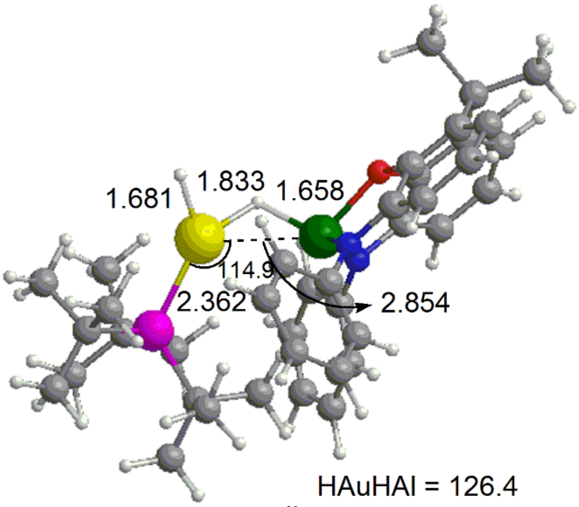

TS^{II}_{Au}

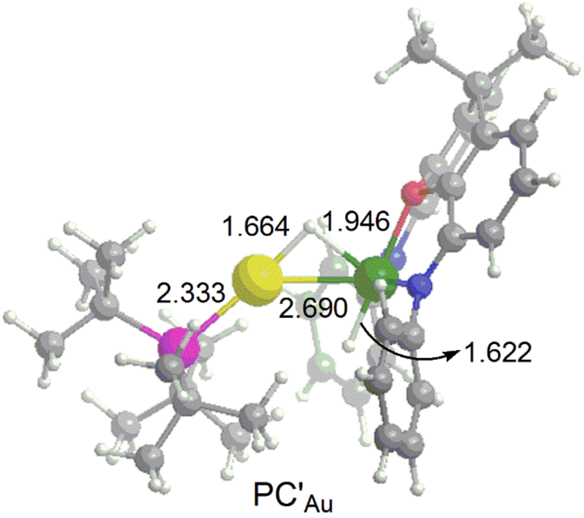

PC'_{Au}

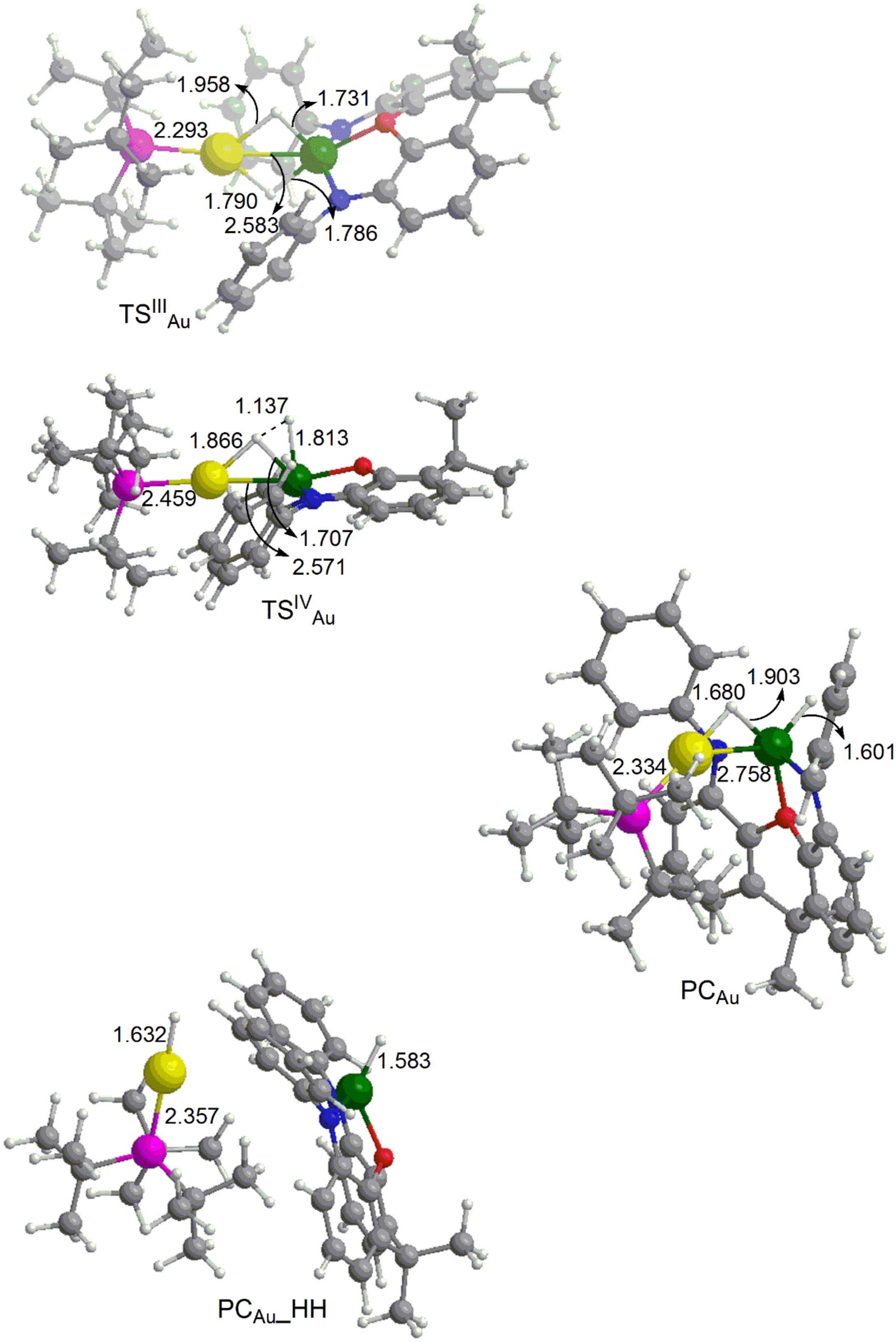

Figure S1. Optimized structures of **RC$_{Au}$**, **TS$^{I}_{Au}$**, **INT$_{Au}$**, **TS$^{II}_{Au}$**, **PC'$_{Au}$**, **TS$^{III}_{Au}$**, **TS$^{IV}_{Au}$**, **PC$_{Au}$** and **PC$_{Au}$_HH** for [^{t}Bu$_3$PAuAl(NON)] complex. Main geometrical parameters are reported (bond in Å, angles in degree). Species **PC$_{Au}$_HH** refers to the product complex formed by two hydride complexes (ΔG = 10.2 kcal/mol above the separated reactants taken as zero energy, see Figure 1 in the main text).

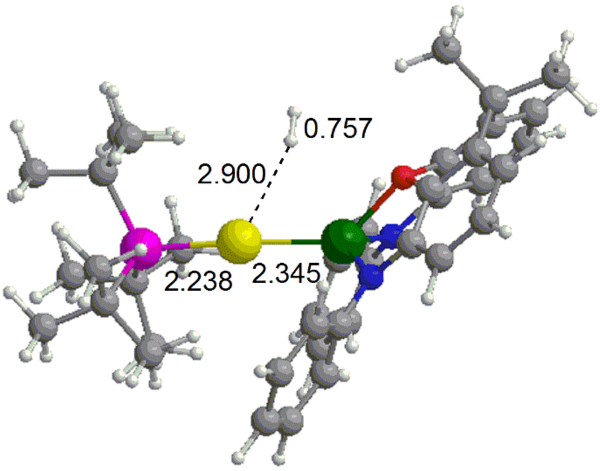

RC_{Cu}

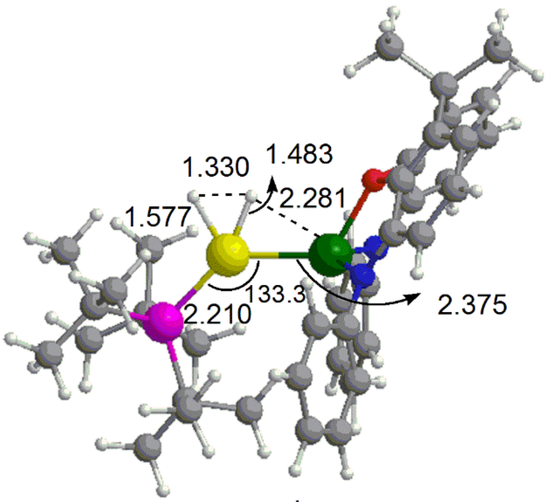

TS^{I}_{Cu}

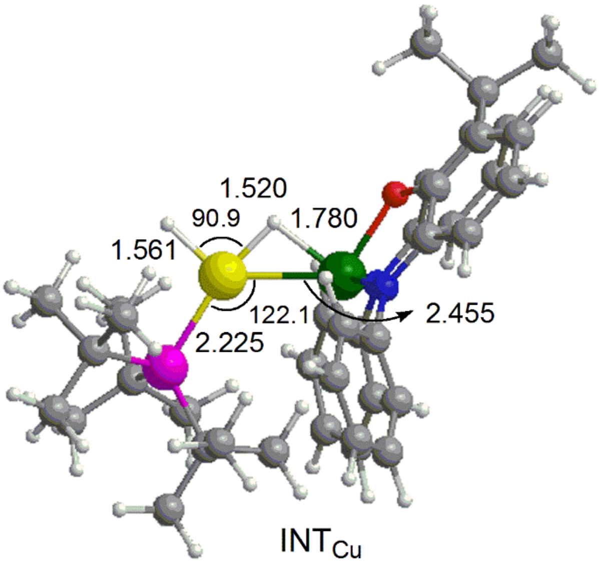

INT_{Cu}

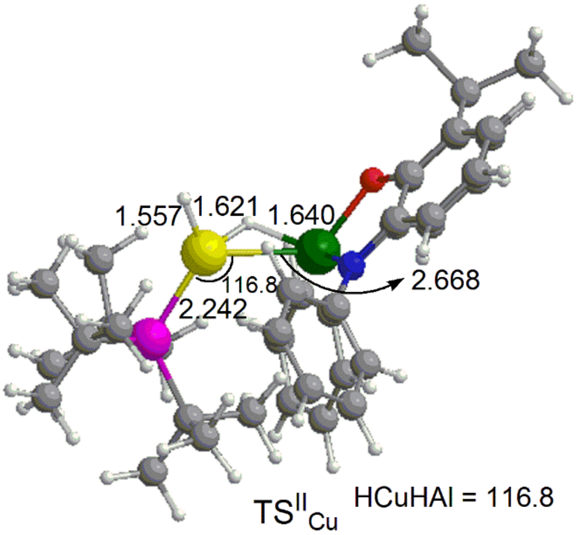

TS^{II}_{Cu} HCuHAl = 116.8

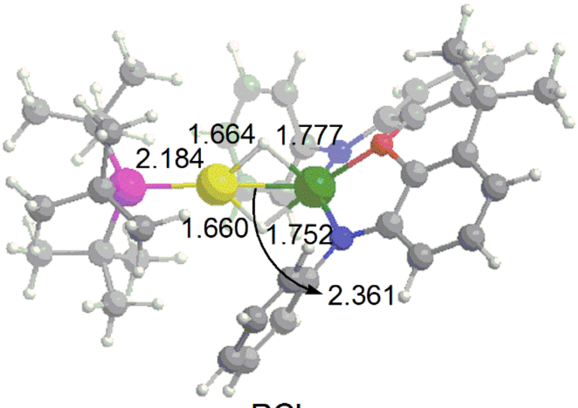

PC'_{Cu}

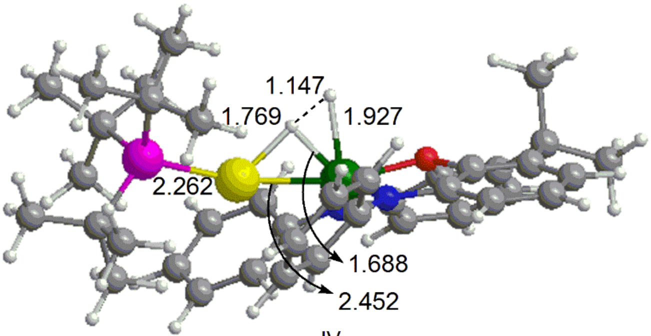

TS^{IV}_{Cu}

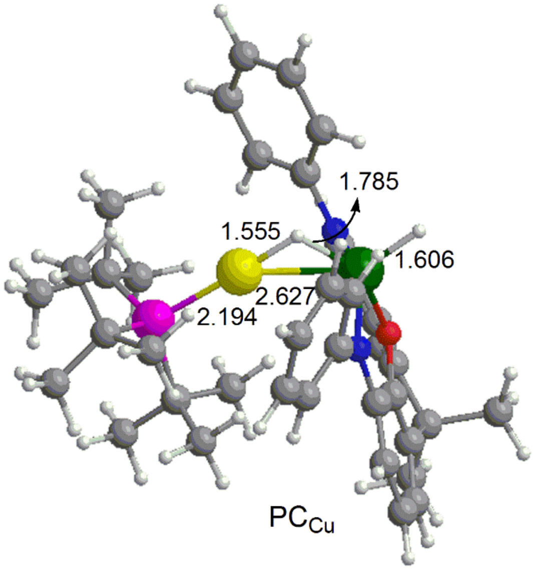

PC_{Cu}

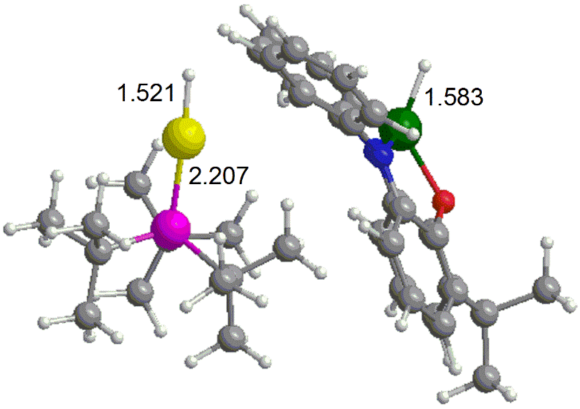

PC_{Cu}_HH

Figure S2. Optimized structures of **RC_{Cu}**, **TS^{I}_{Cu}**, **INT_{Cu}**, **TS^{II}_{Cu}**, **PC'_{Cu}**, **TS^{IV}_{Cu}**, **PC_{Cu}** and **PC_{Cu}_HH** for [$^{t}Bu_3PCuAl(NON)$] complex. Main geometrical parameters are reported (bond in Å, angles in degree). Species **PC_{Cu}_HH** refers to the product complex formed by two hydride complexes (ΔG = 7.9 kcal/mol above the separated reactants taken as zero energy, see Figure 2 in the main text).

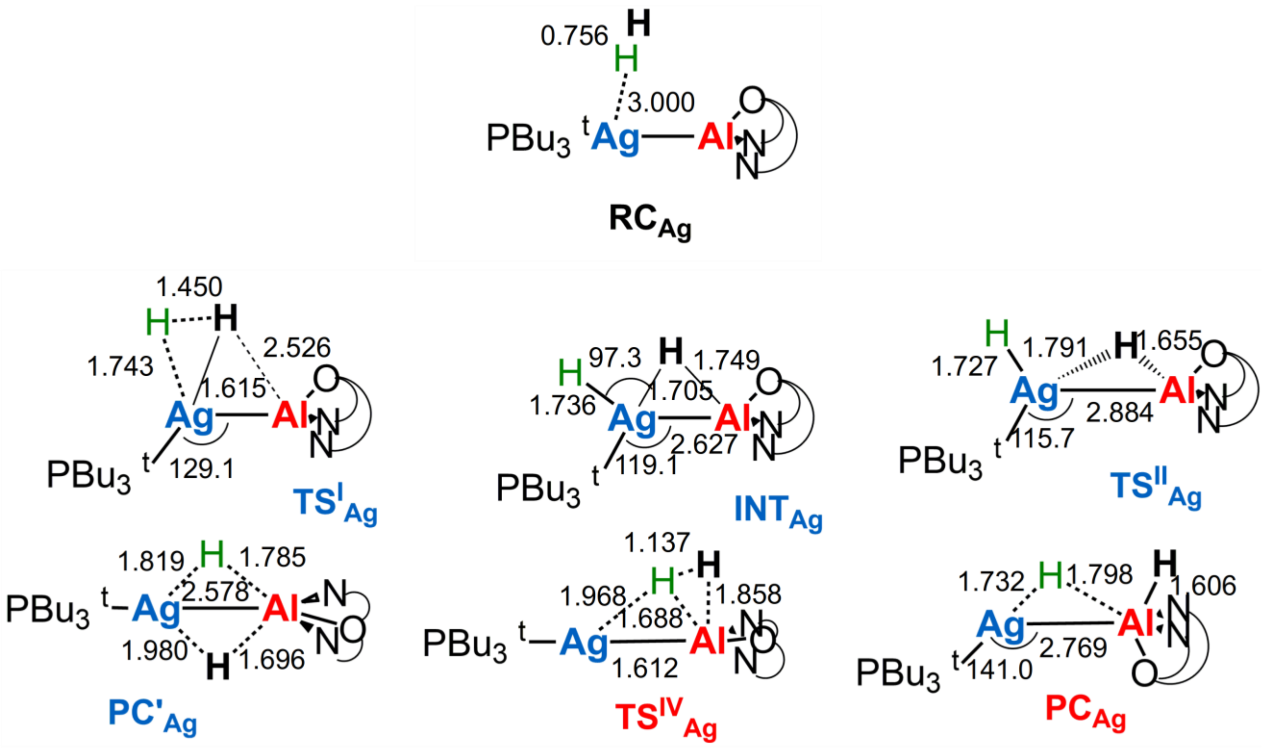

Figure S3. Sketched geometrical structures with the most relevant bond lengths (Å) and angles (degrees) of all stationary points for the reaction of [$^{t}Bu_3PAgAl(NON)$] with H_2 (see Figure 2 in the main text).

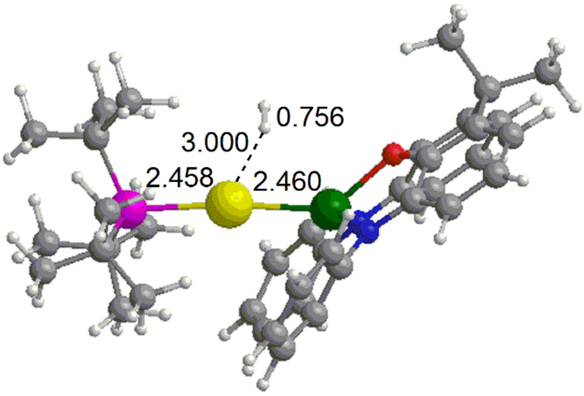

RC_{Ag}

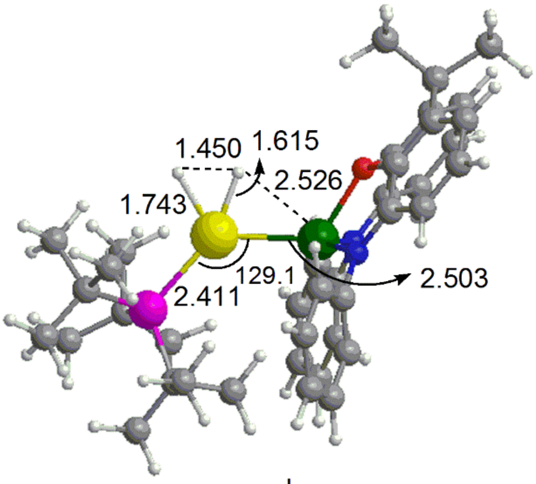

TS^{I}_{Ag}

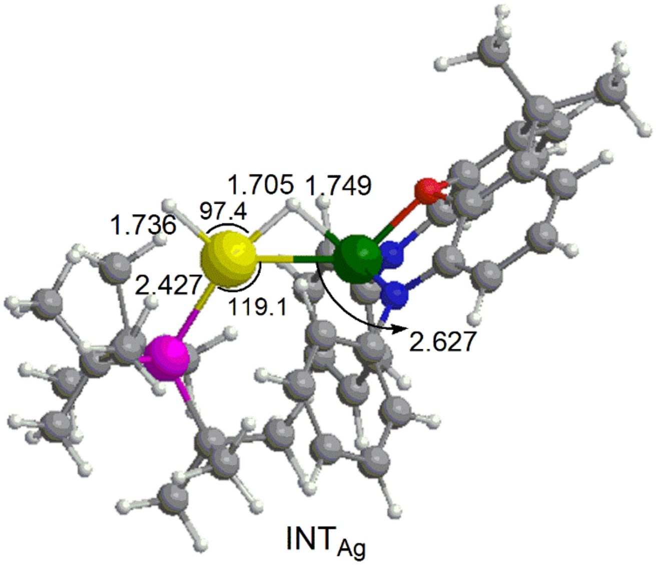

INT_{Ag}

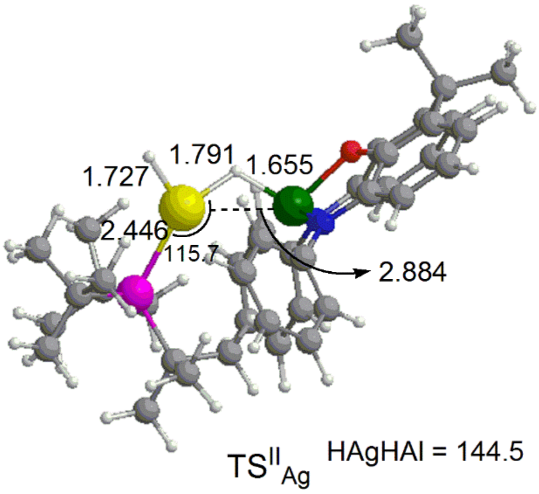

TS^{II}_{Ag} HAgHAl = 144.5

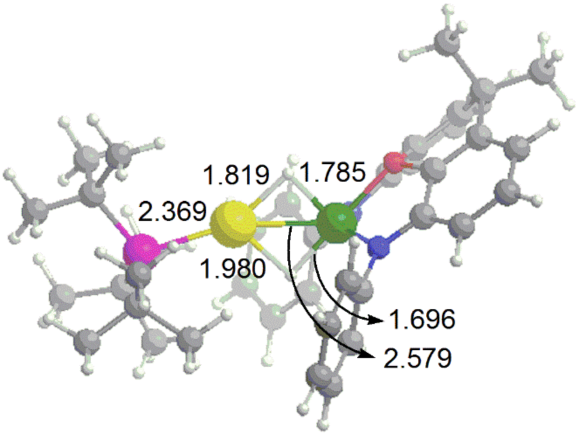

PC'_{Ag}

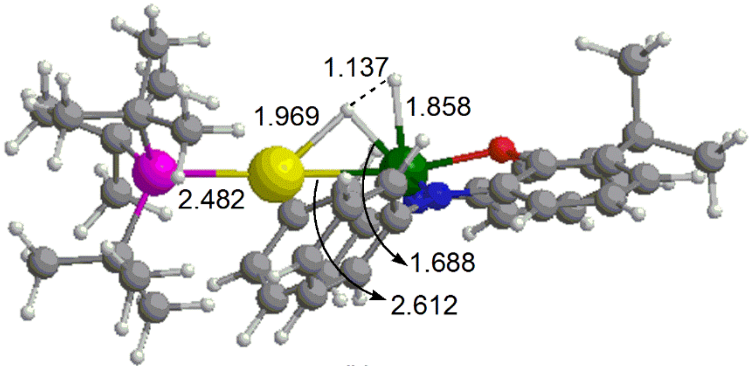

TS^{IV}_{Ag}

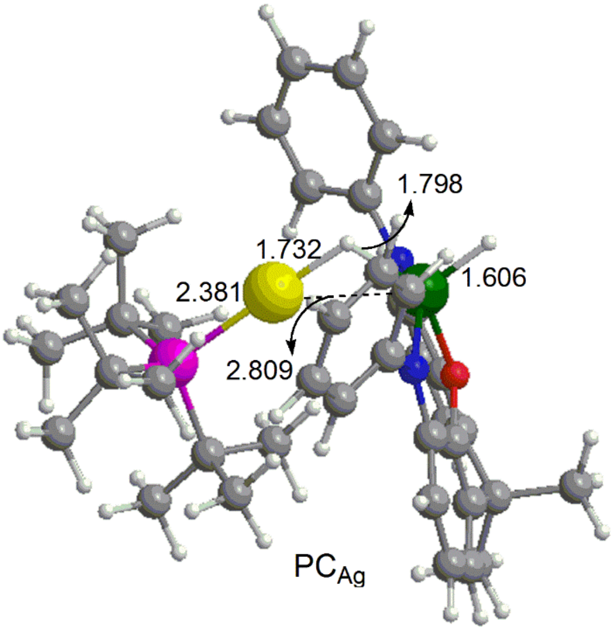

PC_{Ag}

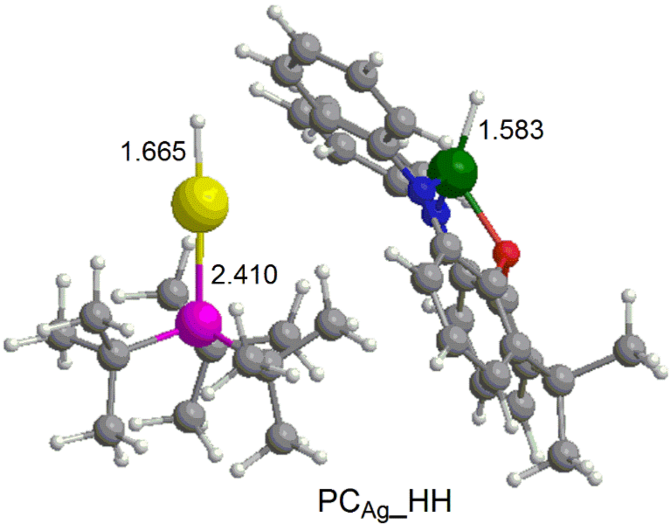

PC_{Ag}_HH

Figure S4. Optimized structures of **RC_{Ag}**, **TS^{I}_{Ag}**, **INT_{Ag}**, **TS^{II}_{Ag}**, **PC'_{Ag}**, **TS^{IV}_{Ag}**, **PC_{Ag}** and **PC_{Ag}_HH** for [$^{t}Bu_3PAgAl(NON)$] complex. Main geometrical parameters are reported (bond in Å, angles in degree). Species **PC_{Ag}_HH** refers to the product complex formed by two hydride complexes (ΔG = 10.0 kcal/mol above the separated reactants taken as zero energy, see Figure 2 in the main text).

	TS^{I}_{Cu}	TS^{I}_{Ag}	TS^{I}_{Au}
**E_a^{TST}**	8.6	24.8	19.6
**$E_a^{TST\text{-}Eckart}$**	6.7	21.1	18.3
**ΔE_a**	1.9	3.7	1.3

Table S1. Activation energies associated to the TS^{I}_{M} transition states (M = Cu, Ag, Au) calculated by application of conventional Transition State Theory (TST) and three-parameter fir Ahrrenius equation not including (E_a^{TST}) and including ($E_a^{TST\text{-}Eckart}$) the Eckart correction for tunneling. All energies are reported in kcal/mol

	TS^{IV}_{Cu}	TS^{I}_{Cu}	TS^{IV}_{Ag}	TS^{I}_{Ag}	TS^{IV}_{Au}	TS^{I}_{Au}
ΔE_{Pauli}	190.7	192.2	192.8	199.6	205.1	222.9
ΔE_{elst}	-74.6	-126.8	-74.8	-138.2	-83.4	-154.8
ΔE_{steric}	116.1	65.4	118.0	61.4	121.7	68.1
ΔE_{oi}	-125.8	-116.2	-126.0	-108.6	-133.0	-107.2
ΔE_{oi}^{1}	-102.7	-79.1	-102.7	-75.1	-108.3	-59.7
$\lvert CT^{1}\rvert$	0.23	0.29	0.21	0.31	0.18	0.18
ΔE_{oi}^{2}	-18.9	-22.4	-19.2	-20.5	-19.2	-27.9
$\lvert CT^{2}\rvert$	0.09	0.13	0.07	0.13	0.07	0.12
ΔE_{disp}	-2.7	-2.2	-2.3	-2.0	-2.3	-2.0
ΔE	-12.5	-53.0	-10.3	-49.2	-13.6	-41.1

Table S2. Results of the Energy Decomposition Analysis (EDA), ETS-NOCV and CD-NOCV analyses of the $[H_2]$-$[^{t}Bu_3PMAl(NON)]$ (M = Cu, Ag, Au) interaction at the corresponding $\mathbf{TS^{I}_{M}}$ and $\mathbf{TS^{IV}_{M}}$. All energies are expressed in kcal/mol, charge transfer (|CT|) values are in electrons.

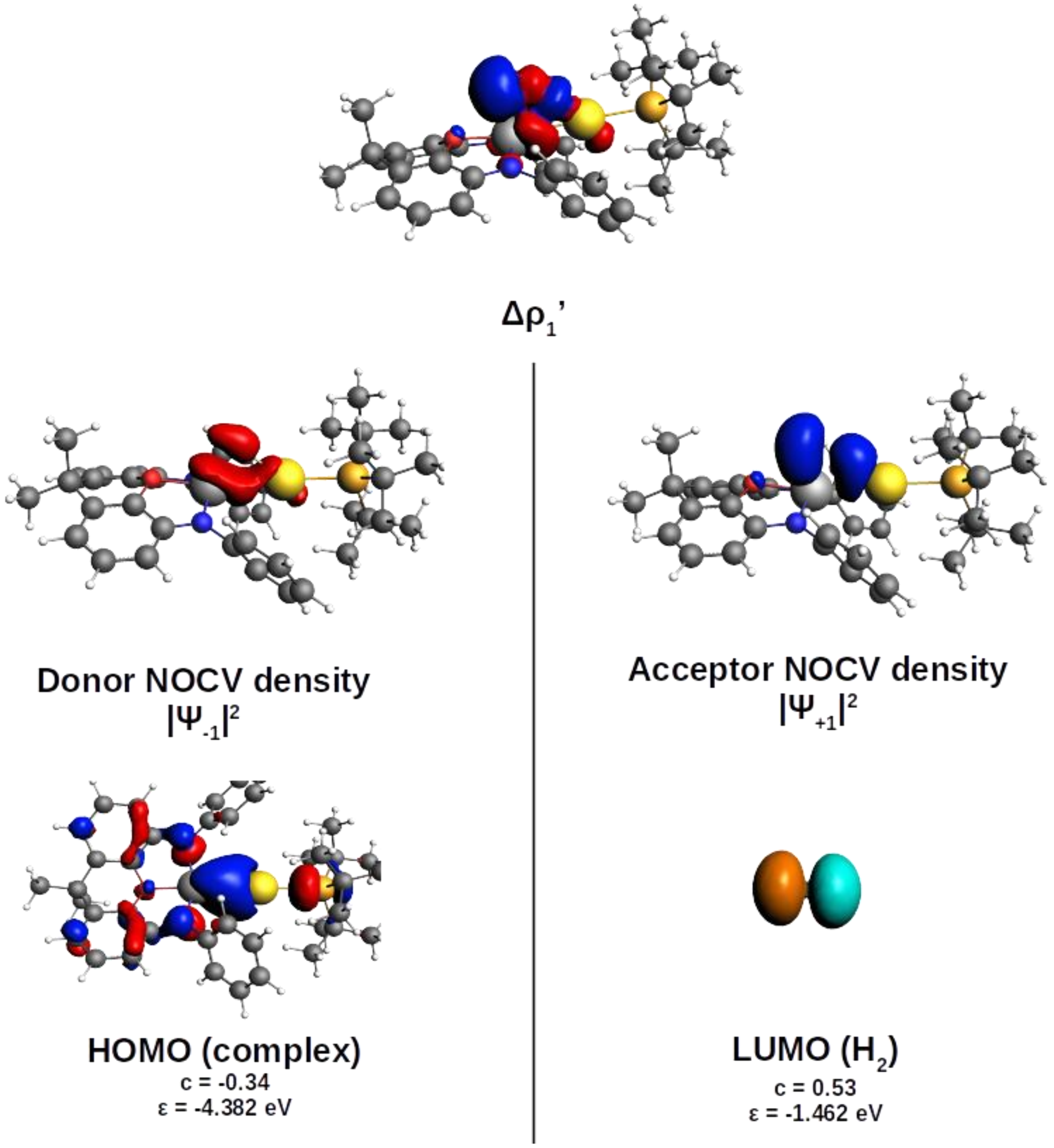

Figure S5. Breakdown of the donor ($|\Psi_1|^2$) and acceptor ($|\Psi_{-1}|^2$) NOCV densities that are associated with the deformation density $\Delta\rho'_1$ in the transition state $\mathbf{TS^{IV}_{Au}}$ into the most important MOs of the fragments frozen at their $\mathbf{TS^{IV}_{Au}}$ geometry. The molecular orbitals' mixing coefficients (c) are given together with their energy (ε).

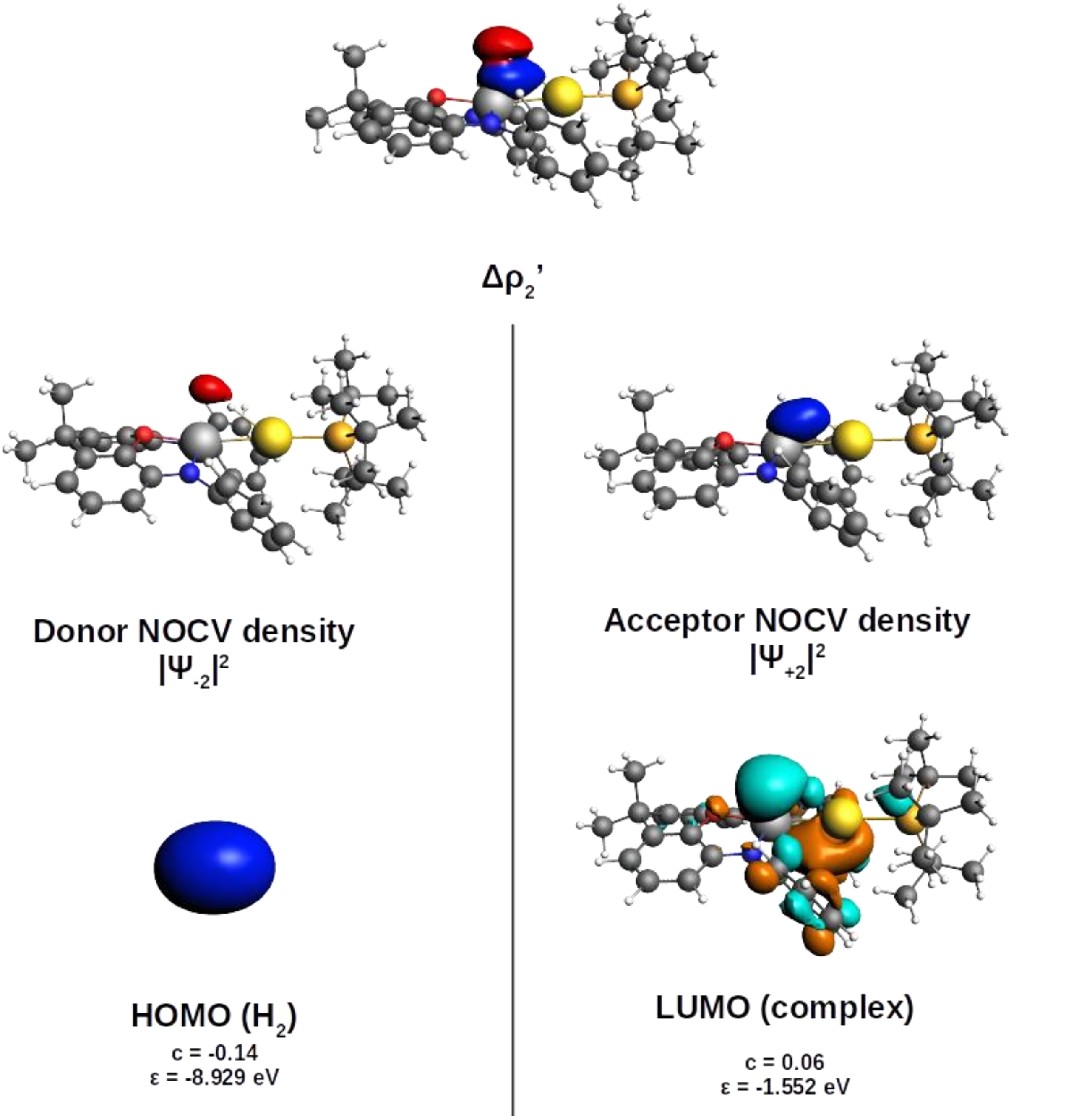

Figure S6. Breakdown of the donor ($|\Psi_2|^2$) and acceptor ($|\Psi_{-2}|^2$) NOCV densities that are associated with the deformation density $\Delta\rho'_2$ in the transition state $\mathbf{TS^{IV}_{Au}}$ into the most important MOs of the fragments frozen at their $\mathbf{TS^{IV}_{Au}}$ geometry. The molecular orbitals' mixing coefficients (c) are given together with their energy (ε).

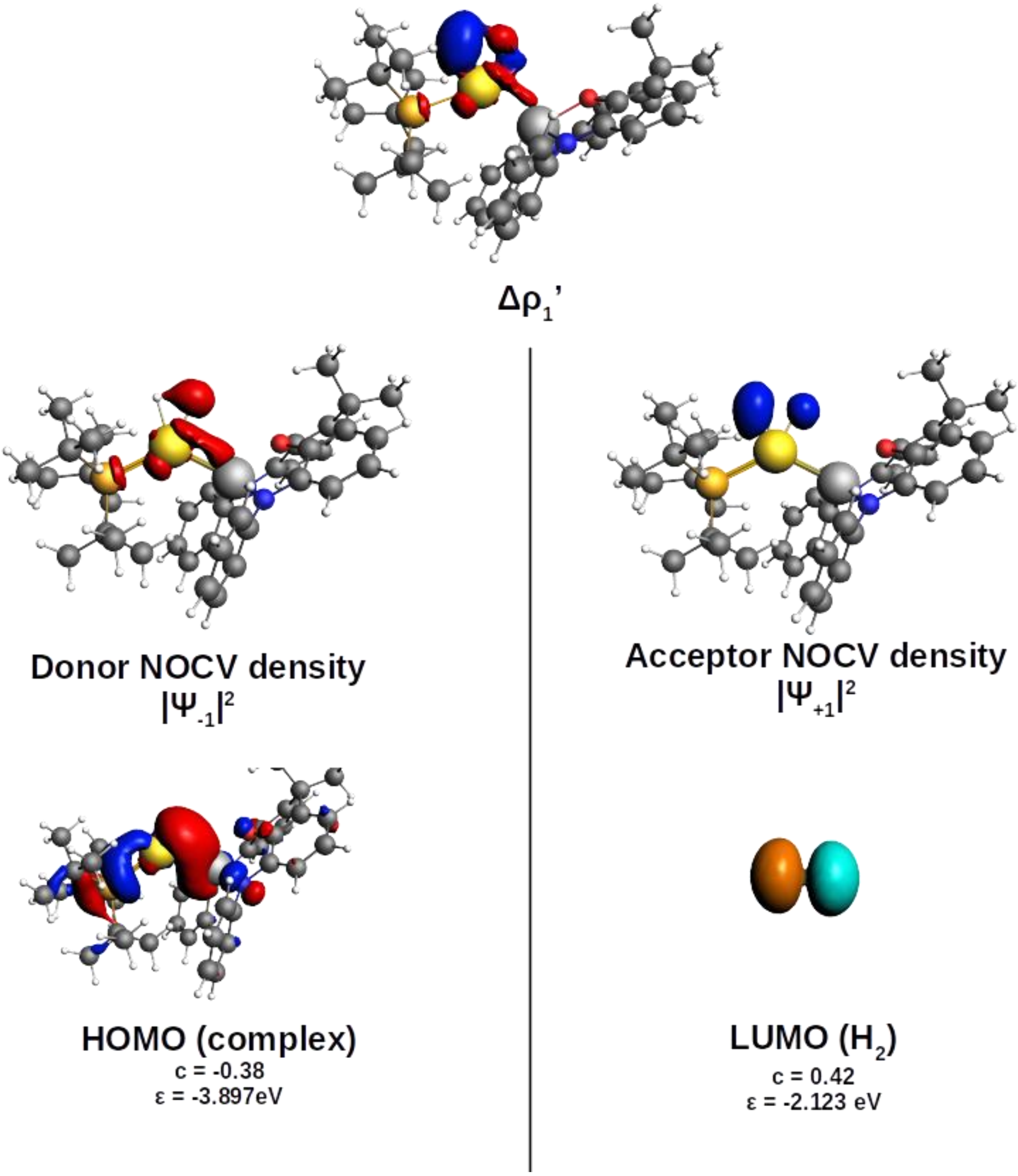

Figure S7. Breakdown of the donor ($|\Psi_1|^2$) and acceptor ($|\Psi_{-1}|^2$) NOCV densities that are associated with the deformation density $\Delta\rho'_1$ in the transition state $\mathbf{TS^{I}_{Au}}$ into the most important MOs of the fragments frozen at their $\mathbf{TS^{I}_{Au}}$ geometry. The molecular orbitals' mixing coefficients (c) are given together with their energy (ε).

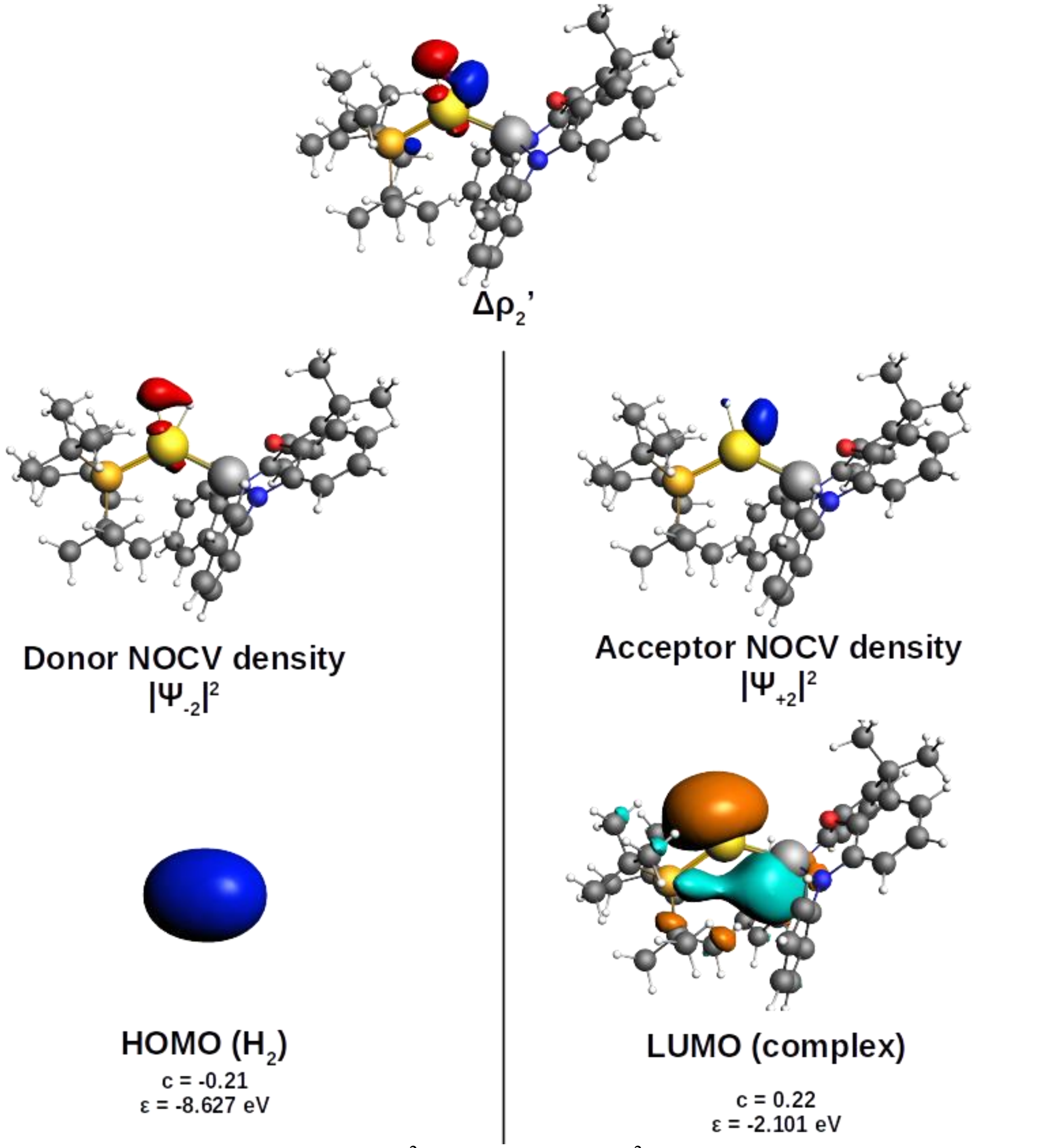

Figure S8. Breakdown of the donor ($|\Psi_2|^2$) and acceptor ($|\Psi_{-2}|^2$) NOCV densities that are associated with the deformation density $\Delta\rho'_2$ in the transition state **TS$^{I}_{Au}$** into the most important MOs of the fragments frozen at their **TS$^{I}_{Au}$** geometry. The molecular orbitals' mixing coefficients (c) are given together with their energy (ε).

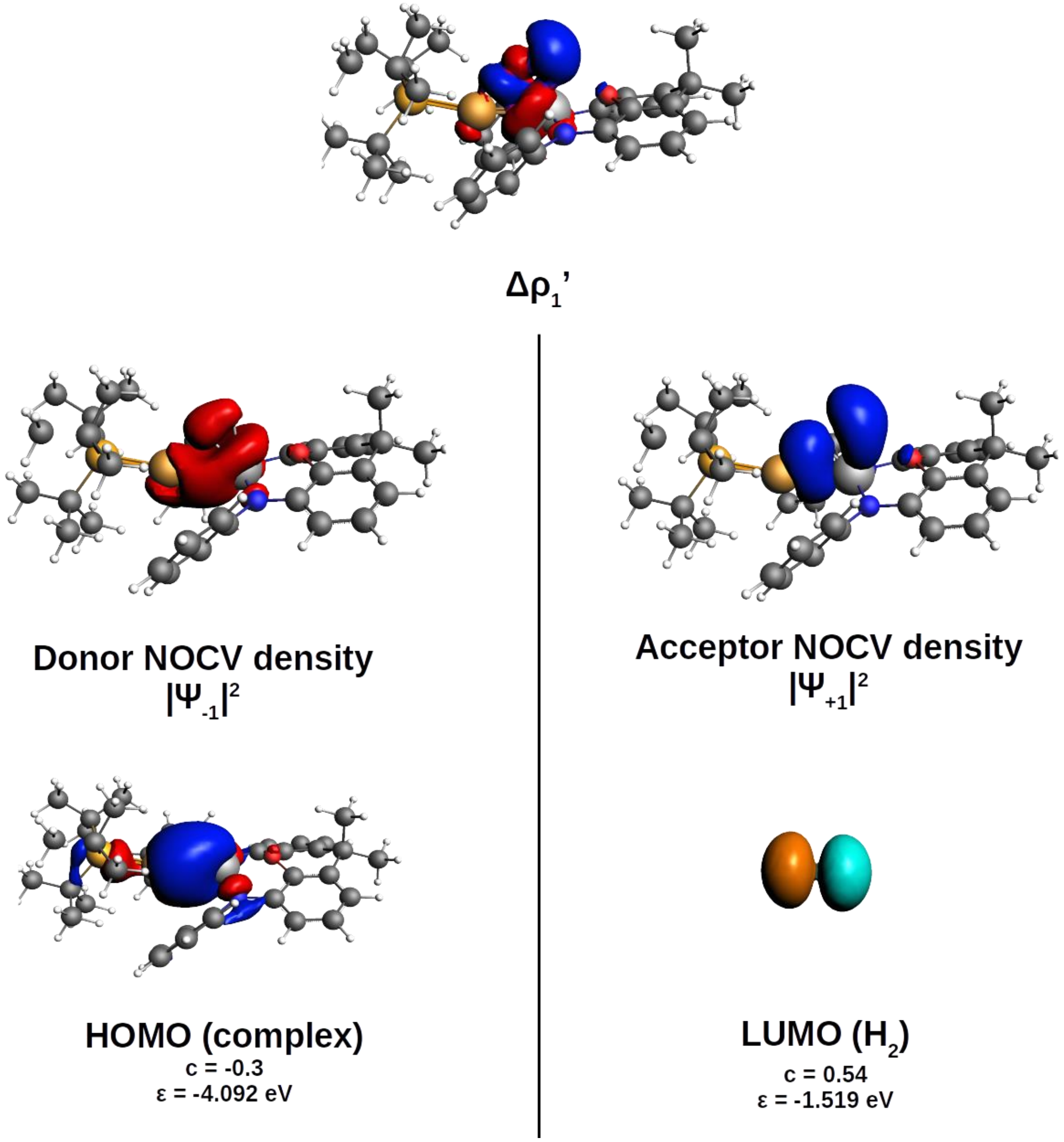

Figure S9. Breakdown of the donor ($|\Psi_{1}|^2$) and acceptor ($|\Psi_{-1}|^2$) NOCV densities that are associated with the deformation density $\Delta\rho'_1$ in the transition state $\mathbf{TS^{IV}_{Cu}}$ into the most important MOs of the fragments frozen at their $\mathbf{TS^{IV}_{Cu}}$ geometry. The molecular orbitals' mixing coefficients (c) are given together with their energy (ε).

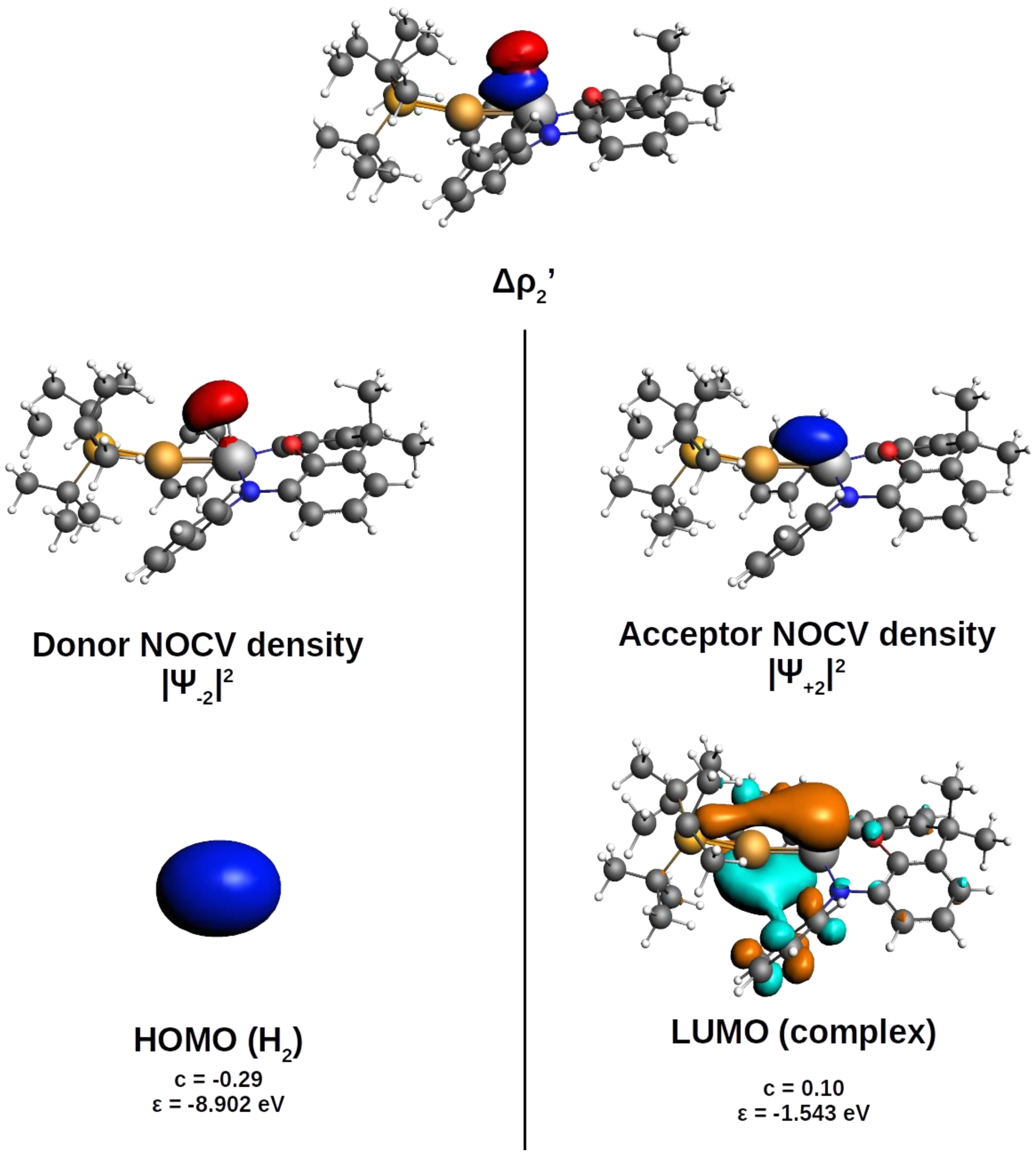

Figure S10. Breakdown of the donor ($|\Psi_2|^2$) and acceptor ($|\Psi_{-2}|^2$) NOCV densities that are associated with the deformation density $\Delta\rho'_2$ in the transition state $\mathbf{TS^{IV}_{Cu}}$ into the most important MOs of the fragments frozen at their $\mathbf{TS^{IV}_{Cu}}$ geometry. The molecular orbitals' mixing coefficients (c) are given together with their energy (ε).

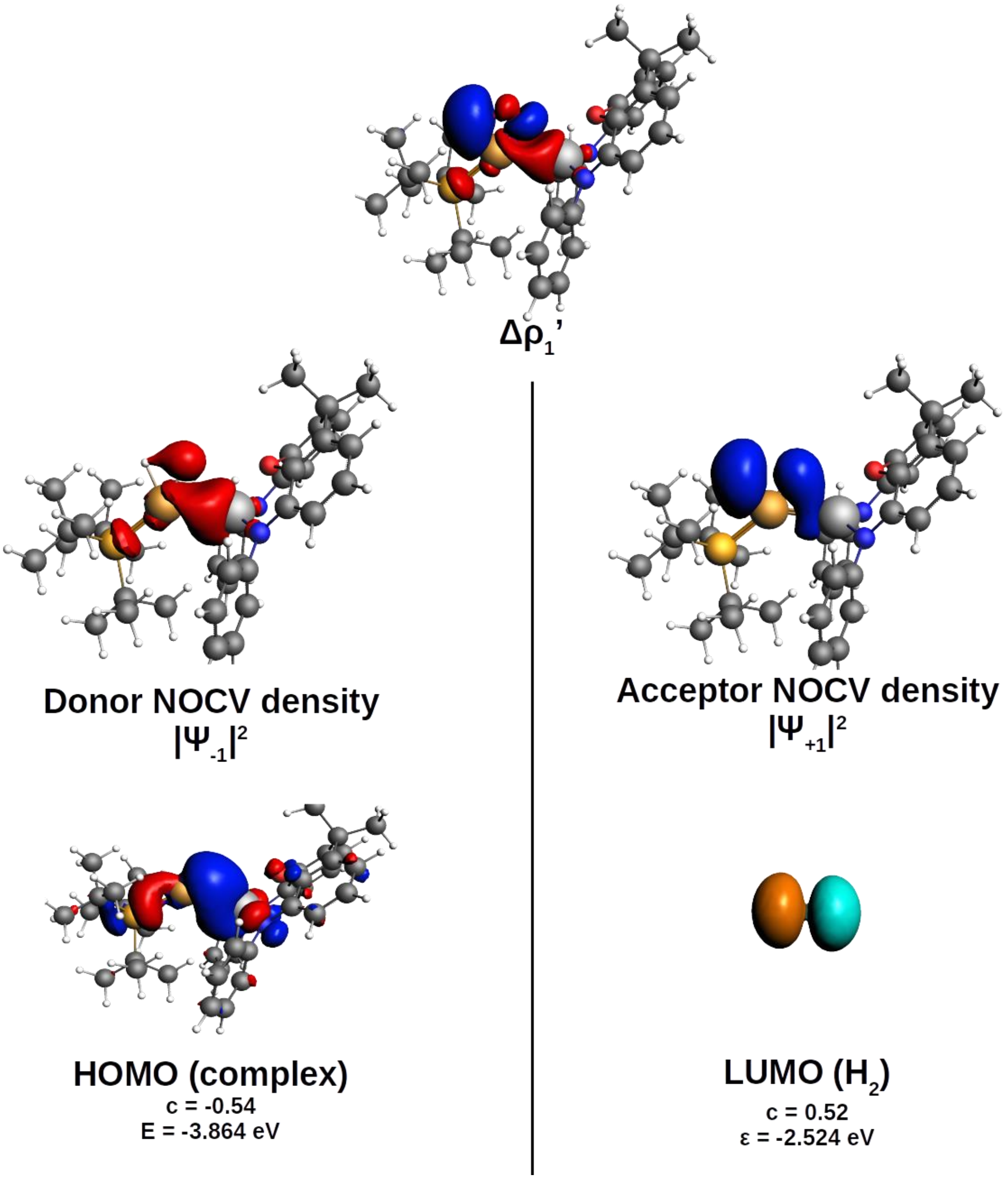

Figure S11. Breakdown of the donor ($|\Psi_1|^2$) and acceptor ($|\Psi_{-1}|^2$) NOCV densities that are associated with the deformation density $\Delta\rho'_1$ in the transition state $\mathbf{TS^I_{Cu}}$ into the most important MOs of the fragments frozen at their $\mathbf{TS^I_{Cu}}$ geometry. The molecular orbitals' mixing coefficients (c) are given together with their energy (ε).

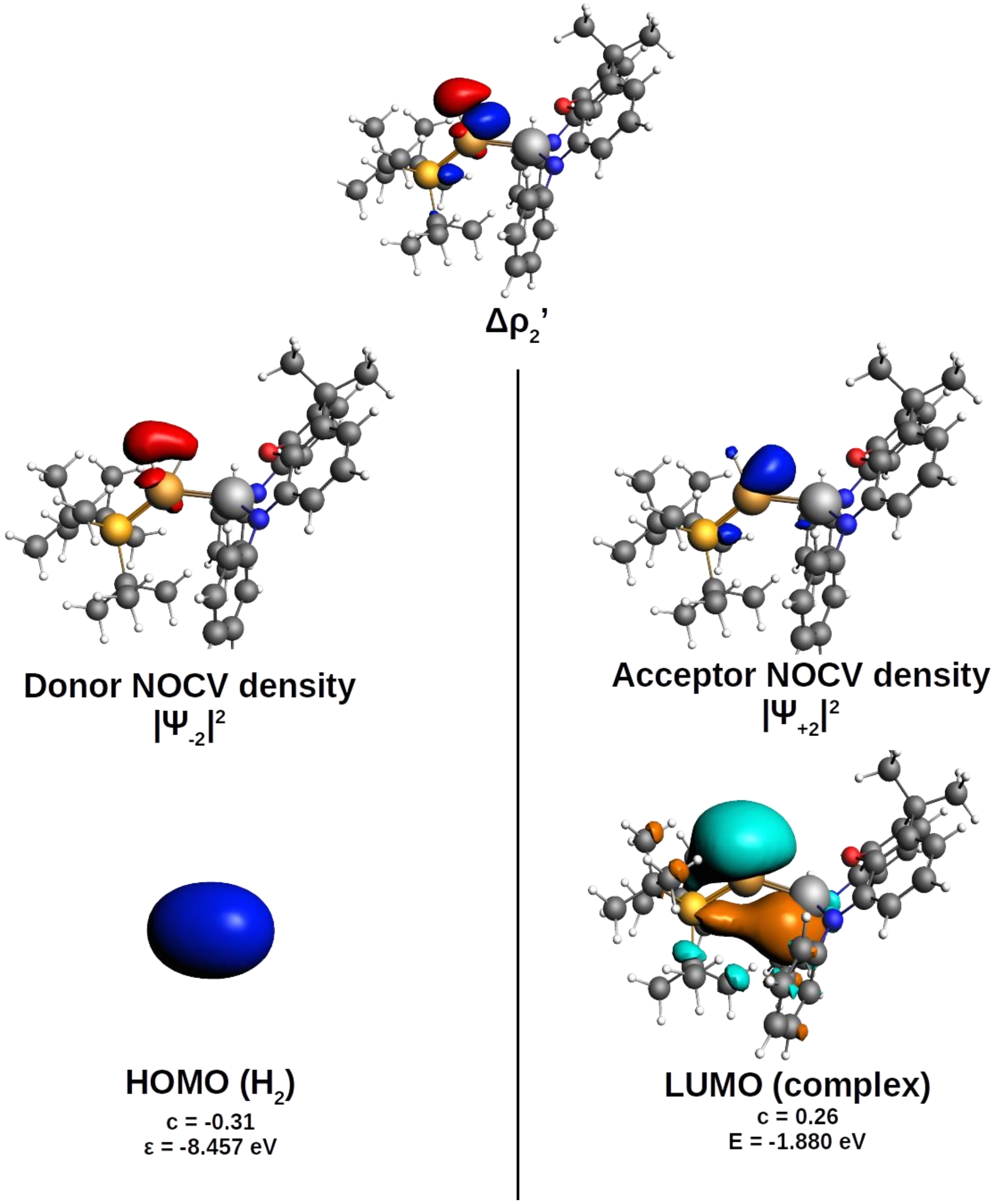

Figure S12. Breakdown of the donor ($|\Psi_2|^2$) and acceptor ($|\Psi_{-2}|^2$) NOCV densities that are associated with the deformation density $\Delta\rho'_2$ in the transition state $\mathbf{TS^{I}_{Cu}}$ into the most important MOs of the fragments frozen at their $\mathbf{TS^{I}_{Cu}}$ geometry. The molecular orbitals' mixing coefficients (c) are given together with their energy (ε).

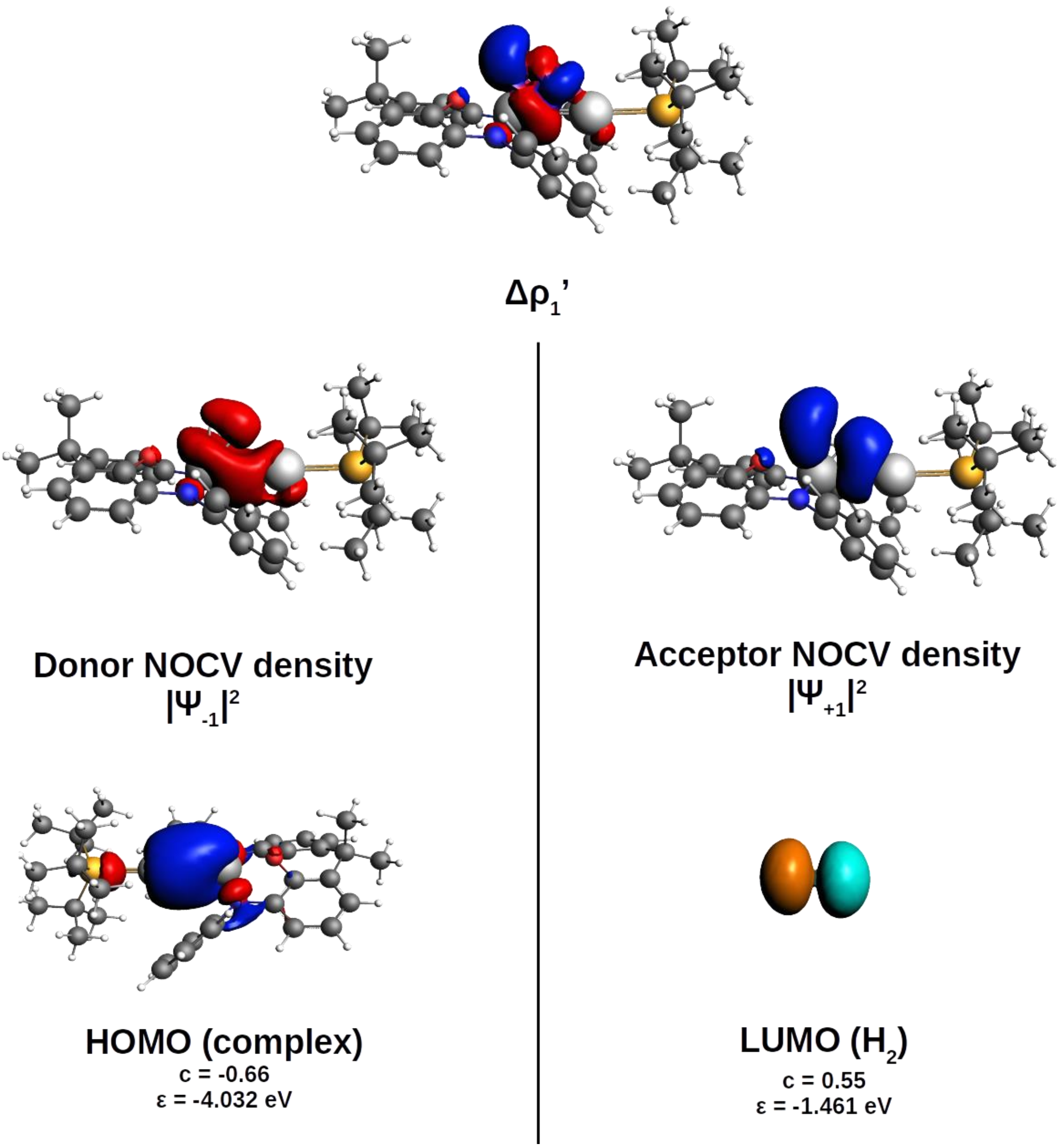

Figure S13. Breakdown of the donor ($|\Psi_1|^2$) and acceptor ($|\Psi_{-1}|^2$) NOCV densities that are associated with the deformation density $\Delta\rho'_1$ in the transition state **TS^{IV}_{Ag}** into the most important MOs of the fragments frozen at their **TS^{IV}_{Ag}** geometry. The molecular orbitals' mixing coefficients (c) are given together with their energy (ε).

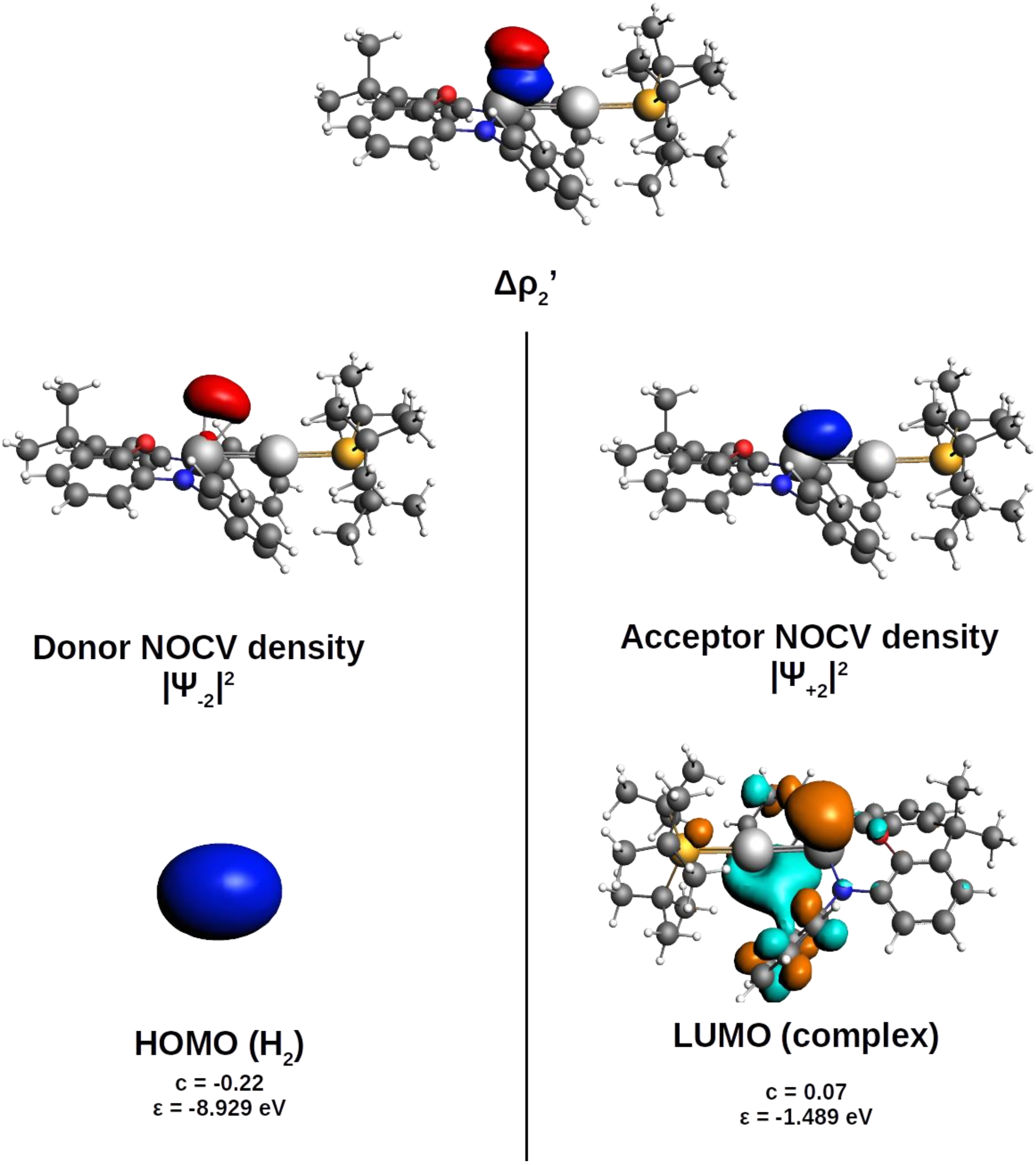

Figure S14. Breakdown of the donor ($|\Psi_2|^2$) and acceptor ($|\Psi_{-2}|^2$) NOCV densities that are associated with the deformation density $\Delta\rho'_2$ in the transition state $\mathbf{TS^{IV}_{Ag}}$ into the most important MOs of the fragments frozen at their $\mathbf{TS^{IV}_{Ag}}$ geometry. The molecular orbitals' mixing coefficients (c) are given together with their energy (ε).

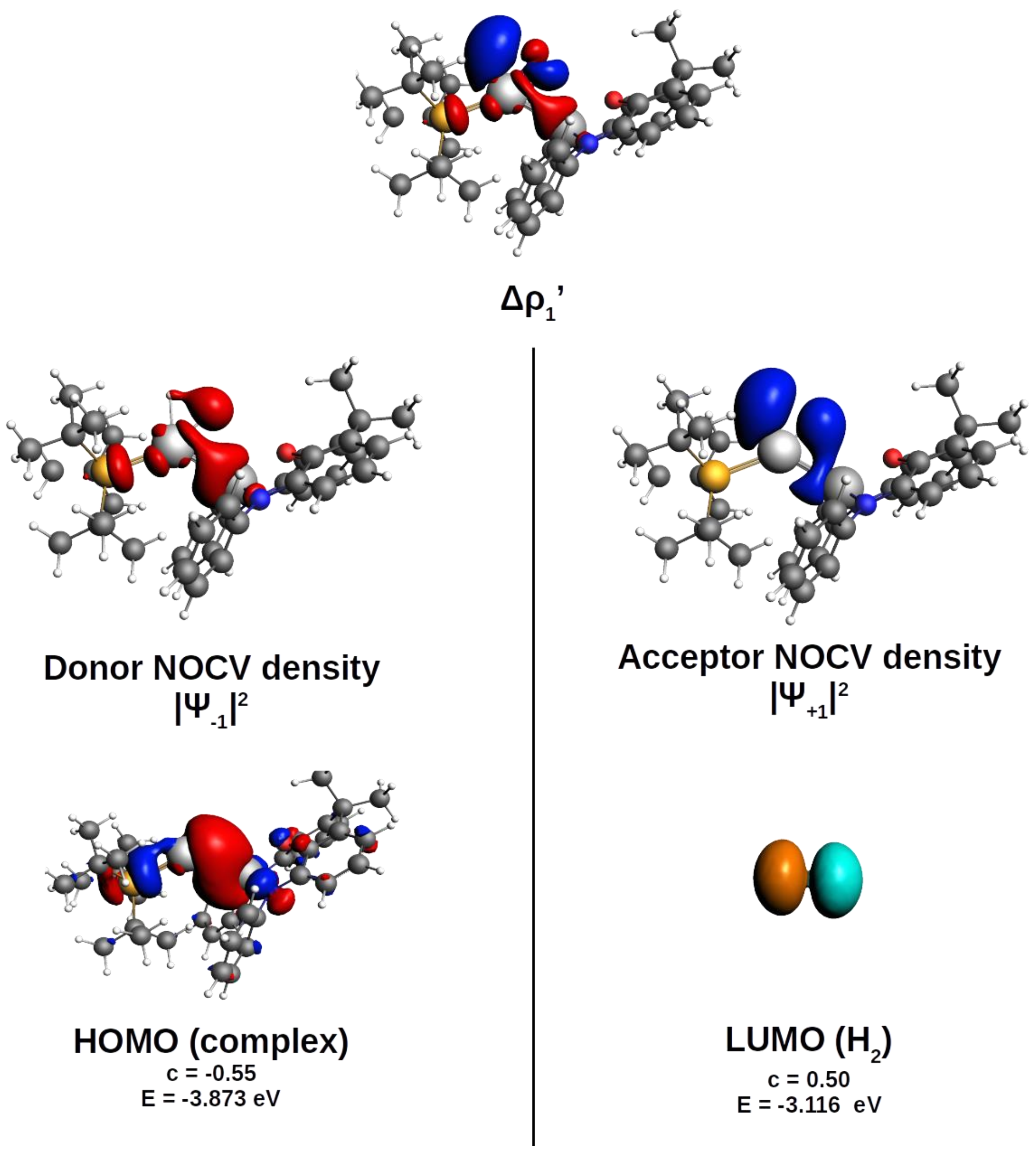

Figure S15. Breakdown of the donor ($|\Psi_1|^2$) and acceptor ($|\Psi_{-1}|^2$) NOCV densities that are associated with the deformation density $\Delta\rho'_1$ in the transition state $\mathbf{TS^{I}_{Ag}}$ into the most important MOs of the fragments frozen at their $\mathbf{TS^{I}_{Ag}}$ geometry. The molecular orbitals' mixing coefficients (c) are given together with their energy (ε).

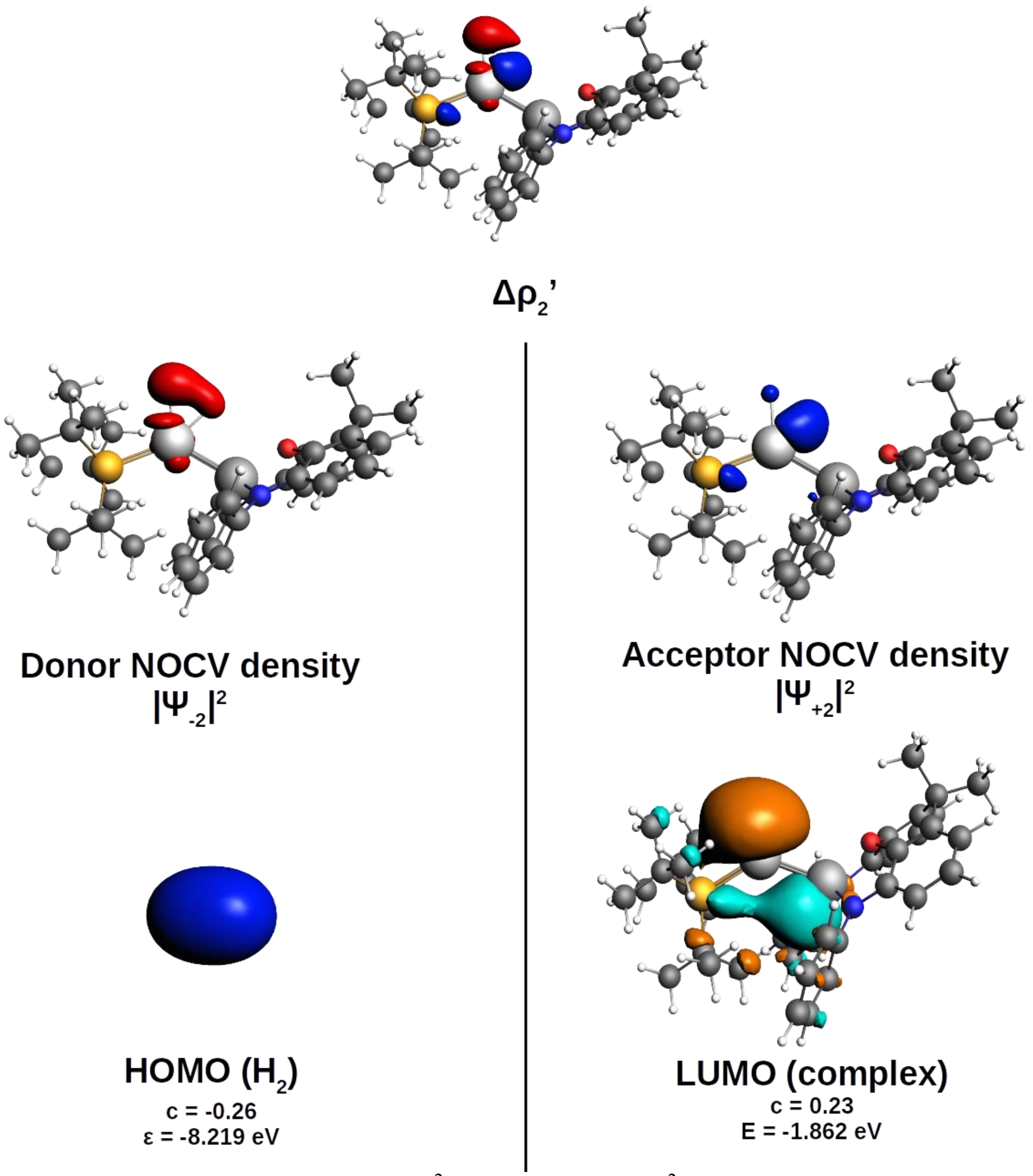

Figure S16. Breakdown of the donor ($|\Psi_2|^2$) and acceptor ($|\Psi_{-2}|^2$) NOCV densities that are associated with the deformation density $\Delta\rho'_2$ in the transition state $\mathbf{TS^{I}_{Ag}}$ into the most important MOs of the fragments frozen at their $\mathbf{TS^{I}_{Ag}}$ geometry. The molecular orbitals' mixing coefficients (c) are given together with their energy (ε).

M-Al bond analysis

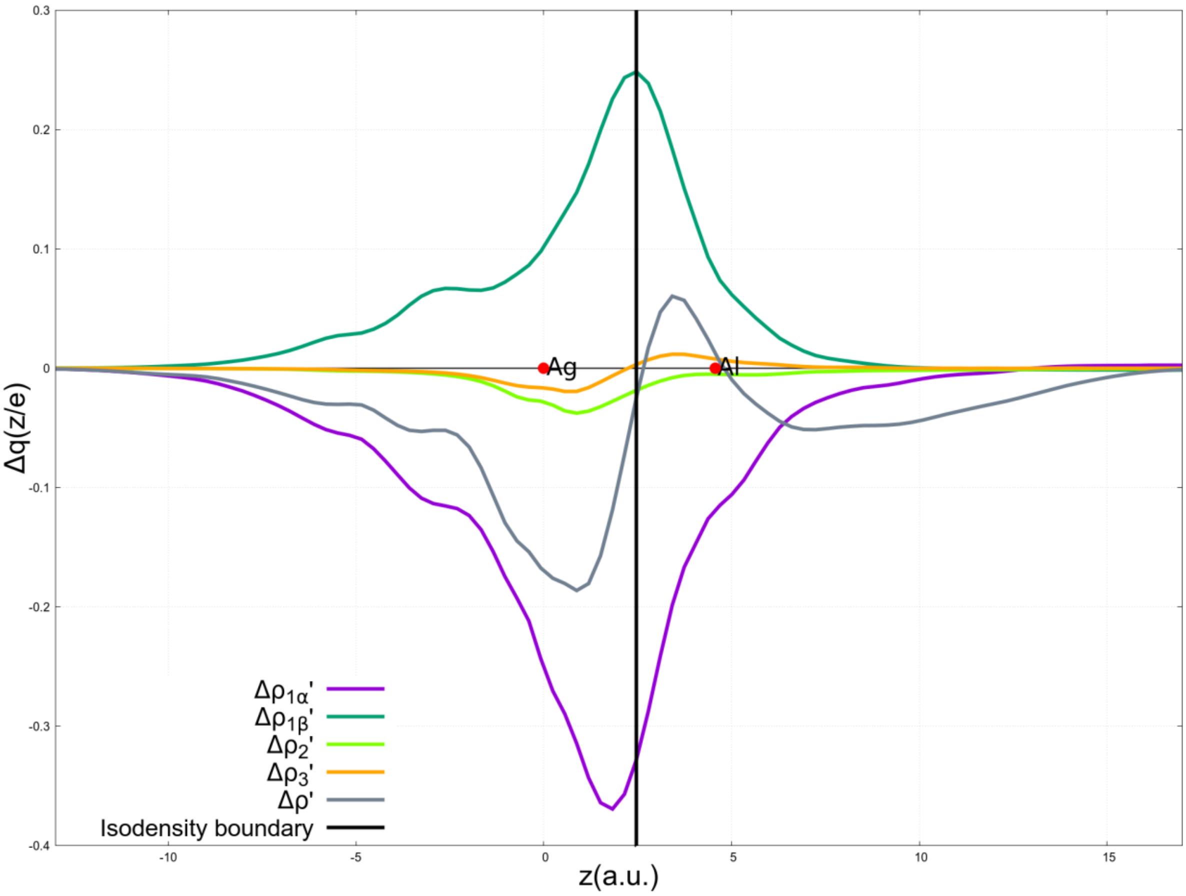

Figure S17. Charge Displacement (CD) curves for the interaction between open shell doublet [^{t}Bu$_3$PAg] and [Al(NON)] fragments in the [^{t}Bu$_3$PAgAl(NON)] complex. Red dots indicate the position of the main nuclei along the z axis. The vertical solid line marks the isodensity boundary between the fragments. Positive values of the curve indicate right-to-left charge flux and viceversa (see Methodology section for details).

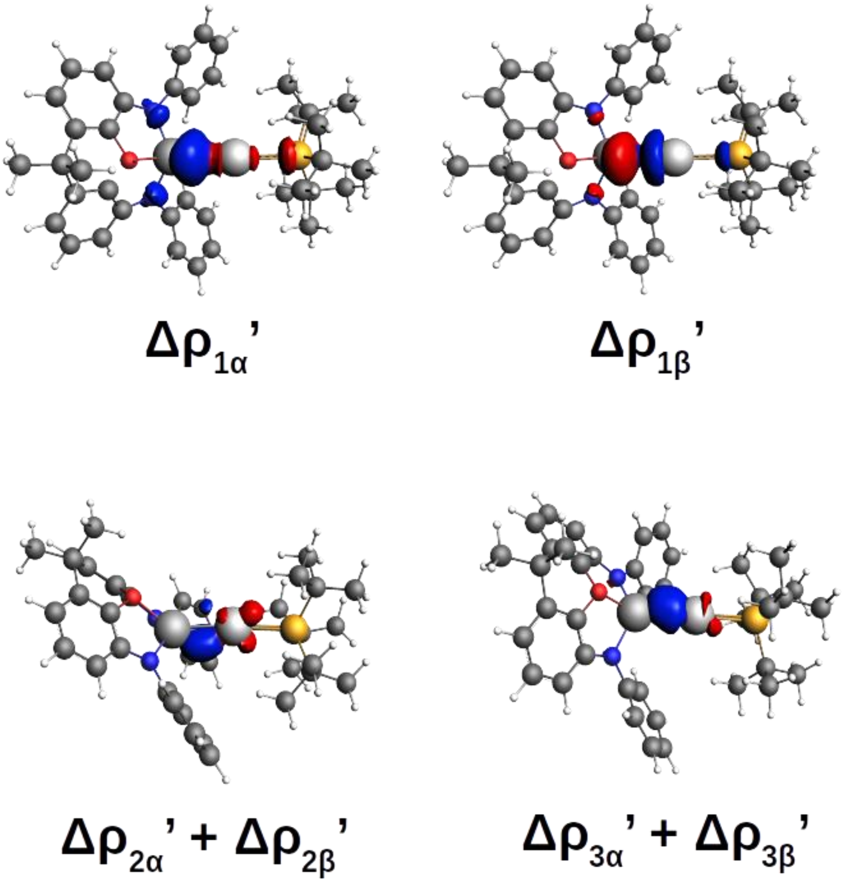

Figure S18. Isodensity surfaces of the main NOCV deformation densities for the interaction between open shell doublet [^{t}Bu$_3$PAg] and [Al(NON)] fragments in the [^{t}Bu$_3$PAgAl(NON)] complex. Blue regions indicate electron density accumulation areas, whereas red regions indicate depletion areas.

[^{t}Bu$_3$PCuAl(NON)]						
**Net CT (e)**	**-0.027**					
	**α**			**Β**		
**k**	**$\|\nu_k\|$**	**ΔE_{oi}^k (kcal/mol)**	**CT^k (e)**	**$\|\nu_k\|$**	**ΔE_{oi}^k (kcal/mol)**	**CT^k (e)**
**1**	0.50	-36.91	-0.324	0.32	-14.80	0.242
**2**	0.08	-1.22	-0.012	0.08	-1.35	-0.017
**3**	0.06	-1.19	-0.015	0.06	-0.92	-0.006
**[^{t}Bu$_3$PAgAl(NON)]**						
**Net CT (e)**	**-0.024**					
	**α**			**Β**		
**K**	**$\|\nu_k\|$**	**ΔE_{oi}^k (kcal/mol)**	**CT^k (e)**	**$\|\nu_k\|$**	**ΔE_{oi}^k (kcal/mol)**	**CT^k (e)**
**1**	0.52	-36.60	-0.327	0.33	-13.28	0.248
**2**	0.06	-1.20	-0.008	0.06	-1.37	-0.008
**3**	0.06	-1.01	0.009	0.05	-1.00	-0.006
**[^{t}Bu$_3$PAuAl(NON)]**						
**Net CT (e)**	**0.050**					
	**α**			**Β**		
**K**	**$\|\nu_k\|$**	**ΔE_{oi}^k (kcal/mol)**	**CT^k (e)**	**$\|\nu_k\|$**	**ΔE_{oi}^k (kcal/mol)**	**CT^k (e)**
**1**	0.45	-32.66	-0.272	0.42	-24.49	0.299
**2**	0.09	-2.03	-0.015	0.09	-2.32	-0.015
**3**	0.07	-1.59	-0.009	0.07	-1.67	-0.009

Table S3. Eigenvalues ($|\nu_k|$), orbital interaction energies ($\Delta E_{oi}{}^k$) and charge transfer (CT^k) associated with the first three NOCV deformation densities (k=1-3) and with the corresponding α and β components for the interaction between open shell doublet [^{t}Bu$_3$PM] and [Al(NON)] fragments in the [^{t}Bu$_3$PMAl(NON)] complexes (M=Cu, Ag, Au). Data for M=Cu, Au have been taken from Ref. [16]□.

The CD-NOCV and EDA approaches have been previously used in Ref. [16]□ to quantitatively assess the nature of the Au-Al and Cu-Al bonds in the corresponding [^{t}Bu$_3$PMAl(NON)] complexes (M=Cu, Au), revealing the presence of an electron-sharing bond with very similar features.

The same approach has been used here to analyse the Ag-Al bond in the [^{t}Bu$_3$PAgAl(NON)] complex. From a qualitative perspective, as shown in Figures S17-S18, the metal-aluminyl bond along the coinage-metal group is analogous, consisting of a metal-to-aluminyl σ charge transfer ($\Delta\rho_{1\alpha}$') and an opposite aluminyl-to-metal charge transfer ($\Delta\rho_{1\beta}$') of similar extent. In addition, two dative gold-to-aluminyl π back-donation components ($\Delta\rho_2$' and $\Delta\rho_3$') are found of much smaller extent.

The M-Al bond in these complexes is also analogous from a quantitative perspective, as can be envisaged from the comparison of the data reported in Table S2. Indeed, overall, all back-donation components feature a very small charge transfer (less than 0.02 e in absolute value in all cases), meaning that the contribution of these components to the overall bond is nearly negligible. Concerning the $\Delta\rho_{1\alpha}$' component, copper and silver feature slightly higher charge transfer in absolute value with respect to gold (-0.324, -0.327 and -0.272 e for Cu, Ag and Au, respectively). Conversely, Cu and Ag complexes feature slightly lower charge transfer values for the $\Delta\rho_{1\beta}$' component with respect to Au (0.242, 0.248 and 0.299 e for Cu, Ag and Au, respectively).

Analysis of the net charge transfer between metal and aluminyl fragments should give a quantitative measure of the bond polarization. In the case of gold, a slightly positive net charge transfer value (0.050 e) indicates a minimal Au(δ-)-Al(δ+) polarization, whereas both Cu and Ag complexes feature slightly negative charge transfer values (-0.024 and -0.027 e, respectively), indicating a very small polarization as M(δ+)-Al(δ-). Overall, however, these net charge transfer values are very small in absolute value, approaching very closely the zero (which is the value of a perfectly apolar electron-sharing bond), meaning that the M-Al bond in the corresponding [^{t}Bu$_3$PMAl(NON)] complexes is a poorly polarized electron-sharing bond with analogous features.

Frontier Molecular Orbitals (FMO) analysis

[^{t}Bu$_3$PCuAl(NON)]		[^{t}Bu$_3$PAgAl(NON)]		[^{t}Bu$_3$PAuAl(NON)]	
**TS^I**	**TS^{IV}**	**TS^I**	**TS^{IV}**	**TS^I**	**TS^{IV}**
**LUMO Energy (eV)**					
-1.880	-1.543	-1.862	-1.489	-2.101	-1.552
**Atomic orbital contributions**					
Cu 4p$_z$ 23.7%	Al 3p$_z$ 23.5 %	Ag 5s 27.7%	Al 3p$_z$ 22.5 %	Au 6s 25.9%	Al 3p$_z$ 30.9%
Cu 4s 20.2 %	Cu 4p$_z$ 14.2%	Ag 5p$_z$ 20.3%	Ag 5p$_z$ 9.9%	Al 3p$_z$ 19.5%	Au 6p$_z$ 7.7 %
Al 3p$_z$ 19.9 %	Cu 4s 3.5 %	Al 3p$_z$ 17.0 %	Ag 5s 4.7 %	Au 6p$_z$ 17.3 %	Al 3s 3.8%
Al 3s 6.0%	/	Al 3s 8.6%	/	Al 3s 14.5%	/
**Tot^{Al} %:** 25.9 %	**Tot^{Al} %:** 23.5 %	**Tot^{Al} %:** 25.6 %	**Tot^{Al} %:** 22.5 %	**Tot^{Al} %:** 34.0 %	**Tot^{Al} %:** 34.2 %
**Tot^{Cu} %:** 43.9 %	**Tot^{Cu} %:** 17.7 %	**Tot^{Ag} %:** 48.0 %	**Tot^{Ag} %:** 14.6 %	**Tot^{Au} %:** 43.2 %	**Tot^{Au} %:** 7.7 %

Table S4. Frontier Molecular Orbitals (FMO) comparative analysis of the LUMO of the [^{t}Bu$_3$PMAl(NON)] (M=Cu, Ag, Al) complexes at the corresponding **TS^{IV}$_M$** and **TS^I$_M$** structures.

Activation Strain Model (ASM) analysis

	RC_{Cu}	TS^{IV}_{Cu}	PC_{Cu}	TS^{I}_{Cu}	INT_{Cu}	TS^{II}_{Cu}	PC'_{Cu}
ΔE	-2.6	22.4	-18.5	7.3	0.9	3.8	-24.5
ΔE_{int}	-2.5	-13.1	-187.2	-47.5	-132.8	-155.6	-155.5
ΔE_{dist}^{H2}	0.0	30.3	132.5	49.7	115.4	129.3	127.3
ΔE_{dist}^{compl}	0.0	5.2	36.2	5.2	18.3	30.1	3.7
ΔE_{dist}	0.0	3.5	168.7	54.8	133.7	159.4	131.0

Table S5. Results of the Activation Strain Model (ASM) analysis of the $[H_2]$-$[^tBu_3PCuAl(NON)]$ interaction for stationary points **RC_{Cu}**, **TS^{IV}_{Cu}**, **PC_{Cu}**, **TS^{I}_{Cu}**, **INT_{Cu}** , **TS^{II}_{Cu}** and **PC'_{Cu}**. All energies are expressed in kcal/mol.

	RC_{Ag}	TS^{IV}_{Ag}	PC_{Ag}	TS^{I}_{Ag}	INT_{Ag}	TS^{II}_{Ag}	PC'_{Ag}
ΔE	-2.2	25.5	-13.4	24.5	13.1	14.2	-12.2
ΔE_{int}	-2.2	-10.0	-183.1	-48.6	-136.9	-148.3	-147.2
ΔE_{dist}^{H2}	0.0	29.3	133.5	61.7	130.8	134.8	130.3
ΔE_{dist}^{compl}	0.0	6.3	36.2	11.4	19.2	27.8	4.8
ΔE_{dist}	0.1	35.6	169.7	73.1	150.0	162.5	135.0

Table S6. Results of the Activation Strain Model (ASM) analysis of the $[H_2]$-$[^tBu_3PAgAl(NON)]$ interaction for stationary points **RC_{Ag}, TS^{IV}_{Ag}, PC_{Ag}, TS^{I}_{Ag}, INT_{Ag}, TS^{II}_{Ag} and PC'_{Ag}**. All energies are expressed in kcal/mol.

	RC_{Au}	TS^{IV}_{Au}	PC_{Au}	TS^{I}_{Au}	INT_{Au}	TS^{II}_{Au}	PC'_{Au}	TS^{III}_{Au}
ΔE	-2.0	24.7	-7.6	20.0	9.2	19.8	-4.6	-1.5
ΔE_{int}	-2.4	-13.1	-181.1	-40.5	-137.7	-149.2	-165.3	-138.7
ΔE_{dist}^{H2}	0.0	29.3	133.1	41.8	122.1	129.4	138.4	129.8
ΔE_{dist}^{compl}	0.4	8.5	40.5	18.7	24.9	39.6	22.3	7.4
ΔE_{dist}	0.4	37.8	173.6	60.5	146.9	169.0	160.7	137.2

Table S7. Results of the Activation Strain Model (ASM) analysis of the $[H_2]$-$[^tBu_3PAuAl(NON)]$ interaction for stationary points **RC_{Au}**, **TS^{IV}_{Au}**, **PC_{Au}**, **TS^{I}_{Au}**, **INT_{Au}**, **TS^{II}_{Au}**, **PC'_{Au}**, and **TS^{III}_{Au}**. All energies are expressed in kcal/mol.

The application of the Activation Strain Model helps highlighting some of the differences occurring between the two reaction paths described in the main text. As shown in Tables S4-S6, the difference between **TS^{I}** and **TS^{IV}** for all three complexes is also evident from the electronic energy activation barrier ΔE, with the values associated with **TS$^{I}_{Cu}$ / TS$^{I}_{Ag}$ / TS$^{I}_{Au}$** (7.3, 24.5 and 20.0 kcal/mol, respectively) being lower than those associated to **TS$^{IV}_{Cu}$ / TS$^{IV}_{Ag}$ / TS$^{IV}_{Au}$** (22.4, 25.5 and 24.7 kcal/mol, respectively). The lower barrier associated to **TS$^{I}_{M}$** arises from a more favourable interaction energy stabilization ΔE_{int} at **TS$^{I}_{Cu}$ / TS$^{I}_{Ag}$ / TS$^{I}_{Au}$** (-47.5, -48.6, -40.5 kcal/mol, respectively) with respect to that at **TS$^{IV}_{Cu}$ / TS$^{IV}_{Ag}$ / TS$^{IV}_{Au}$** (-13.1, -10.0, -13.1 kcal/mol, respectively), which is consistent with the enhanced H_2-complex interaction for **TS$^{I}_{M}$** described in the main text. Thus, although the distortion penalty ΔE_{dist} is larger for **TS$^{I}_{M}$**, the interaction energy is able to counterbalance it more efficiently for **TS$^{I}_{M}$**, resulting in a lower activation barrier in all cases. An additional information that can be gathered from the ASM diagrams concerns the stability of **INT$_{M}$** compared to **PC$_{M}$**. Indeed, in both structures the H-H bond dissociation leads to an overall high distortion penalty ΔE_{dist} (for instance, 146.9 vs. 173.6 kcal/mol for **PC$_{Au}$** and **INT$_{Au}$,** respectively), which, however, is efficiently counterbalanced by a stabilizing interaction energy ΔE_{int} only in the case of **PC$_{M}$** (for instance, -181.1 vs. -137.7 kcal/mol for **PC$_{Au}$** and **INT$_{Au}$,** respectively), resulting in a stable **PC$_{M}$** (overall ΔE for **PC$_{Au}$** is -7.6 kcal/mol) and an unstable **INT$_{M}$** (ΔE=9.2 kcal/mol for **INT$_{Au}$**). This finding reflects, as also discussed in the main text, the atypical behaviour of coinage metal-aluminyl complexes in the framework of bimetallic compounds reacting with H_2, with the related oxidative addition product being unstable because of unfavourable interactions with H_2.

$\alpha_{P\text{-}M\text{-}Al}$	$^tBu_3PCuAl(NON)$		$^tBu_3PAgAl(NON)$		$^tBu_3PAuAl(NON)$	
	% $4p_z$	% 4s	% $5p_z$	% 5s	% $6p_z$	% 6s
160º	17.8	5.6	14.6	7.1	13.2	5.8
150º	18.5	9.4	15.3	11.9	14.3	10.2
140º	19.5	14.0	16.0	17.7	15.7	15.3
130º	21.3	19.4	17.4	24.1	17.1	20.9
120º	25.6	21.1	17.9	31.2	18.2	25.9

Table S8. Contribution of copper, silver and gold ns and np_z atomic orbitals (n=4,5,6, respectively) to the LUMO of the corresponding aluminyl complex optimized with constrained fixed P-M-Al (M = Cu, Ag, Au) angles (in degrees).

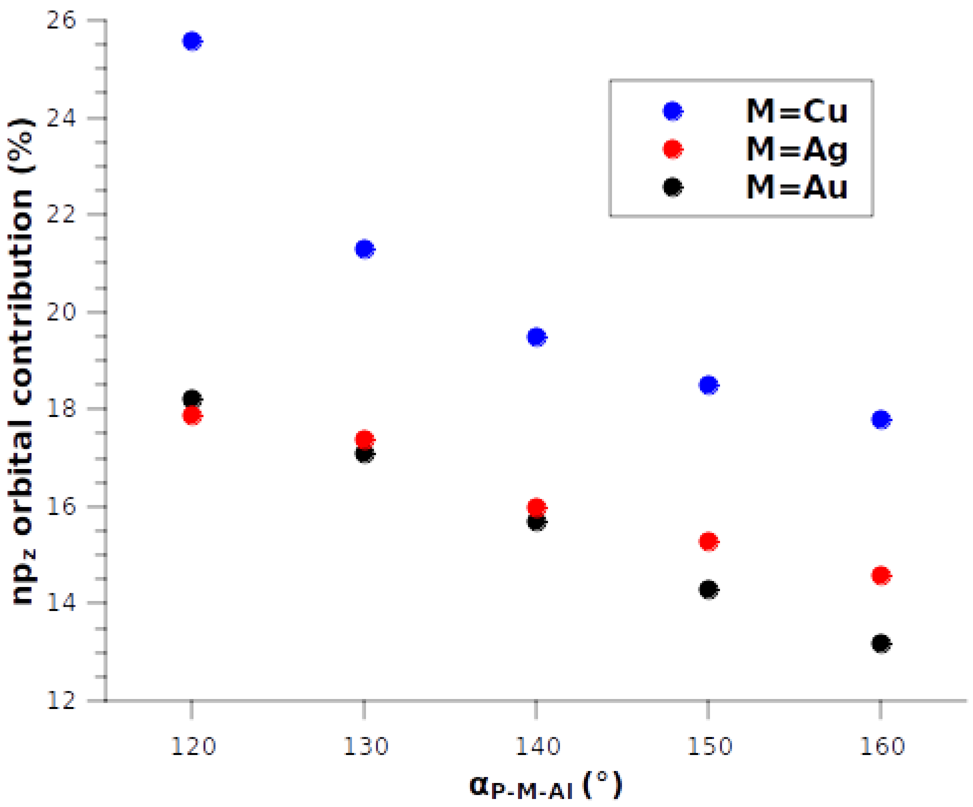

Figure S19 Contribution of copper, silver and gold np_z atomic orbitals (n=4,5,6, respectively) to the LUMO of the corresponding aluminyl complex optimized with constrained fixed P-M-Al (M = Cu, Ag, Au) angles.

Comparison with literature results: cooperative H_2 activation with a bimetallic Pt-Al complex

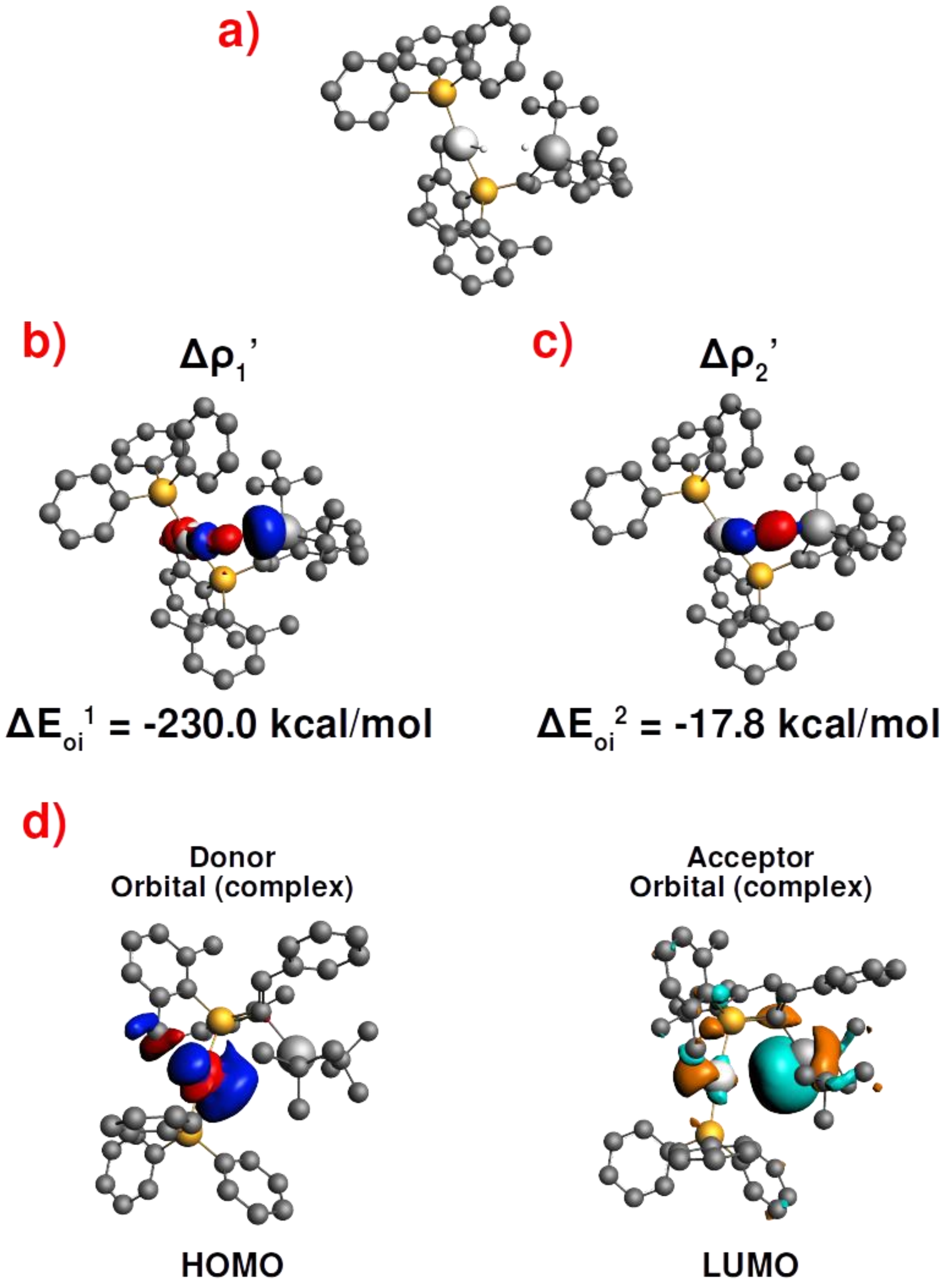

Figure S20. Results of the ETS-NOCV analysis of the $[H_2]$-[(L)AlPt(PPh_3)(ethylene)] (L=[Mes_2PC(=CHPh)^tBu_2]) interaction occuring at the second transition state (TS2) for the oxidative addition of H_2 to the [(L)AlPt(PPh_3)(ethylene)] complex reported in ref. [17] The structure of the transition state is shown (*a*) together with the isosurfaces for the $\Delta\rho_1$' and $\Delta\rho_2$' (*b*) and *c*), respectively). The main donor and acceptor molecular orbitals of the complex contributing to each are also shown (*d*). Isovalue is 5 me/a_0^3 for $\Delta\rho_1$', 2 me/a_0^3 for $\Delta\rho_2$' and 30 me/a_0^3 for the MO isosurfaces.

$[H_2]$-[$^tBu_3PAuAl(NON)$] *(HOMO complex at TS^{IV})*	$[H_2]$-[$^tBu_3PAuAl(NON)$] *(HOMO complex at TS^{I})*	$[H_2]$-[(L)AlPt(PPh_3)(ethylene)] *(HOMO complex at TS2)*
**Energy (eV)**		
-4.38	-3.89	-3.99
**Atomic orbital contributions**		
Al 3s (11.6%)	Al 3s (13.1%)	Pt $5d_{z2}$ (43.6%)
Au 6s (10.1%)	Au $6p_x$ (9.8%)	Pt 6s (17.7%)
Al $3p_x$ (7.9%)	Au 6s (8.1%)	Pt $5d_{yz}$ (8.8%)
Au $6p_x$ (6.0%)	Au $5d_{xz}$ (8.2%)	Al $3p_x$ (3.0%)
/	Al $3p_x$ (4.8%)	Pt $5d_{x2-y2}$ (2.1%)
Total Al contributions: 19.5%	Total Al contributions: 17.9%	Total Al contributions: 3.0 %
Total Au contributions: 16.1%	Total Au contributions: 26.1%	Total Pt contributions: 72.2%

Table S9. Energy and atomic orbital contributions to the main donor molecular orbitals at the transition state $\mathbf{TS^I}$ for the H_2 activation with the [$^tBu_3PAuAl(NON)$] complex (left). The results are compared with those obtained by the same analysis carried out at the second transition state (TS2) for the H_2 activation with the [(L)AlPt(PPh_3)(ethylene)] complex (right) (L=[$Mes_2PC(=CHPh)^tBu_2$]) reported in ref. [17]

The reactivity of the aluminium-platinum complex [(L)AlPt(PPh_3)(ethylene)] (L=[$Mes_2PC(=CHPh)^tBu_2$]) with H_2, resulting in the oxidative addition of dihydrogen to the Pt(0) center, has been investigated both experimentally and theoretically by Bourissou and coworkers and represents one of the first H_2 activation processes involving a late transition metal (Pt) and a main-group Lewis acid (Al).[17] In the paper, the authors clearly state that the reaction is assisted by the electrophilic behavior of Al and that the Pt-Al bond in the complex is of dative (Pt→ Al) nature.

The authors report a two step reaction mechanism. In the first step (via the transition state TS1) an intermediate in which the H_2 molecule is trapped between Al and Pt is formed and subsequently, via transition state TS2, the H-H bond undergoes breaking and the addition product is formed, where one hydrogen is bridged between Al and Pt and the other is bound to Pt (similarly to **PC**).

Since TS2 leads to the formation of a product which resembles the product **PC** we discuss in

this work, it represents an ideal test case for the application of the NOCV methodology to investigate any analogy/difference with the **TS^{I}** and **TS^{IV}** we discuss here.

The results of the NOCV analysis at TS2 (Figure S20) depicts an interaction between H_2 and the [(L)AlPt(PPh_3)(ethylene)] complex which is apparently of similar nature to that discussed for **TS^{I}** and **TS^{IV}** . Indeed, we find a dominant component ($\Delta\rho_1$') which represents the population of the LUMO of H_2 from the HOMO of the complex and a – smaller in extent – inverse charge flux ($\Delta\rho_2$') describing the charge transfer from the H_2 HOMO towards the Al-centered LUMO of the complex. While the analogy is consistent for the $\Delta\rho_2$' component (particularly for **TS^{IV}**, since it features the Lewis acid behavior of Al), qualitative discrepancies emerge for the dominant component. Indeed, while, as discussed in the main text, for both **TS^{I}** and **TS^{IV}**, the HOMO (main donor MO for the $\Delta\rho_1$' component) is found to be almost equally localized on both Au and Al atoms, the HOMO of the [(L)AlPt(PPh_3)(ethylene)] complex (main donor MO as well) is centered practically only on Pt. This can be assessed quantitatively by analyzing the atomic orbital contributions to these MOs (Table S8). For **TS^{IV}**, we find almost equal contribution from Al and Au (19.5% and 15.9%, respectively), consistently with an electron-sharing weakly polarized bond in which both Au and Al have an important role in populating the H_2 LUMO. For **TS^{I}**, while we observe an increased contribution from Au (26.1%), the participation of Al is left practically unchanged (17.9%), consistently, yet again, with the Au-Al covalency being the driving force of this interaction. Conversely, and in agreement with the previous results,[17] for the [(L)AlPt(PPh_3)(ethylene)] complex, the molecular orbital is almost uniquely localized on Pt (72.2%) with negligible contributions from Al (3.0%). This is consistent with the picture of a dative Pt → Al bond, a Pt(δ-)-Al(δ+) polarization and a consequent oxidative addition occuring at the Pt site with the electrophilic assistance of Al, which is consistently different with respect to the mechanism we discuss for the coinage metal-Al bond.

Isomerization of the hydrogenated products: [RGeGeR] vs. [^{t}Bu$_3$PAuAl(NON)]

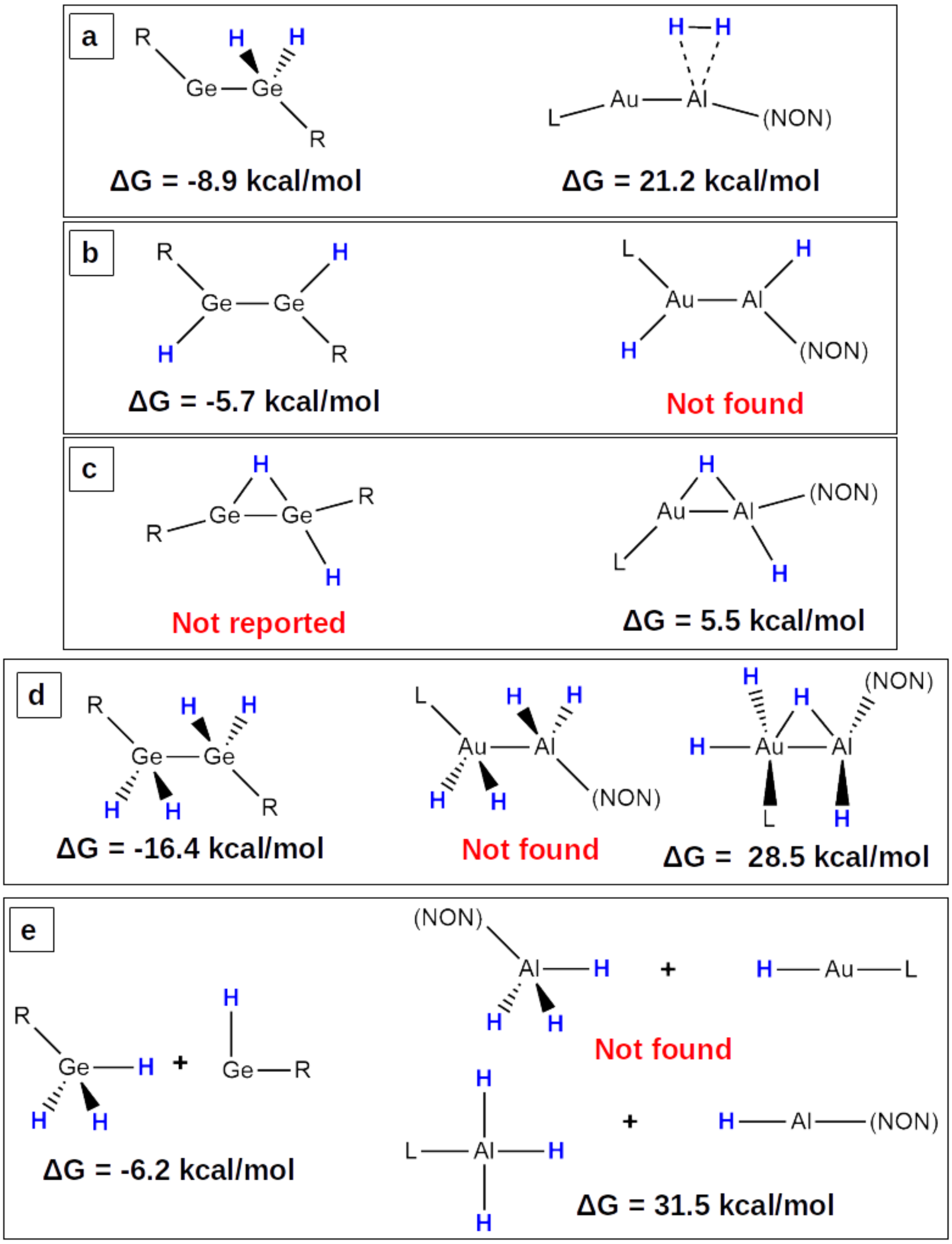

Scheme S1. Study of the evolution of singly-bridged products [RGe(μ-H)Ge(H)R] and [LAu(μ-H)Al(H)(NON)] (L=^{t}Bu$_3$P) to singly-hydrogenated and doubly-hydrogenated isomers. The Gibbs' free energy with respect to separated reactants [RGeGeR]/[LAuAl(NON)] and H_2 are reported for the cases where optimization procedures ended successfully. The results for digermyne compounds are taken from Ref. [18]

As shown in Scheme S1, the computational analysis carried out in this work excludes the thermodynamically favourable formation of singly-bridged isomers analogous to digermyne compounds in the case of the gold-aluminyl complex under study. In detail, in ref. [18] it has been extensively discussed that two main isomerization product come from single hydrogenation of digermyne (and distannyne) compounds, i.e. the mixed-valence asymmetrically hydrogenated [RGeGe(H_2)R] (Scheme S1a) and the symmetrically hydrogenated [R(H)GeGe(H)R] (Scheme S1b) compounds, which can be eventually experimentally detected upon tuning of the R steric hindrance. In our case, optimization of the equivalent [L(H)AuAl(H)(NON)] symmetrically hydrogenated isomer fails at each attempt, always leading to the "parent" singly-bridged molecule. Instead, optimization of the asymmetrically hydrogenated [LAuAl(H_2)(NON)] compound yields a thermodynamically unstable species (21.2 kcal/mol) (Scheme S1a). The only singly-hydrogenated isomer found to be reasonably stable is the *trans*-[LAu(μ-H)Al(H)(NON)] **PC'**, which, however, was not predicted for digermyne compounds (Scheme S1c).

Geometry optimizations show no thermodynamically feasible way of obtaining doubly-hydrogenated products in the case of [LAu(μ-H)Al(H)(NON)]. In detail, the analogous of [R(H_2)GeGe(H_2)R], which is found to be very stable for digermynes (-16.4 kcal/mol, Scheme S1d), is not found upon optimization for gold-aluminyl complex. Each attempt leads to the formation of an asymmetrically doubly-hydrogenated bridged structure [L(H_2)Au(μ-H)Al(H)(NON)], which is, however, found to be thermodynamically unstable (28.5 kcal/mol).

Finally, we attempted to optimize the hydrogenation products involving Au-Al bond breaking, which were found to be stable in the case of digermynes (-6.2 kcal/mol, Scheme S1e). For gold-aluminyl complex, the Au-Al bond breaking, as expected, does not pave thermodynamically favourable paths. When the dissociation features a gold monohydride [LAuH], the optimization of the corresponding aluminyl tri-hydride [(H_3)Al(NON)] failed at every attempt. In the other case, featuring the aluminium monohydride [HAl(NON)] and the tetra-coordinate gold complex [LAu(H_3)], optimizations were successful, but these products are found to be highly thermodynamically unstable (31.5 kcal/mol).

The same structural investigation has been carried out for both copper and silver complexes. Concerning singly-hydrogenated structures, attempts to optimize structures **a**-**c** lead unequivocally to doubly-bridged **PC'$_{Cu}$** and **PC'$_{Ag}$** for copper and silver complexes, respectively and, similarly, optimization of doubly-hydrogenated structure **d** leads to **PC'$_{Cu}$** and **PC'$_{Ag}$** with extrusion of H_2. In the case of the [^{t}Bu$_3$PCuAl(NON)], attempts to optimize mono- and trihydride complexes lead eventually to the Cu-Al bond breaking and formation of monohydride [^{t}Bu$_3$PCuH] and [HAl(NON)] species with H_2 extrusion, but this path is found to be thermodynamically not accessible. Conversely,

the same attempts for the silver complex lead to the tri-hydride silver species [$^tBu_3PAgH_3$] (analogous to structure **e** for Au in Scheme 1); the reaction, however, is found to be highly endergonic (ΔG=71.9 kcal/mol).

In light of this structural analysis, it is reasonable to conclude that the hydrogenated products for copper, silver and gold-aluminyl complexes are not expected to easily undergo isomerization at variance with the digermyne equivalent.

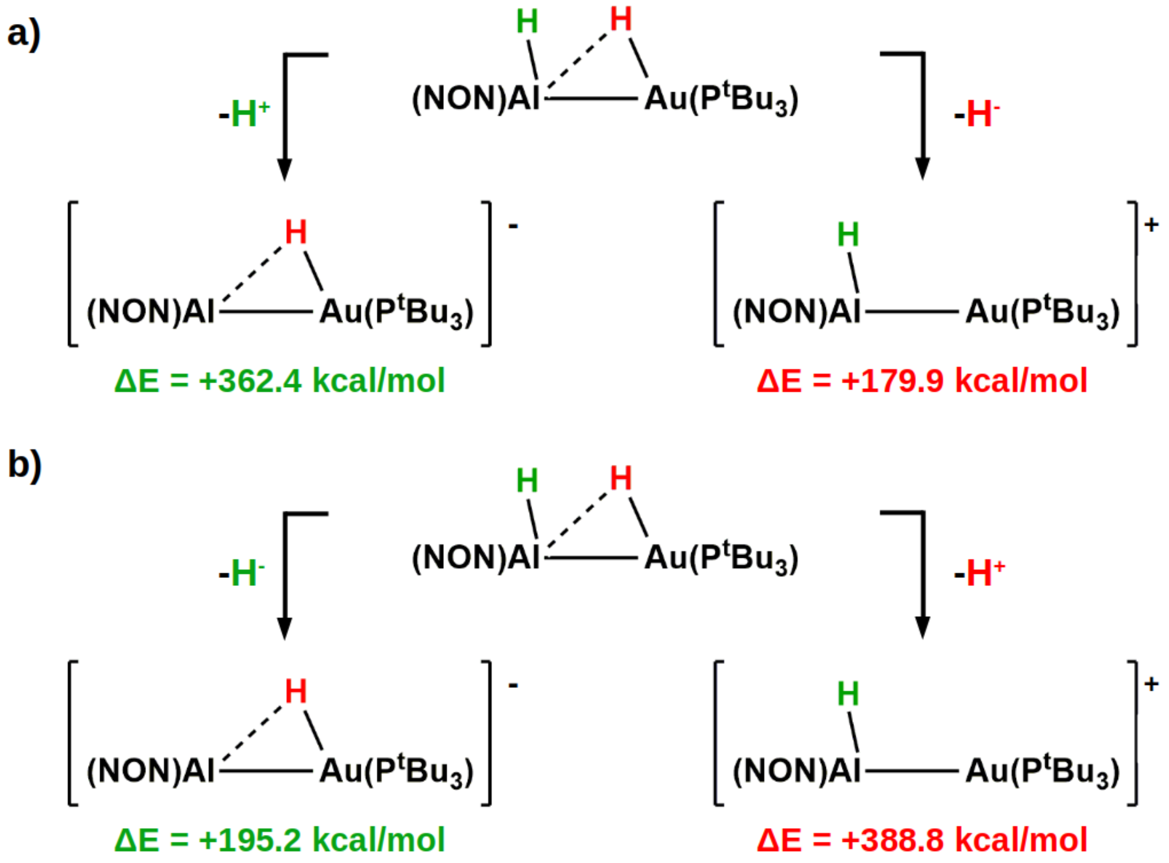

Figure S21. Pathways for the heterolytic breaking of the two H-substrate bonds on the singly-bridged product [^{t}Bu$_3$PAu(μ-H)AlH(NON)] (**PC$_{Au}$**) and corresponding associated energies.

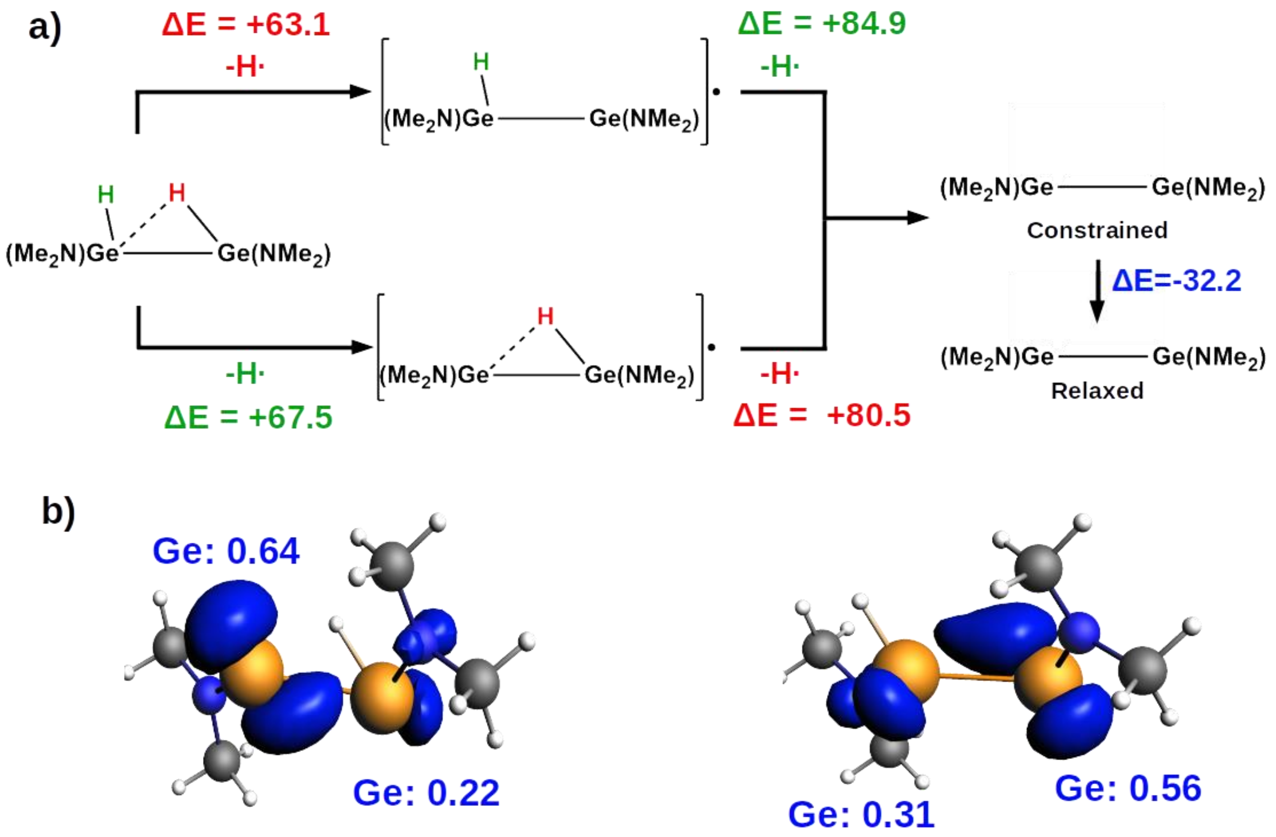

Figure S22. ***a)*** Pathways for the homolitic breaking of the H-substrate bonds on the singly-bridged product [(Me_2N)Ge(μ-H)GeH(NMe_2)] (**IM2Ge'** from Ref. [18]) and relative associated energies (expressed in kcal/mol). ***b)*** Spin density (in blue) associated with the [(Me_2N)Ge(μ-H)Ge(NMe_2)]· (left) and [(Me_2N)GeGeH(NMe_2)]· (right) radicals. The isovalue for the surface is 5 me/a_0^3. The most relevant atomic spin polarization values are reported.

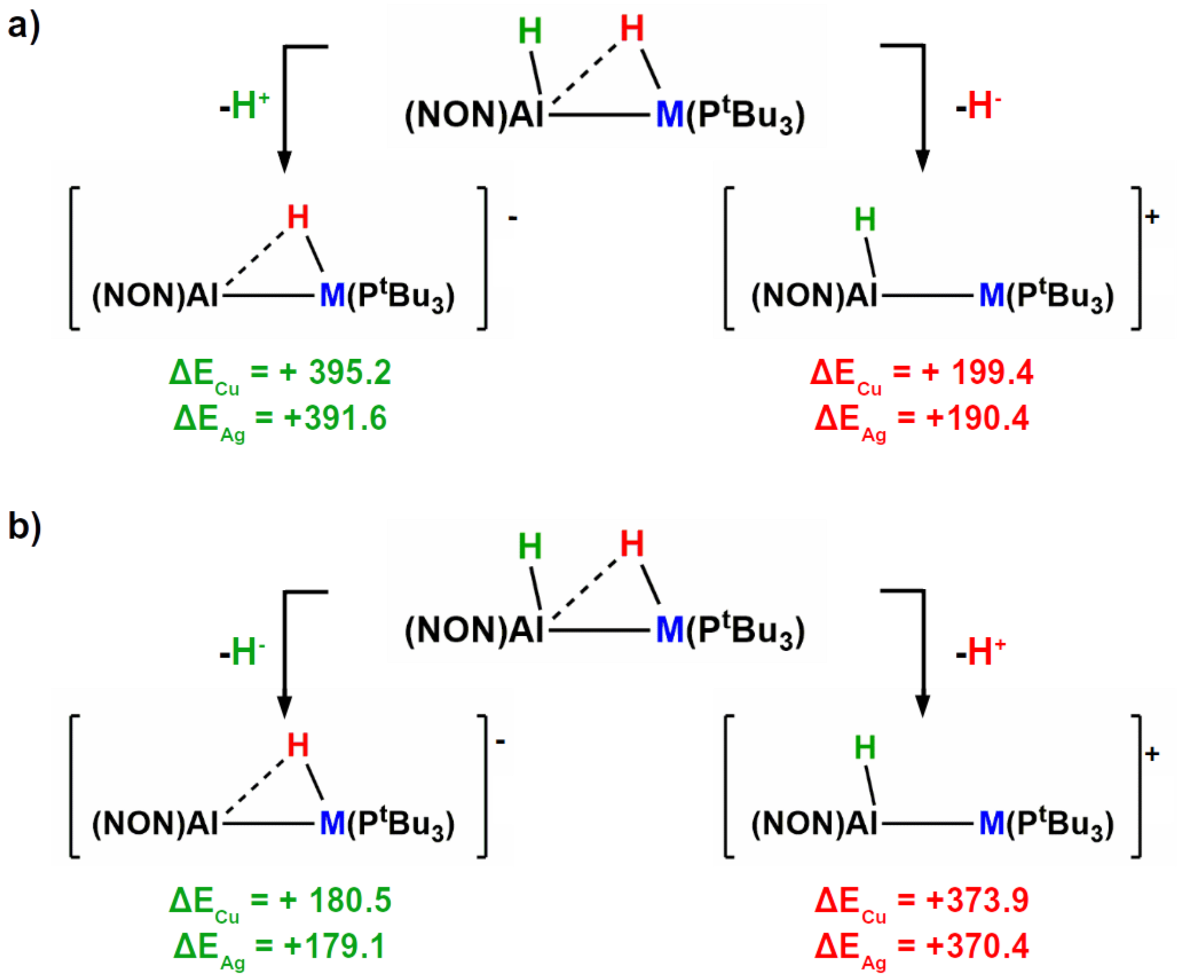

Figure S23. Pathways for the heterolytic breaking of the H-substrate bonds on the singly-bridged product [$^{t}Bu_3PM(\mu$-H)AlH(NON)] (M=Cu,Ag, $\mathbf{PC_{Cu}}$, $\mathbf{PC_{Ag}}$) and associated relative energies in kcal/mol.

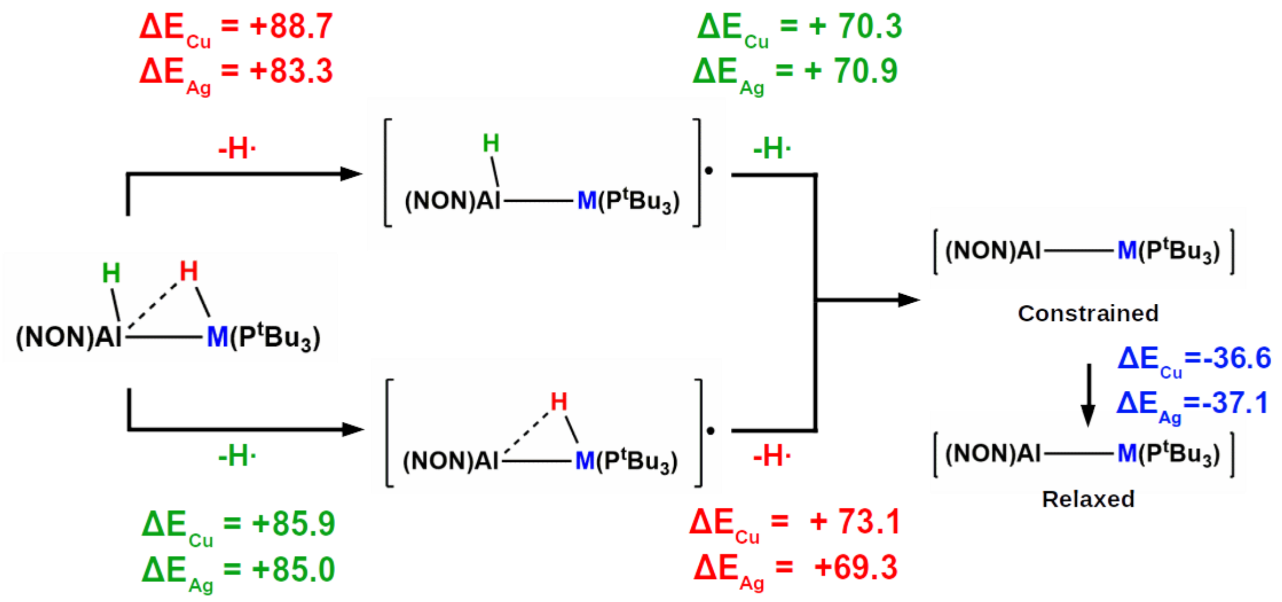

Figure S24. Pathways for the homolitic breaking of the H-substrate bonds on the singly-bridged product [^{t}Bu$_3$PM(μ-H)AlH(NON)] (M=Cu,Ag, **PC$_{Cu}$** , **PC$_{Ag}$**) and associated relative energies in kcal/mol.

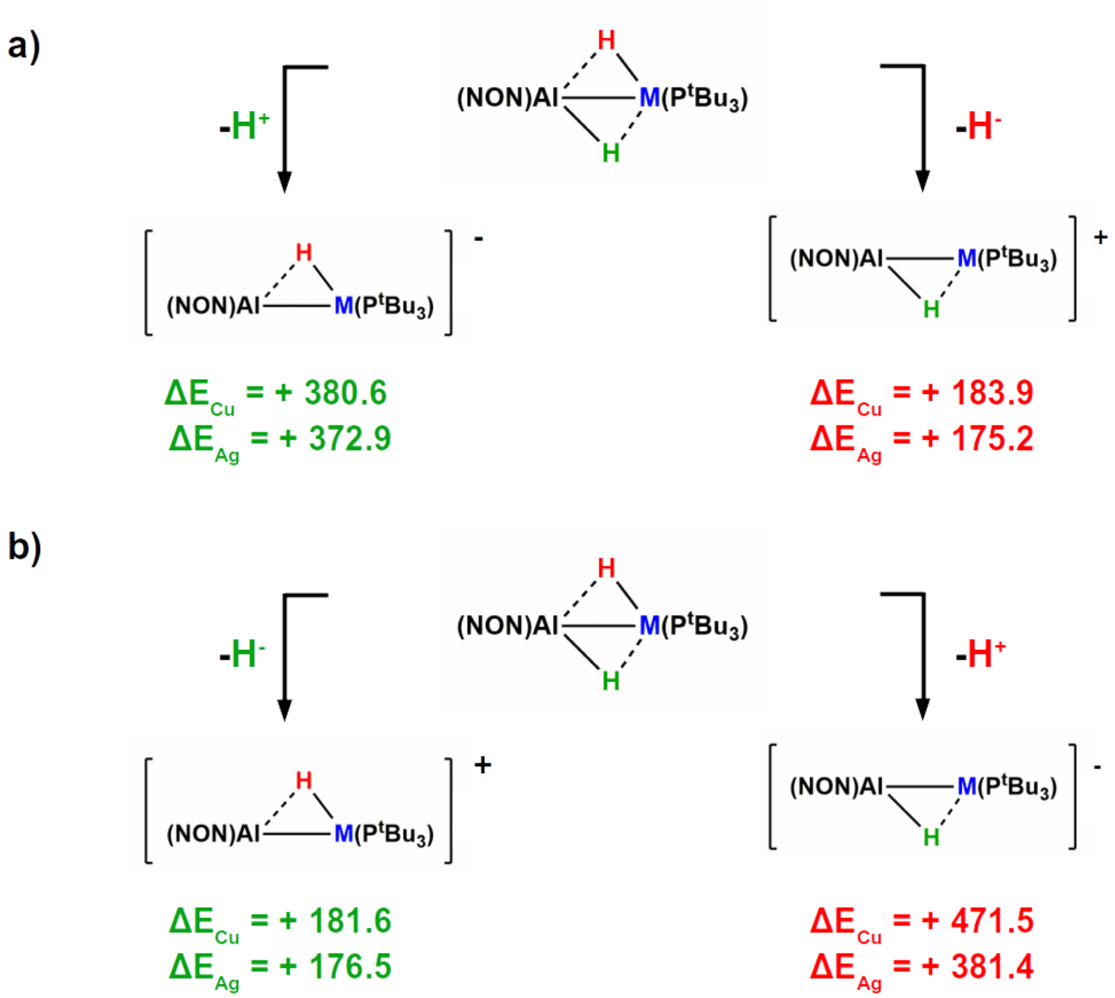

Figure S25. Pathways for the heterolytic breaking of the H-substrate bonds on the doubly-bridged product [^{t}Bu$_3$PM(μ-H)Al(μ-H)(NON)] (M=Cu,Ag, **PC$_{Cu}$'**, **PC$_{Ag}$'**) and associated relative energies in kcal/mol.

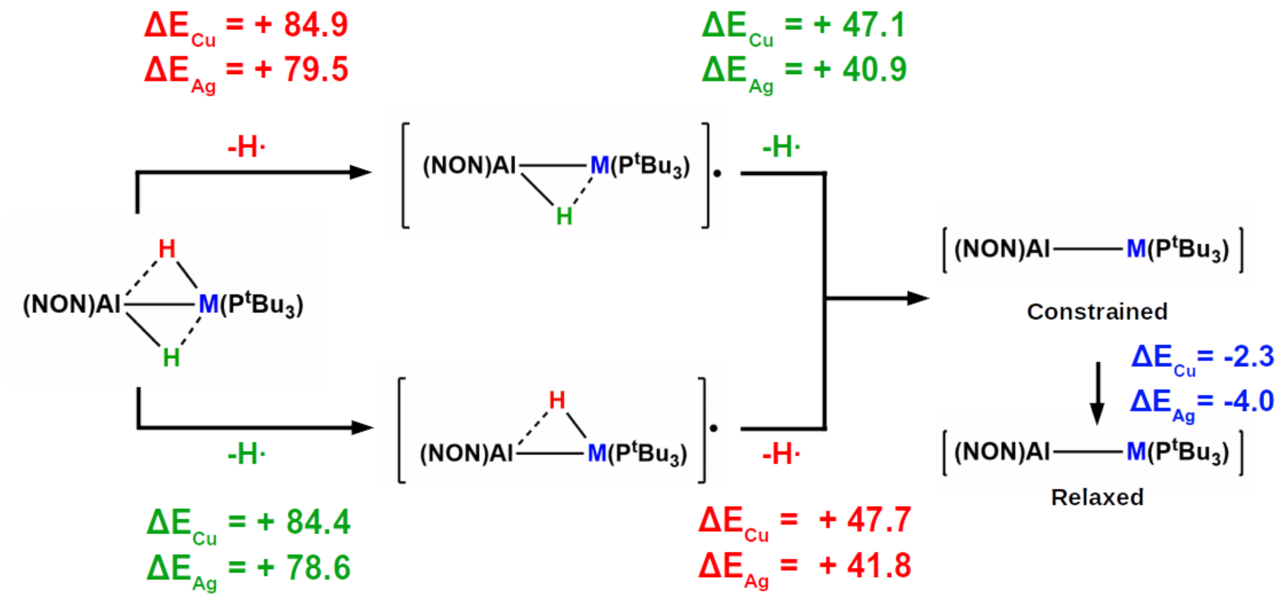

Figure S26. Pathways for the homolitic breaking of the H-substrate bonds on the doubly-bridged products [^{t}Bu$_3$PM(μ-H)Al(μ-H)(NON)] (M=Cu,Ag, **PC$_{Cu}$'**, **PC$_{Ag}$'**) and associated relative energies in kcal/mol.

		RC_{Cu}	TS^{I}_{Cu}	INT_{Cu}	TS^{II}_{Cu}	PC'_{Cu}	TS^{IV}_{Cu}	PC_{Cu}
q_{H1}		0.00	-0.08	-0.14	-0.19	-0.20	-0.08	-0.17
q_{H2}		0.02	-0.17	-0.32	-0.39	-0.20	-0.14	-0.24
q_{Cu}		-0.08	0.12	0.17	0.16	0.16	0.03	0.14
q_{Al}		0.17	0.21	0.29	0.37	0.31	0.27	0.29
		RC_{Ag}	TS^{I}_{Ag}	INT_{Ag}	TS^{II}_{Ag}	PC'_{Ag}	TS^{IV}_{Ag}	PC_{Ag}
q_{H1}		0.00	-0.08	-0.14	-0.16	-0.19	-0.07	-0.15
q_{H2}		0.01	-0.19	-0.37	-0.40	-0.20	-0.12	-0.24
q_{Ag}		-0.04	0.17	0.18	0.18	0.18	0.05	0.17
q_{Al}		0.16	0.20	0.31	0.36	0.30	0.27	0.29
	RC	TS^{I}	INT	TS^{II}	PC'	TS^{III}	TS^{IV}	PC
q_{H1}	0.00	-0.07	-0.12	-0.17	-0.14	-0.15	-0.06	-0.11
q_{H2}	0.01	-0.11	-0.25	-0.33	-0.21	-0.19	-0.12	-0.24
q_{Au}	-0.06	0.15	0.22	0.36	0.12	0.17	0.04	0.12
q_{Al}	0.18	0.19	0.22	0.17	0.32	0.30	0.27	0.30

Table S10. Evolution of the Voronoi Deformation Density (VDD) atomic charges on M, Al and two H atoms of H_2 along the pathways for the reactivity of H_2 with copper- (top), silver- (center) and gold-aluminyl complexes (bottom).

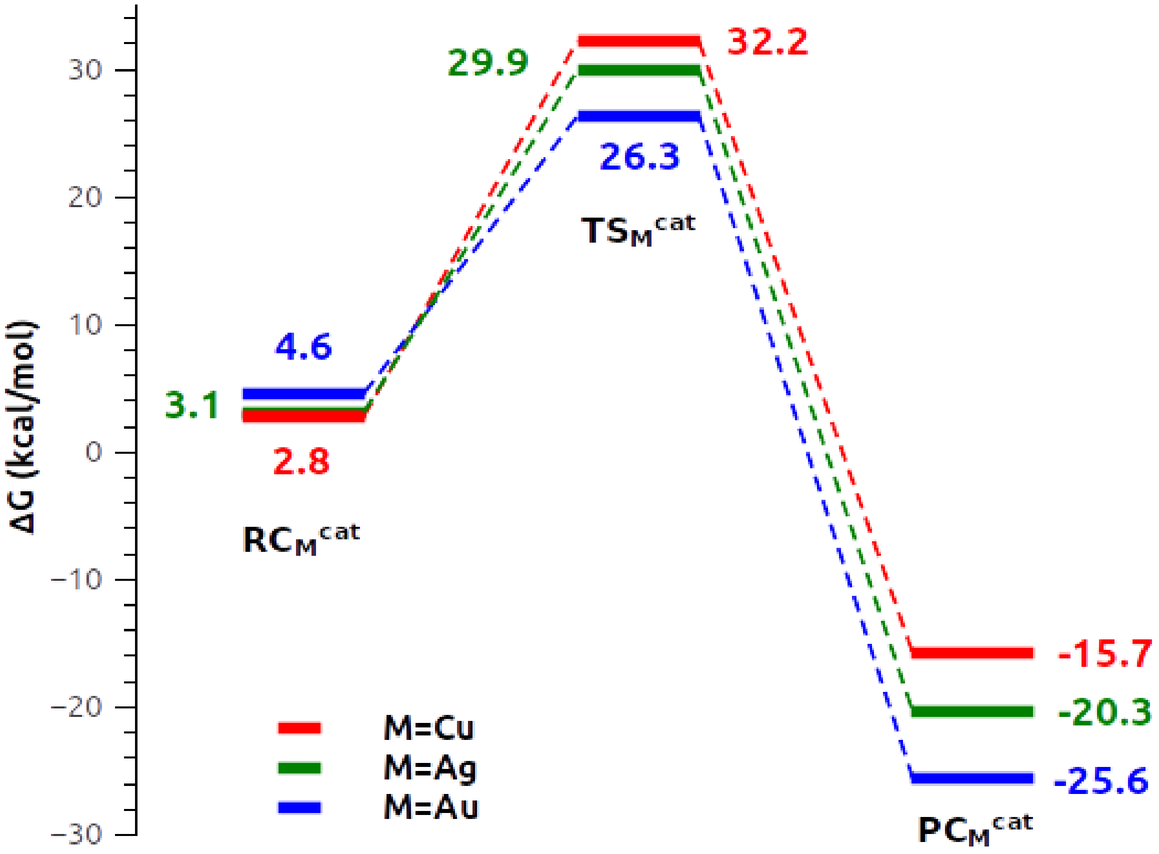

Figure S27. Free energy profile for the hydrogenation of ethylene and regeneration of catalyst [^{t}Bu$_3$PMAl(NON)] (M=Cu, Ag, Au) starting from the corresponding **PC$_M$** structures. ΔG values refer to the energy of the separated reactants (**PC$_M$** + C_2H_4) taken as zero

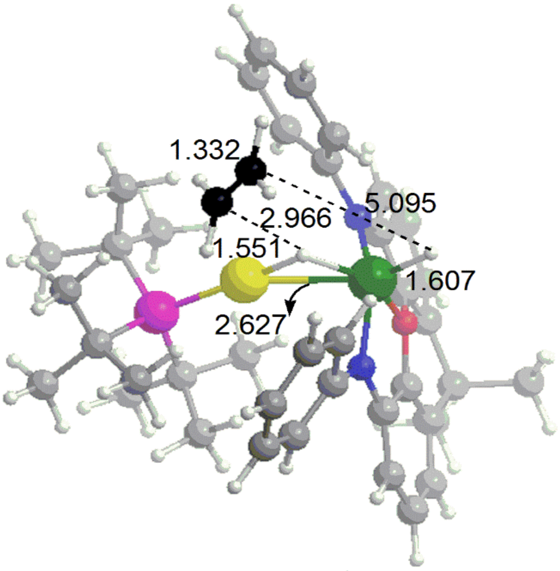

RC_{Cu}^{cat}

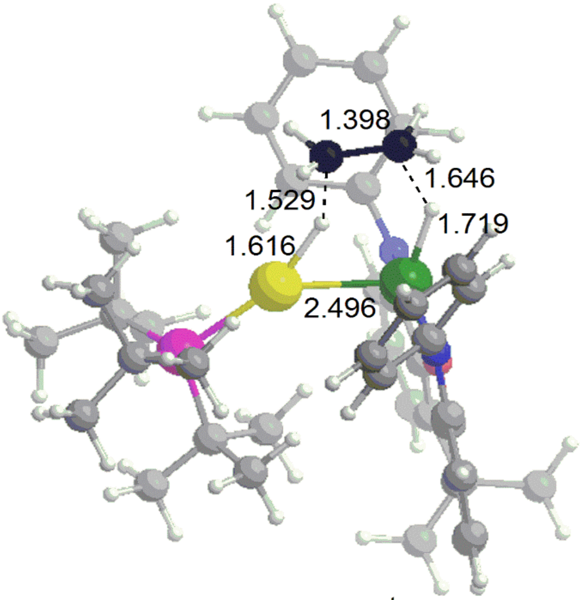

TS_{Cu}^{cat}

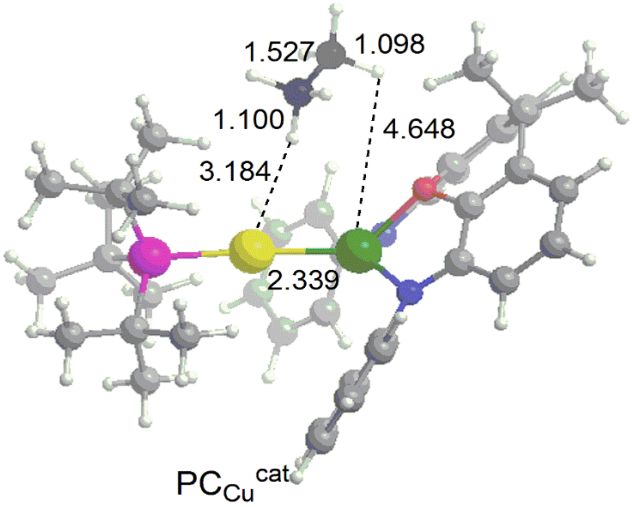

PC_{Cu}^{cat}

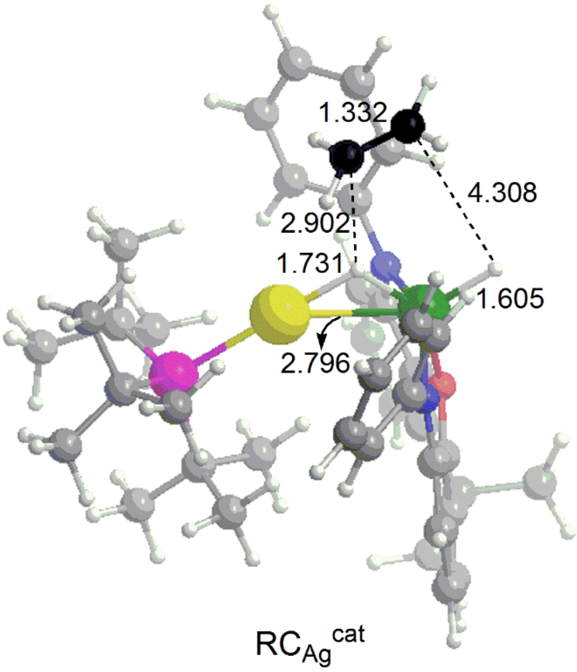

RC_{Ag}^{cat}

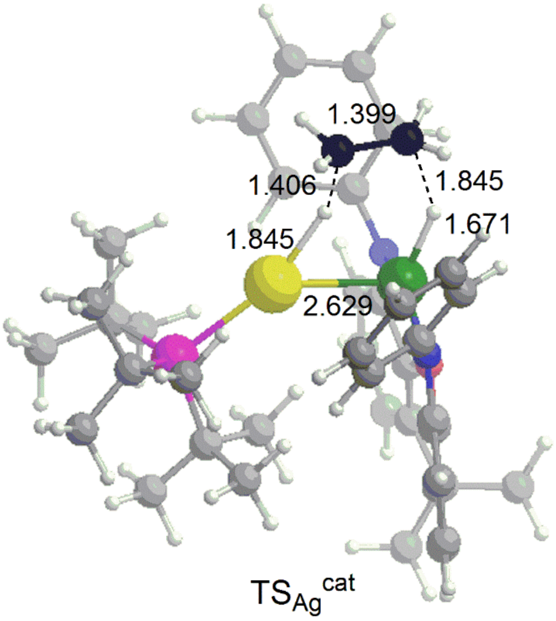

TS_{Ag}^{cat}

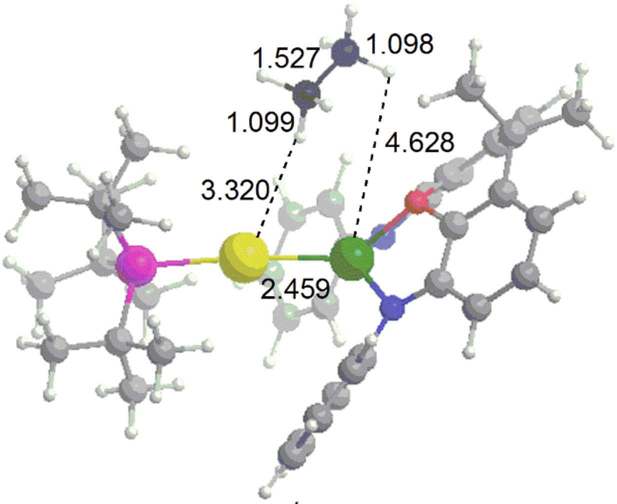

PC_{Ag}^{cat}

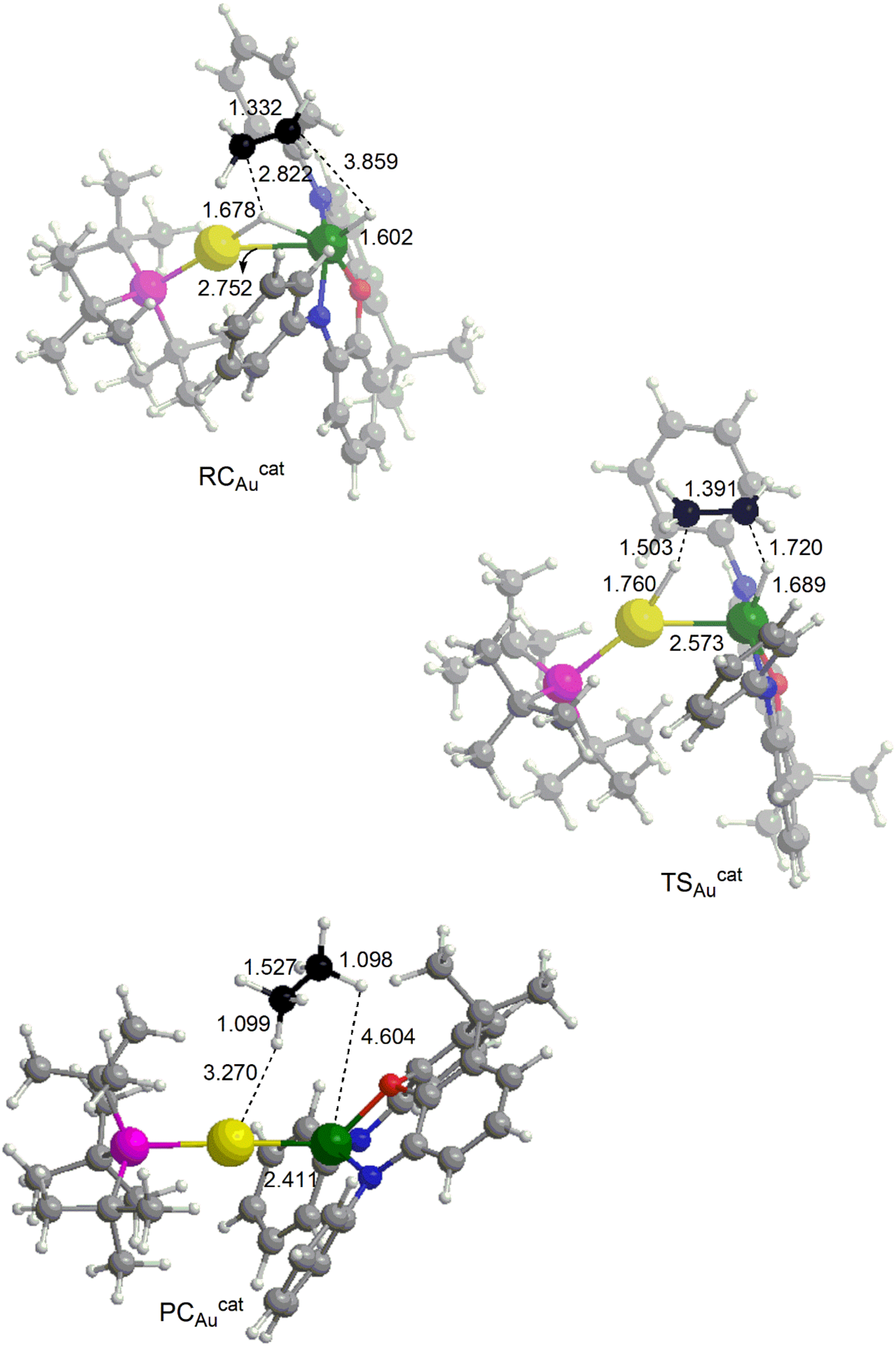

Figure S28. Optimized structures of **RC$_M^{cat}$**, **TS$_M^{cat}$** and **PC$_M^{cat}$** (M = Cu, Ag, Au) complexes. Main geometrical parameters are reported (bond in Å).

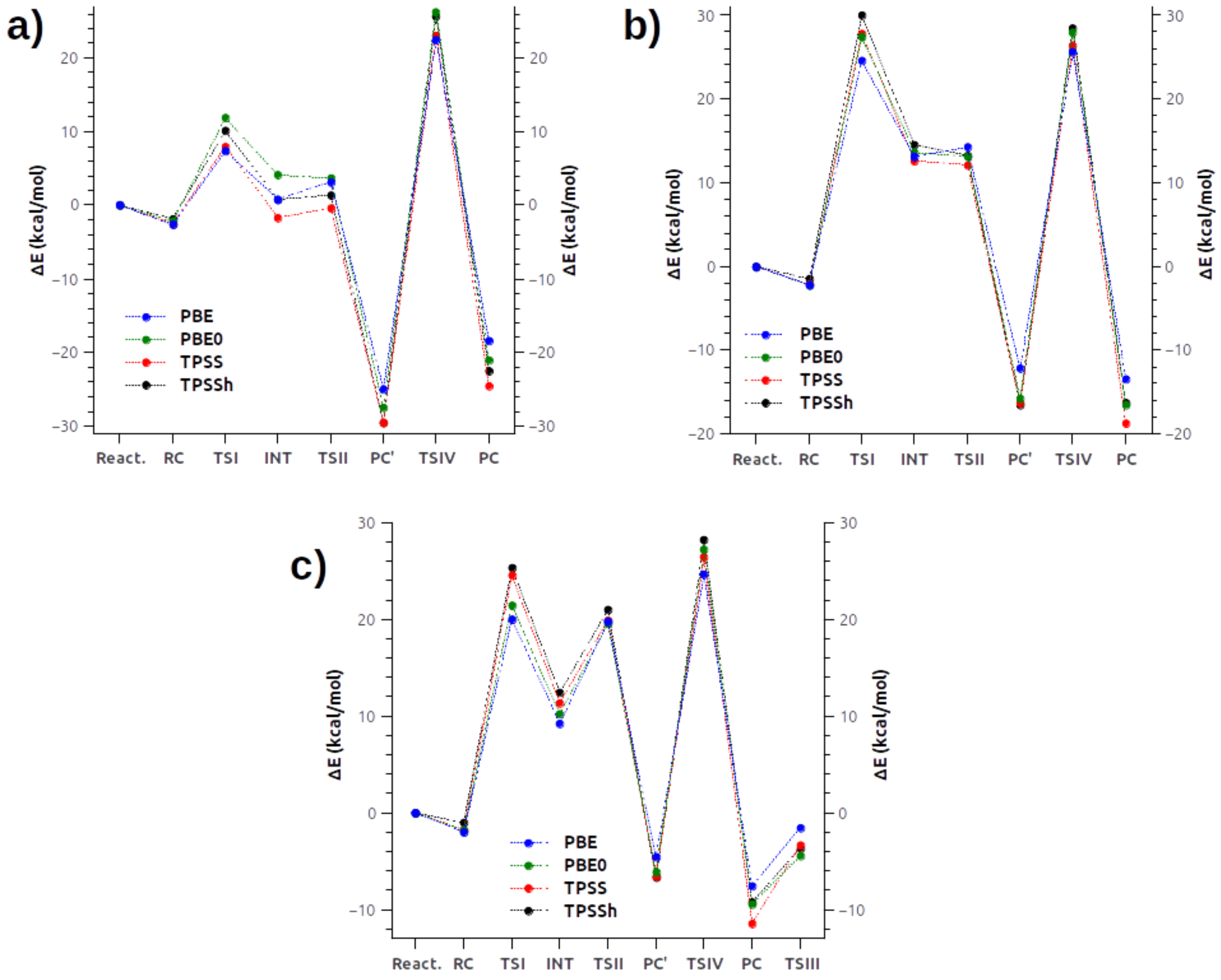

Figure S29. Relative electronic energy of the stationary points along the pathways for the H_2 mediated by copper a), silver b) and gold c) complexes with aluminyl ligands calculated with different exchange-correlation functionals (PBE, PBE0, TPSS, TPSSh). ΔE values refer to the energy of the separated reactants taken as zero.

		RC_{Cu}	TS^{I}_{Cu}	INT_{Cu}	TS^{II}_{Cu}	PC'_{Cu}	TS^{IV}_{Cu}	PC_{Cu}
**PBE**		-2.63	7.34	0.85	3.19	-24.94	22.41	-18.47
**PBE0**		-2.21	11.85	4.09	3.70	-27.49	26.30	-21.10
**TPSS**		-2.53	8.00	-1.68	-0.41	-29.53	23.11	-24.56
**TPSSh**		-1.83	10.09	0.80	1.39	-29.55	25.67	-22.44
		RC_{Ag}	TS^{I}_{Ag}	INT_{Ag}	TS^{II}_{Ag}	PC'_{Ag}	TS^{IV}_{Ag}	PC_{Ag}
**PBE**		-2.19	24.51	13.07	14.23	-12.20	25.55	-13.38
**PBE0**		-2.24	27.36	13.59	13.13	-15.69	27.92	-16.53
**TPSS**		-2.16	27.80	12.59	12.13	-16.25	26.33	-18.73
**TPSSh**		-1.47	29.92	14.51	13.25	-16.52	28.46	-16.30
	**RC**	TS^{I}	**INT**	TS^{II}	**PC'**	TS^{III}	TS^{IV}	**PC**
**PBE**	-1.99	20.01	9.24	19.76	-4.56	24.67	-7.59	-1.51
**PBE0**	-1.83	21.44	10.24	19.55	-6.12	27.17	-9.41	-4.49
**TPSS**	-1.73	24.51	11.37	19.94	-6.53	26.39	-11.41	-3.37
**TPSSh**	-1.03	25.32	12.42	20.98	-6.71	28.22	-9.23	-3.63

Table S11. Relative electronic energy of the stationary points along the pathways for the H_2 mediated by copper (top), silver (center) and gold (bottom) complexes with aluminyl ligands calculated with different exchange-correlation functionals (PBE, PBE0, TPSS, TPSSh). ΔE values refer to the energy of the separated reactants taken as zero.

References

1 M. Mitoraj and A. Michalak, *J. Mol. Model.*, 2007, **13**, 347–355.

2 A. Michalak, M. Mitoraj and T. Ziegler, *J. Phys. Chem. A*, 2008, **112**, 1933–1939.

3 R. F. Nalewajski and J. Mrozek, *Int. J. Quantum Chem.*, 1994, **51**, 187–200.

4 R. F. Nalewajski, J. Mrozek and A. Michalak, *Int. J. Quantum Chem.*, 1997, **61**, 589–601.

5 G. Bistoni, S. Rampino, F. Tarantelli and L. Belpassi, *J. Chem. Phys.*, 2015, **142**, 084112.

6 L. Belpassi, I. Infante, F. Tarantelli and L. Visscher, *J. Am. Chem. Soc.*, 2008, **130**, 1048–1060.

7 N. Salvi, L. Belpassi and F. Tarantelli, *Chem. - A Eur. J.*, 2010, **16**, 7231–7240.

8 G. Bistoni, L. Belpassi and F. Tarantelli, *J. Chem. Theory Comput.*, 2016, **12**, 1236–1244.

9 K. Morokuma, *J. Chem. Phys.*, 1971, **55**, 1236–1244.

10 T. Ziegler and A. Rauk, *Theor. Chim. Acta*, 1977, **46**, 1–10.

11 L. Zhao, M. von Hopffgarten, D. M. Andrada and G. Frenking, *Wiley Interdiscip. Rev. Comput. Mol. Sci.*, 2018, **8**, 1345.

12 M. P. Mitoraj, A. Michalak and T. Ziegler, *J. Chem. Theory Comput.*, 2009, **5**, 962–975.

13 I. Fernández and F. M. Bickelhaupt, *Chem. Soc. Rev.*, 2014, **43**, 4953–4967.

14 F. M. Bickelhaupt and K. N. Houk, *Angew. Chemie - Int. Ed.*, 2017, **56**, 10070–10086.

15 P. Vermeeren, S. C. C. van der Lubbe, C. Fonseca Guerra, F. M. Bickelhaupt and T. A. Hamlin, *Nat. Protoc.*, 2020, **15**, 649–667.

16 D. Sorbelli, L. Belpassi and P. Belanzoni, *J. Am. Chem. Soc.*, 2021, **143**, 14433–14437.

17 M. Devillard, R. Declercq, E. Nicolas, A. W. Ehlers, J. Backs, N. Saffon-Merceron, G. Bouhadir, J. C. Slootweg, W. Uhl and D. Bourissou, *J. Am. Chem. Soc.*, 2016, **138**, 4917–4926.

18 M. Hermann, C. Goedecke, C. Jones and G. Frenking, *Organometallics*, 2013, **32**, 6666–6673.

xyz geometries

96

RC_{Au}

C	-0.288695	2.379107	-2.542260
C	-1.099586	2.781152	-1.463659
C	-0.943028	4.091355	-0.971381
C	-0.019931	4.959784	-1.548965
C	0.781622	4.547899	-2.618119
C	0.642376	3.248183	-3.107094
N	-1.941097	1.828263	-0.871188
AL	-1.199089	0.124276	-0.362261
N	-1.808259	-1.600683	-0.976161
C	-0.891449	-2.452446	-1.608136
C	-0.089029	-1.926761	-2.640067
C	0.903099	-2.695905	-3.244587
C	1.115014	-4.016056	-2.845036
C	0.329172	-4.547618	-1.818274
C	-0.655368	-3.780263	-1.200843
C	-3.163904	2.160704	-0.307830
C	-3.737164	1.175792	0.511593
C	-5.015173	1.165032	1.037594
C	-5.783541	2.315451	0.806642
C	-5.241477	3.364726	0.055529
C	-3.965279	3.303765	-0.508492
O	-2.927466	0.025042	0.666043
C	-3.648023	-1.171361	0.436940
C	-4.922734	-1.289489	0.958557
C	-5.463606	-0.105642	1.772747
C	-5.603816	-2.476576	0.651211
C	-4.985773	-3.432874	-0.162911
C	-3.717680	-3.241582	-0.716435
C	-3.002982	-2.057596	-0.440061
C	-6.984097	-0.167249	1.920759
C	-4.816224	-0.126735	3.180424
AU	1.123914	0.126544	0.295638
P	3.501716	0.044411	0.726466
C	3.846036	-0.252325	2.589405
C	2.847258	0.607900	3.389640
C	4.240510	-1.395718	-0.301763
C	3.284936	-2.600618	-0.186025
C	4.291487	1.704710	0.178506
C	3.605807	2.145240	-1.131014
C	4.221028	-0.991823	-1.785760
C	5.662838	-1.816679	0.089180
C	3.931244	2.782707	1.214834
C	5.813270	1.676924	-0.012048
C	3.501860	-1.712581	2.926741
C	5.279289	0.043206	3.049338
H	-3.300735	-3.984616	-1.393461
H	-6.610813	-2.649769	1.025779
H	-6.798996	2.389245	1.191007
H	-3.606824	4.117136	-1.136383
H	-7.343671	0.684540	2.511652
H	-7.278114	-1.080894	2.452534
H	-7.486655	-0.153143	0.945045
H	-5.164410	0.735892	3.764612
H	-3.722031	-0.084206	3.118676
H	-5.098409	-1.048395	3.707241
H	-5.523912	-4.351712	-0.400039
H	-5.847361	4.254311	-0.122319
H	2.931879	0.335248	4.452903
H	3.032574	1.681357	3.305006
H	1.815492	0.412058	3.064404
H	3.520289	-1.820978	4.021762
H	2.494278	-1.979452	2.581099
H	4.221398	-2.426871	2.513955
H	5.365108	-0.207690	4.118421
H	6.022115	-0.553479	2.507821
H	5.542842	1.101365	2.941642
H	3.613492	-3.369100	-0.902019
H	3.274972	-3.053423	0.807913
H	2.256015	-2.319855	-0.450042
H	4.469760	-1.882786	-2.381778
H	3.222620	-0.654121	-2.094621
H	4.955491	-0.216510	-2.027212
H	5.995914	-2.610066	-0.598501
H	6.380437	-0.991657	0.016652
H	5.711221	-2.226716	1.104450
H	4.230826	3.759106	0.804865
H	2.848942	2.816938	1.397576
H	4.452467	2.652484	2.168811
H	6.154268	2.692366	-0.268691
H	6.344352	1.368352	0.895673
H	6.117769	1.015536	-0.831183
H	3.946546	3.164210	-1.370915
H	3.843902	1.504858	-1.983700
H	2.512878	2.173218	-1.019857
H	-0.268694	-0.904804	-2.978288
H	1.507774	-2.260476	-4.041236
H	1.885462	-4.622200	-3.321713
H	0.494959	-5.571987	-1.480722
H	-1.230247	-4.197479	-0.375314
H	-1.530046	4.416232	-0.113102
H	0.086014	5.968371	-1.146046
H	1.504282	5.232124	-3.062879
H	1.258720	2.906185	-3.939549
H	-0.410892	1.372381	-2.945635
H	-0.682247	-2.567337	2.224030
H	-0.358838	-2.041095	1.787710

96

TS^{I}_{Au}

C	2.815146	-2.126974	0.467762
C	3.521067	-1.217561	-0.334795
C	4.842092	-1.299774	-0.733120
C	5.516437	-2.479271	-0.381367
C	4.843709	-3.459481	0.357413
C	3.525594	-3.301055	0.793987
C	5.432319	-0.086423	-1.468396
C	4.883596	1.161258	-0.759793
C	3.562966	1.127642	-0.355383
O	2.801707	-0.034681	-0.608279
C	5.593854	2.328322	-0.437696
C	4.952220	3.346291	0.277989
C	3.633506	3.234800	0.727273
C	2.892149	2.070391	0.438305
N	1.640112	1.691565	0.898732
AL	0.967265	0.010004	0.231911
AU	-0.958500	0.097585	-1.289264
P	-3.279455	0.026567	-0.643857
C	-3.668872	0.718990	1.107841
C	-5.070578	0.382183	1.636898
C	6.961116	-0.112892	-1.461448
C	4.933126	-0.093809	-2.936190
N	1.565776	-1.702824	0.900772
C	0.696846	-2.583251	1.568246
C	-0.011412	-2.121994	2.691223
C	-0.928773	-2.941568	3.348134
C	-1.152362	-4.246356	2.906843
C	-0.456324	-4.714754	1.787895
C	0.451440	-3.896202	1.121257
C	0.808612	2.627802	1.544718
C	0.198415	2.284393	2.761781
C	-0.683535	3.162199	3.392672
C	-0.966916	4.404904	2.826129
C	-0.363706	4.757087	1.614215
C	0.511308	3.880639	0.977396
C	-4.285605	1.048865	-1.937090
C	-5.730902	1.368973	-1.530208
C	-3.813430	-1.814809	-0.729909
C	-5.326607	-2.041677	-0.849594
C	-3.524806	2.363564	-2.201564
C	-4.310230	0.300325	-3.279433
C	-3.095315	-2.463853	-1.929024
C	-3.298595	-2.549060	0.518588
C	-2.629887	0.184610	2.104098
C	-3.479254	2.244564	1.074631
H	3.063466	-4.063055	1.418880
H	6.557252	-2.628093	-0.662061
H	6.637630	2.439517	-0.724825
H	3.192320	4.024327	1.333545
H	7.359770	0.758752	-1.995620
H	7.328938	-1.008505	-1.977853

H	7.360794	-0.109539	-0.439222
H	5.321627	0.786030	-3.466808
H	3.837679	-0.073044	-2.986871
H	5.286913	-0.999979	-3.446473
H	5.375077	-4.372506	0.629941
H	5.510160	4.251221	0.523125
H	-4.040965	2.900133	-3.012304
H	-3.484392	3.026847	-1.334301
H	-2.496980	2.160794	-2.531694
H	-4.741651	0.976729	-4.032518
H	-3.296243	0.037216	-3.608391
H	-4.928785	-0.602689	-3.257352
H	-6.219055	1.889341	-2.368708
H	-6.316882	0.468473	-1.314431
H	-5.783650	2.035535	-0.662881
H	-3.365021	-3.530865	-1.951592
H	-3.367479	-2.025011	-2.891843
H	-2.004162	-2.393628	-1.814555
H	-3.424587	-3.629239	0.351587
H	-2.232219	-2.364644	0.696190
H	-3.860021	-2.292069	1.422727
H	-5.514388	-3.126450	-0.829024
H	-5.884617	-1.594439	-0.019848
H	-5.739398	-1.658649	-1.788983
H	-3.518256	2.614599	2.109231
H	-2.495656	2.521685	0.671227
H	-4.261732	2.761008	0.509954
H	-5.198899	0.886535	2.607320
H	-5.876661	0.720923	0.979432
H	-5.194572	-0.692587	1.813209
H	-2.766100	0.719444	3.055936
H	-2.722821	-0.884751	2.299832
H	-1.613079	0.378863	1.745776
H	0.181047	-1.111120	3.054759
H	-1.463388	-2.559018	4.218692
H	-1.862670	-4.890798	3.424837
H	-0.632947	-5.726878	1.420571
H	0.967525	-4.261374	0.233611
H	0.963863	4.150551	0.022907
H	-0.586244	5.721049	1.154222
H	-1.653279	5.093140	3.319857
H	-1.145298	2.873361	4.337900
H	0.440400	1.322690	3.217194
H	0.081841	0.145892	-2.559170
H	-1.086393	0.173357	-3.015709

96

INT_{Au}

C	-2.730359	-1.971012	-0.783526
C	-3.479253	-1.238779	0.147797
C	-4.821294	-1.379902	0.443790
C	-5.475525	-2.450880	-0.184071
C	-4.761566	-3.269068	-1.066928
C	-3.419235	-3.043878	-1.384285
C	-5.450047	-0.341259	1.385493
C	-4.847253	1.019339	1.004314
C	-3.505409	1.048355	0.679939
O	-2.767686	-0.154128	0.702098
C	-5.523922	2.246189	0.913673
C	-4.829445	3.383003	0.484598
C	-3.485565	3.345320	0.101083
C	-2.776966	2.128924	0.164699
N	-1.493820	1.838194	-0.282954
AL	-0.897691	0.036834	-0.019671
AU	0.952021	-0.348144	1.573970
P	3.203405	-0.143811	0.821522
C	3.483018	0.932806	-0.753371
C	4.879615	0.802409	-1.381713
C	-6.975186	-0.332496	1.276364
C	-5.047292	-0.678748	2.843478
N	-1.452296	-1.489020	-1.046059
C	-0.584842	-2.235219	-1.872282
C	0.055695	-1.605717	-2.949851
C	0.954607	-2.305943	-3.754916
C	1.217566	-3.653689	-3.509610
C	0.580849	-4.291123	-2.440503
C	-0.304964	-3.591550	-1.625418
C	-0.681632	2.899759	-0.747810
C	-0.196681	2.881789	-2.062866
C	0.622669	3.908239	-2.533715
C	0.962557	4.974733	-1.701283
C	0.484977	4.999698	-0.387677
C	-0.325751	3.971535	0.087923
C	4.207722	0.648745	2.268432
C	5.598245	1.170582	1.876065
C	3.851787	-1.916630	0.485586
C	5.382152	-2.043076	0.495416
C	3.367176	1.804981	2.846604
C	4.387941	-0.362807	3.413688
C	3.250654	-2.868172	1.536981
C	3.323670	-2.394639	-0.876097
C	2.447529	0.566473	-1.823343
C	3.213563	2.399618	-0.382650
H	-2.918345	-3.671479	-2.119304
H	-6.531520	-2.639449	-0.000814
H	-6.581982	2.314927	1.158486
H	-2.997808	4.241073	-0.279749
H	-7.402033	0.410181	1.962040
H	-7.382964	-1.310912	1.559772
H	-7.306117	-0.098060	0.256410
H	-5.461302	0.073144	3.528882
H	-3.957357	-0.695029	2.964844
H	-5.441541	-1.665960	3.120239
H	-5.277384	-4.100214	-1.549934
H	-5.362232	4.332684	0.418900
H	3.900769	2.210997	3.719485
H	3.208007	2.623722	2.140641
H	2.389286	1.439264	3.187891
H	4.826646	0.178894	4.265308
H	3.423819	-0.776000	3.734055
H	5.069917	-1.180918	3.160633
H	6.090554	1.539411	2.788864
H	6.237232	0.387416	1.452682
H	5.556783	2.007573	1.171897
H	3.615986	-3.883861	1.320477
H	3.527762	-2.616734	2.563319
H	2.154309	-2.878866	1.476341
H	3.514594	-3.475121	-0.953665
H	2.242251	-2.243957	-0.976490
H	3.830713	-1.913601	-1.718773
H	5.639935	-3.081056	0.233876
H	5.864358	-1.388652	-0.238110
H	5.817418	-1.838791	1.479146
H	3.198005	2.988619	-1.310220
H	2.230869	2.522269	0.091872
H	3.982481	2.827855	0.266976
H	4.943944	1.519953	-2.214234
H	5.693374	1.028919	-0.687421
H	5.048414	-0.195270	-1.802612
H	2.503119	1.316701	-2.625387
H	2.603504	-0.419004	-2.264407
H	1.433138	0.595343	-1.405954
H	-0.171478	-0.559051	-3.157513
H	1.442577	-1.794843	-4.586069
H	1.913918	-4.203716	-4.142789
H	0.788754	-5.341030	-2.229643
H	-0.781576	-4.086036	-0.778697
H	-0.689198	3.983089	1.116000
H	0.754189	5.823318	0.274923
H	1.597571	5.780463	-2.070177
H	0.988219	3.877180	-3.561061
H	-0.486428	2.058812	-2.717876
H	-0.586752	-0.495124	2.061932
H	1.483322	-0.780502	3.123400

96

TS^{II}_{Au}

C	2.939690	-2.199075	0.576217
C	3.741905	-1.308101	-0.145715
C	5.117125	-1.323859	-0.268678
C	5.762806	-2.434112	0.297879
C	5.005013	-3.409840	0.955857
C	3.619676	-3.308344	1.115415
C	5.780795	-0.126629	-0.966843

C	5.020679	1.127906	-0.509259
C	3.651434	1.027258	-0.369826
O	3.018325	-0.205034	-0.655807
C	5.572542	2.375941	-0.178368
C	4.734219	3.399347	0.278265
C	3.360140	3.220957	0.470185
C	2.776563	1.973671	0.174156
N	1.462493	1.557545	0.383546
AL	1.111493	-0.224452	-0.138313
AU	-1.256901	0.448605	-1.582090
P	-3.273373	0.106117	-0.401143
C	-3.313095	0.412624	1.498093
C	-4.503574	-0.221277	2.233342
C	7.269312	-0.033355	-0.629059
C	5.616146	-0.284840	-2.500009
N	1.599440	-1.845961	0.720898
C	0.744948	-2.800977	1.336824
C	0.207404	-2.554719	2.605273
C	-0.649879	-3.481064	3.202498
C	-0.963872	-4.671737	2.547207
C	-0.414571	-4.932266	1.288463
C	0.430561	-4.003621	0.685100
C	0.572351	2.565063	0.867727
C	0.429437	2.778715	2.243884
C	-0.373784	3.818041	2.715412
C	-1.038063	4.653995	1.815929
C	-0.911486	4.433334	0.442712
C	-0.109660	3.394379	-0.031639
C	-4.581714	1.273660	-1.210978
C	-5.934991	1.344855	-0.490115
C	-3.778782	-1.722392	-0.743315
C	-5.274550	-2.031407	-0.591010
C	-3.975790	2.687467	-1.308089
C	-4.810464	0.813896	-2.660566
C	-3.328823	-2.089584	-2.171925
C	-2.982248	-2.643367	0.193226
C	-2.006932	-0.132728	2.089349
C	-3.304090	1.922023	1.772810
H	3.072499	-4.061078	1.680677
H	6.845059	-2.532889	0.242306
H	6.643611	2.547966	-0.264438
H	2.749077	4.026794	0.873563
H	7.723485	0.822063	-1.144568
H	7.795191	-0.934693	-0.968106
H	7.431914	0.081904	0.450271
H	6.056322	0.580231	-3.013896
H	4.559295	-0.355278	-2.785012
H	6.128010	-1.196098	-2.837721
H	5.516559	-4.270869	1.388127
H	5.170863	4.369335	0.519599
H	-4.656169	3.308400	-1.911030
H	-3.850092	3.174033	-0.337993
H	-2.999744	2.654161	-1.811019
H	-5.409533	1.583604	-3.170238
H	-3.858088	0.716223	-3.198979
H	-5.363151	-0.128970	-2.727491
H	-6.607392	1.986371	-1.080907
H	-6.416493	0.366137	-0.392158
H	-5.856542	1.792900	0.506274
H	-3.537158	-3.159237	-2.330190
H	-3.847022	-1.526079	-2.951434
H	-2.248267	-1.930295	-2.296974
H	-3.144460	-3.682121	-0.131127
H	-1.904781	-2.447973	0.134367
H	-3.296489	-2.578168	1.239322
H	-5.420368	-3.111237	-0.751518
H	-5.657823	-1.791641	0.406463
H	-5.887702	-1.508722	-1.333073
H	-3.103218	2.071219	2.844318
H	-2.507385	2.429069	1.218371
H	-4.260088	2.404863	1.546972
H	-4.452590	0.078336	3.291906
H	-5.468610	0.115950	1.840972
H	-4.482593	-1.315839	2.204921
H	-2.006510	0.052471	3.174668
H	-1.869603	-1.202397	1.931277
H	-1.143937	0.398955	1.664813
H	0.469655	-1.628313	3.117295
H	-1.068900	-3.270726	4.187235
H	-1.631907	-5.395650	3.014446
H	-0.656624	-5.859703	0.768178
H	0.852105	-4.194531	-0.302334
H	0.000515	3.213829	-1.101630
H	-1.439732	5.072242	-0.265789
H	-1.662308	5.468497	2.184343
H	-0.477031	3.977197	3.789423
H	0.968077	2.130915	2.936620
H	0.326402	-0.474011	-1.577172
H	-0.886164	1.220559	-3.028914

96

PC'$_{Au}$

C	-2.855127	-2.248577	-0.499537
C	-3.588708	-1.284456	0.210386
C	-4.898572	-1.381736	0.641537
C	-5.535028	-2.609131	0.393382
C	-4.838898	-3.627005	-0.266880
C	-3.524712	-3.468484	-0.716617
C	-5.511015	-0.169659	1.356502
C	-4.958176	1.096521	0.689208
C	-3.647187	1.076653	0.252625
O	-2.882342	-0.088579	0.369849
C	-5.650542	2.302676	0.491567
C	-5.003503	3.377211	-0.127938
C	-3.686500	3.295614	-0.589615
C	-2.963267	2.098519	-0.425010
N	-1.713903	1.752882	-0.917737
AL	-1.048840	-0.023660	-0.622396
AU	1.035087	0.027405	1.077385
P	3.365835	0.037807	0.985874
C	3.956101	1.742855	0.348586
C	5.410478	1.759262	-0.143482
C	-7.040449	-0.206429	1.316169
C	-5.042059	-0.186162	2.834518
N	-1.618090	-1.828277	-0.969321
C	-0.719744	-2.769286	-1.516756
C	-0.049459	-2.480169	-2.718050
C	0.886172	-3.369169	-3.245196
C	1.162438	-4.574899	-2.597820
C	0.498867	-4.873775	-1.404784
C	-0.424064	-3.980857	-0.864658
C	-0.861989	2.754788	-1.431212
C	-0.227651	2.567453	-2.670485
C	0.660598	3.520316	-3.167089
C	0.926319	4.685603	-2.445716
C	0.300220	4.881208	-1.211785
C	-0.578104	3.925821	-0.705265
C	4.075582	-0.298283	2.732856
C	5.567621	0.027620	2.882508
C	3.902708	-1.345845	-0.225973
C	5.381801	-1.739107	-0.113491
C	3.262320	0.518698	3.755899
C	3.840054	-1.775226	3.089989
C	3.010748	-2.577808	0.021759
C	3.605357	-0.899924	-1.668025
C	3.015532	2.188351	-0.786422
C	3.790285	2.785050	1.467058
H	-3.033070	-4.270651	-1.264279
H	-6.565210	-2.771264	0.704312
H	-6.685731	2.405335	0.811240
H	-3.232281	4.139964	-1.105600
H	-7.458652	0.656602	1.848980
H	-7.415141	-1.107564	1.817309
H	-7.416037	-0.197169	0.284819
H	-5.444666	0.687100	3.365903
H	-3.947025	-0.157960	2.901643
H	-5.396531	-1.099868	3.331080
H	-5.343185	-4.575881	-0.455470
H	-5.550384	4.309777	-0.274814
H	3.596315	0.228496	4.763667
H	3.397629	1.598249	3.657717
H	2.189553	0.297129	3.670658
H	4.100738	-1.909636	4.150342
H	2.785609	-2.056321	2.967098

H	4.462931	-2.461754	2.508173
H	5.883875	-0.254906	3.898273
H	6.194016	-0.525691	2.174317
H	5.773267	1.097276	2.764320
H	3.259909	-3.336486	-0.734325
H	3.147743	-3.028427	1.007341
H	1.948368	-2.330901	-0.104836
H	3.759533	-1.771848	-2.320776
H	2.560720	-0.582784	-1.785475
H	4.272183	-0.104358	-2.016046
H	5.596909	-2.490199	-0.888716
H	6.057517	-0.892106	-0.276974
H	5.623199	-2.193514	0.853828
H	3.968133	3.777495	1.026979
H	2.771124	2.781088	1.875515
H	4.506142	2.655751	2.285376
H	5.663787	2.790484	-0.433109
H	6.122574	1.447239	0.628612
H	5.558172	1.128288	-1.026538
H	3.308956	3.202843	-1.093446
H	3.055791	1.547127	-1.668660
H	1.971034	2.232808	-0.452176
H	-0.272751	-1.545670	-3.231487
H	1.394071	-3.120957	-4.178389
H	1.886761	-5.273796	-3.017152
H	0.711132	-5.807085	-0.880892
H	-0.918739	-4.210394	0.079282
H	-1.048514	4.073747	0.267093
H	0.506096	5.782086	-0.631486
H	1.615194	5.433612	-2.839364
H	1.141606	3.352981	-4.131962
H	-0.442241	1.662348	-3.237954
H	0.362667	0.024835	-1.419051
H	-0.616610	0.001809	1.274514

96

TS^{III}_{Au}

C	-0.071818	2.867098	-1.926771
C	-0.886271	2.877179	-0.782635
C	-0.667725	3.874627	0.185068
C	0.334426	4.826433	0.012339
C	1.148699	4.800176	-1.123154
C	0.938305	3.814950	-2.089819
N	-1.853740	1.864451	-0.584000
AL	-1.321465	0.040450	-0.314737
N	-1.848608	-1.735903	-0.812048
C	-0.874252	-2.707353	-1.143251
C	-0.101229	-2.570289	-2.307107
C	0.916487	-3.478809	-2.596149
C	1.175354	-4.548416	-1.737108
C	0.404067	-4.698937	-0.581334
C	-0.606719	-3.787977	-0.283961
C	-3.142479	2.216584	-0.211749
C	-3.927408	1.180075	0.316078
C	-5.277060	1.216059	0.608172
C	-5.906688	2.457567	0.416919
C	-5.161804	3.547928	-0.046451
C	-3.804032	3.450385	-0.364085
O	-3.204030	-0.012703	0.460999
C	-3.921146	-1.183099	0.171784
C	-5.269660	-1.263578	0.458837
C	-5.935411	-0.062402	1.143777
C	-5.890542	-2.478578	0.121863
C	-5.138168	-3.501294	-0.466683
C	-3.782191	-3.356065	-0.774516
C	-3.130898	-2.142945	-0.479394
C	-7.449226	-0.052405	0.917555
C	-5.648840	-0.154108	2.664669
AU	1.242504	-0.005140	-0.004318
P	3.477719	-0.042379	0.505422
C	3.822309	1.359749	1.771111
C	3.004223	2.597295	1.351073
C	3.951874	-1.744277	1.254926
C	2.819805	-2.191914	2.199053
C	4.513564	0.260874	-1.086912
C	3.911627	-0.573997	-2.234082
C	4.000214	-2.794299	0.132581
C	5.290123	-1.751184	2.006796
C	4.337854	1.731161	-1.501668
C	6.008568	-0.057955	-0.951558
C	3.265151	0.930713	3.138811
C	5.299293	1.744453	1.926716
H	-3.236901	-4.161013	-1.265613
H	-6.950218	-2.630791	0.317421
H	-6.967627	2.577558	0.627352
H	-3.264840	4.312082	-0.755107
H	-7.908446	0.797373	1.437601
H	-7.903775	-0.963644	1.325428
H	-7.695326	0.014823	-0.149914
H	-6.088650	0.708747	3.183332
H	-4.569416	-0.163725	2.862342
H	-6.084483	-1.075775	3.073989
H	-5.630910	-4.442991	-0.712778
H	-5.661636	4.507971	-0.182763
H	3.100518	3.354526	2.144214
H	3.338292	3.050361	0.415426
H	1.938147	2.353722	1.243636
H	3.310727	1.801748	3.809446
H	2.214887	0.620385	3.061903
H	3.843765	0.126877	3.605264
H	5.377519	2.506535	2.717412
H	5.927503	0.896428	2.221826
H	5.715077	2.182830	1.012893
H	3.069726	-3.195758	2.575511
H	2.683657	-1.534201	3.060663
H	1.864536	-2.255507	1.661095
H	4.090384	-3.784568	0.604075
H	3.078928	-2.797679	-0.463739
H	4.861087	-2.668673	-0.532146
H	5.494647	-2.780656	2.338861
H	6.128429	-1.434477	1.376589
H	5.272837	-1.118830	2.901412
H	4.770595	1.854180	-2.505905
H	3.276487	2.009379	-1.557455
H	4.855136	2.428358	-0.834443
H	6.509996	0.217245	-1.892354
H	6.490875	0.503654	-0.144108
H	6.192388	-1.125750	-0.788080
H	4.421853	-0.284906	-3.165543
H	4.040053	-1.650829	-2.103234
H	2.838737	-0.368273	-2.344548
H	-0.304512	-1.735682	-2.977046
H	1.508516	-3.349946	-3.503430
H	1.971653	-5.257898	-1.963849
H	0.600208	-5.525894	0.102777
H	-1.194586	-3.895749	0.628287
H	-1.286852	3.885352	1.082694
H	0.490225	5.586700	0.779187
H	1.937067	5.542050	-1.253115
H	1.560322	3.786696	-2.985800
H	-0.237597	2.100786	-2.682907
H	-0.359287	-0.047242	1.120937
H	0.107569	0.095120	-1.385220

96

PC_{Au}

C	0.783290	2.687785	-3.265851
C	0.795932	2.778013	-1.859872
C	-0.069170	3.710568	-1.254664
C	-0.900342	4.520504	-2.024741
C	-0.902907	4.421024	-3.417829
C	-0.057874	3.492819	-4.030162
N	1.601661	1.876850	-1.140622
AL	1.665536	-0.023141	-1.666472
N	1.589462	-1.909004	-1.095368
C	0.802171	-2.824736	-1.816222
C	-0.088196	-3.737545	-1.217620
C	-0.892742	-4.567698	-1.995271
C	-0.845252	-4.506471	-3.389486
C	0.024010	-3.596605	-3.995791
C	0.839167	-2.772595	-3.223839
C	2.261214	2.243169	0.015591
C	2.957617	1.199533	0.656935
C	3.653743	1.266456	1.847062

C	3.702217	2.532401	2.456776
C	3.092601	3.623880	1.833349
C	2.385426	3.504605	0.634672
O	2.856754	-0.008794	-0.047750
C	2.924509	-1.192823	0.701333
C	3.613516	-1.232029	1.896597
C	4.371360	0.015267	2.363108
C	3.624364	-2.474193	2.555400
C	2.987226	-3.571656	1.972222
C	2.294476	-3.482164	0.762001
C	2.213302	-2.244720	0.089478
C	4.508212	0.043306	3.889480
C	5.784234	-0.021401	1.725896
AU	-0.966529	-0.012790	-0.842163
P	-2.247582	0.037937	1.107926
C	-1.153152	0.652943	2.555684
C	-0.880927	2.151916	2.352805
C	-3.716229	1.234797	0.825064
C	-4.725379	0.559021	-0.118421
C	-2.896193	-1.722040	1.486942
C	-1.737777	-2.565452	2.044812
C	-4.430609	1.671654	2.110747
C	-3.201959	2.477460	0.074431
C	-4.063956	-1.762482	2.481971
C	-3.315164	-2.383105	0.159378
C	-1.751387	0.428887	3.950295
C	0.216697	-0.041640	2.458414
H	1.829207	-4.368994	0.339127
H	4.143894	-2.591608	3.504101
H	4.230541	2.672362	3.397565
H	1.935994	4.384682	0.181262
H	5.079990	0.922635	4.209320
H	5.054214	-0.839305	4.243546
H	3.525909	0.066707	4.378994
H	6.354782	0.869361	2.022637
H	5.718675	-0.041899	0.630562
H	6.324102	-0.918970	2.057448
H	3.032520	-4.538556	2.475597
H	3.169785	4.608682	2.296462
H	-3.593171	-3.426225	0.373616
H	-4.173062	-1.900927	-0.314893
H	-2.484036	-2.396925	-0.557702
H	-2.060149	-3.617134	2.053744
H	-0.841266	-2.495729	1.417210
H	-1.471546	-2.295159	3.071365
H	-4.306421	-2.816735	2.684550
H	-3.817436	-1.290120	3.439325
H	-4.969913	-1.291570	2.085501
H	0.908203	0.459449	3.150996
H	0.198658	-1.103101	2.711768
H	0.630379	0.061693	1.448503
H	-0.106108	2.457055	3.071017
H	-0.488650	2.351441	1.348256
H	-1.763284	2.775170	2.529536
H	-1.064445	0.867070	4.690236
H	-2.725975	0.912375	4.076999
H	-1.855952	-0.633562	4.195316
H	-5.473953	1.312752	-0.403790
H	-4.242570	0.205335	-1.039295
H	-5.259596	-0.274388	0.348162
H	-5.291249	2.298100	1.831211
H	-4.811023	0.826010	2.693829
H	-3.785032	2.276910	2.757042
H	-4.072437	3.093662	-0.196891
H	-2.528258	3.097340	0.669739
H	-2.677446	2.202499	-0.850261
H	-0.170591	-3.779252	-0.134564
H	-1.576057	-5.261505	-1.502539
H	-1.477457	-5.157202	-3.994108
H	0.076815	-3.532030	-5.083674
H	1.525580	-2.074198	-3.702366
H	1.450657	1.974274	-3.749342
H	-0.045081	3.398283	-5.117025
H	-1.555809	5.056330	-4.016851
H	-1.564872	5.228663	-1.526894
H	-0.110659	3.782657	-0.170145
H	2.410627	-0.039101	-3.083818
H	-0.129737	-0.032396	-2.298752

96

TS^{IV}_{Au}

C	-0.135489	-2.504522	1.705721
C	-0.832589	-2.721444	0.507816
C	-0.544254	-3.871266	-0.242235
C	0.419271	-4.778816	0.196848
C	1.107645	-4.557198	1.391863
C	0.824263	-3.415460	2.144351
N	-1.790941	-1.773403	0.070844
AL	-1.340145	0.017279	-0.383423
AU	1.230787	0.017206	-0.413952
P	3.686114	-0.019866	-0.283215
C	4.302762	-1.358215	-1.510983
C	3.408908	-2.603729	-1.339690
C	-3.117836	-2.168155	-0.045006
C	-4.009720	-1.171401	-0.470993
C	-5.388744	-1.238331	-0.494844
C	-5.946793	-2.477369	-0.137412
C	-5.104368	-3.532183	0.229122
C	-3.712806	-3.399397	0.289875
O	-3.362718	0.031857	-0.772653
C	-4.020736	1.181196	-0.320608
C	-5.400762	1.236033	-0.335538
C	-6.156714	0.026489	-0.906003
C	-5.970510	2.415099	0.173545
C	-5.138402	3.424409	0.669023
C	-3.745459	3.300115	0.710723
C	-3.137625	2.126594	0.224878
N	-1.805965	1.734723	0.289480
C	-0.844427	2.637886	0.804645
C	-0.075446	2.274790	1.921546
C	0.897278	3.136230	2.426718
C	1.119625	4.377485	1.826818
C	0.360634	4.745394	0.713092
C	-0.612620	3.885609	0.205217
C	-7.606659	-0.013722	-0.417960
C	-6.143583	0.127833	-2.452511
H	-0.179924	0.098579	-1.632720
H	-1.173237	0.130287	-2.185564
C	4.352597	1.700948	-0.812626
C	3.469855	2.216691	-1.967450
C	4.319490	-0.430932	1.478228
C	3.408640	0.314639	2.473478
C	4.133467	2.690205	0.343851
C	5.827350	1.731607	-1.234970
C	4.105123	-1.927384	1.752184
C	5.790076	-0.085097	1.745111
C	4.054809	-0.854800	-2.942943
C	5.778445	-1.752815	-1.367184
H	-3.136770	4.089432	1.148823
H	-7.050736	2.545854	0.191186
H	-7.025667	-2.619785	-0.137176
H	-3.095363	-4.230129	0.627927
H	-8.133328	-0.871029	-0.855474
H	-8.141859	0.890447	-0.733970
H	-7.661931	-0.089579	0.675699
H	-6.652767	-0.741801	-2.890100
H	-5.116822	0.158997	-2.838258
H	-6.662404	1.041809	-2.772682
H	-5.591157	4.337664	1.057696
H	-5.547913	-4.491335	0.500246
H	3.644750	-3.309473	-2.151001
H	3.561547	-3.123639	-0.391477
H	2.343671	-2.341392	-1.410773
H	4.237700	-1.691915	-3.633360
H	3.014962	-0.528808	-3.080867
H	4.725310	-0.038602	-3.230969
H	6.020870	-2.491795	-2.147075
H	6.455135	-0.900782	-1.495714
H	5.993584	-2.220149	-0.399877
H	4.284691	-2.102856	2.824074
H	3.077194	-2.238805	1.532378
H	4.798265	-2.566574	1.195678
H	3.678539	0.000957	3.493688
H	3.503559	1.401523	2.418982

H	2.353539	0.058614	2.302564
H	6.044995	-0.407158	2.767119
H	6.469280	-0.600573	1.056319
H	5.988756	0.990219	1.684208
H	6.106165	2.773938	-1.455778
H	6.497536	1.368775	-0.447647
H	6.013521	1.147185	-2.143038
H	3.772759	3.250778	-2.192853
H	3.566489	1.632469	-2.885867
H	2.410451	2.226536	-1.674397
H	4.352234	3.701337	-0.032101
H	3.091263	2.687832	0.687237
H	4.794005	2.509003	1.197698
H	-0.261846	1.309940	2.394953
H	1.480337	2.836847	3.298487
H	1.880304	5.051379	2.221562
H	0.532501	5.708018	0.229444
H	-1.195247	4.168344	-0.672071
H	-1.074941	-4.038245	-1.180153
H	0.638097	-5.663550	-0.402793
H	1.859196	-5.269129	1.734375
H	1.347939	-3.234942	3.083936
H	-0.369021	-1.616044	2.294021

96

PC_{Au}_HH

C	2.580206	2.048606	0.104278
C	3.577724	1.150834	-0.304853
C	4.288318	1.176998	-1.489919
C	4.055153	2.284069	-2.319104
C	3.125679	3.253611	-1.929375
C	2.384524	3.151235	-0.750485
C	5.206286	-0.012952	-1.797034
C	4.444903	-1.268498	-1.352467
C	3.733970	-1.201676	-0.169334
O	3.706796	0.023883	0.550902
C	4.356395	-2.482377	-2.050477
C	3.558008	-3.513451	-1.543355
C	2.810569	-3.377320	-0.371885
C	2.866159	-2.171281	0.353477
N	2.110887	-1.770960	1.447042
AL	2.351334	0.014506	1.996767
AU	-2.676756	-0.309591	0.708017
P	-2.412288	0.085770	-1.600826
C	-0.771702	-0.666673	-2.242827
C	-0.320698	-0.170485	-3.623156
C	5.576916	-0.073005	-3.279218
C	6.501323	0.117436	-0.956111
N	1.861948	1.667820	1.231062
C	0.904259	2.513153	1.827616
C	-0.301811	1.949608	2.272081
C	-1.268423	2.728806	2.903656
C	-1.052107	4.093348	3.097759
C	0.149448	4.662825	2.665191
C	1.124126	3.885122	2.043662
C	1.307923	-2.688423	2.159811
C	0.005353	-2.314614	2.519876
C	-0.799507	-3.165917	3.275103
C	-0.320820	-4.413942	3.674126
C	0.976230	-4.795949	3.317022
C	1.788368	-3.943519	2.572749
C	-3.886714	-0.726007	-2.521634
C	-3.704030	-0.877892	-4.037615
C	-2.417407	1.976910	-1.927626
C	-2.684725	2.380575	-3.383710
C	-4.139963	-2.110487	-1.891914
C	-5.155299	0.097941	-2.244061
C	-3.472268	2.618481	-1.003921
C	-1.069156	2.566889	-1.484053
C	0.323074	-0.359500	-1.208119
C	-0.897750	-2.199318	-2.268873
H	1.626360	3.894933	-0.515702
H	4.579054	2.383235	-3.267527
H	4.889394	-2.619162	-2.989118
H	2.156199	-4.181408	-0.041093
H	6.234676	-0.929443	-3.472645
H	6.124876	0.831203	-3.572164
H	4.687555	-0.164553	-3.916021
H	7.153563	-0.747030	-1.139290
H	6.280712	0.163271	0.117109
H	7.038739	1.032571	-1.238904
H	2.950159	4.109843	-2.581963
H	3.493507	-4.451714	-2.095813
H	-5.064735	-2.519701	-2.326689
H	-3.337229	-2.827265	-2.080435
H	-4.277347	-2.027783	-0.804545
H	-6.018087	-0.470842	-2.622520
H	-5.305301	0.254978	-1.167487
H	-5.154791	1.068872	-2.749557
H	-4.629367	-1.299884	-4.459599
H	-3.517573	0.078527	-4.538194
H	-2.889006	-1.565174	-4.290629
H	-3.401746	3.711517	-1.114830
H	-4.497779	2.323782	-1.238500
H	-3.272992	2.368087	0.047161
H	-1.156096	3.663589	-1.516904
H	-0.824187	2.288756	-0.451473
H	-0.236355	2.286550	-2.136200
H	-2.601939	3.475856	-3.460996
H	-1.960099	1.944104	-4.080117
H	-3.692039	2.107287	-3.716910
H	0.106526	-2.615735	-2.439925
H	-1.257417	-2.588708	-1.307054
H	-1.548949	-2.563384	-3.070203
H	0.594464	-0.714706	-3.903859
H	-1.067536	-0.350635	-4.404110
H	-0.072799	0.896702	-3.620198
H	1.246296	-0.863707	-1.529586
H	0.542891	0.703550	-1.103621
H	0.048086	-0.756015	-0.222543
H	-0.514817	0.895635	2.074612
H	-2.201041	2.259716	3.219439
H	-1.810029	4.709131	3.582147
H	0.337278	5.725576	2.825071
H	2.069483	4.333973	1.740234
H	2.807971	-4.236797	2.322628
H	1.365910	-5.764932	3.632541
H	-0.950963	-5.085840	4.257141
H	-1.811968	-2.848682	3.527614
H	-0.407183	-1.364177	2.172264
H	-2.904899	-0.572210	2.302093
H	2.559004	0.131680	3.562003

98

[$^{t}Bu_3P(H_2)Au(\mu$-H)Al(H)(NON)]

C	-0.468299566	-3.414922570	-2.099344605
C	-0.820551626	-2.093083685	-2.430083397
C	-0.292601043	-1.539011701	-3.608571742
C	0.561747720	-2.279167877	-4.424689440
C	0.894048637	-3.594417278	-4.095754911
C	0.371398882	-4.155603108	-2.927325897
N	-1.616849286	-1.296577079	-1.580316619
C	-2.750892200	-1.830891933	-0.985654180
C	-3.306197703	-1.058279447	0.046258911
C	-4.511842482	-1.264917723	0.689820911
C	-5.217086159	-2.418313665	0.310713622
C	-4.686949127	-3.259537781	-0.674120233
C	-3.480447254	-2.987120364	-1.324724057
C	-4.939769944	-0.240869079	1.748953746
C	-4.501660744	1.145574354	1.259161511
C	-3.294022282	1.239914988	0.594343894
O	-2.551817374	0.086609997	0.324346848
C	-5.197861601	2.352057219	1.436367502
C	-4.657007390	3.540138469	0.933861615
C	-3.443908020	3.579776416	0.239472143
C	-2.717949154	2.390253839	0.034026417
N	-1.571746230	2.167943176	-0.719455374
AL	-0.919810371	0.361234160	-0.891783178
AU	0.752366452	-1.186253344	0.955860590
P	3.061046850	-0.437868301	0.822262978
C	3.798875074	-0.929446140	-0.879432429
C	5.332178880	-1.024260879	-0.886470664
C	-6.445445774	-0.296387888	2.012538744

C	-4.178794927	-0.553827809	3.063556639
C	-0.804988960	3.258614728	-1.179814333
C	-0.298619929	3.247493746	-2.490883165
C	0.529657905	4.272565147	-2.944911725
C	0.858635556	5.341805839	-2.109424930
C	0.349509238	5.368993932	-0.808204358
C	-0.465895005	4.339507466	-0.344992208
C	3.272449052	1.449364606	1.099954429
C	4.677221447	1.971541674	0.757083108
C	3.986260405	-1.385731517	2.222769848
C	5.368629621	-0.811076320	2.568416248
C	2.241233137	2.219301285	0.259357357
C	2.947613512	1.778949552	2.566881036
C	3.103871552	-1.371377160	3.487389054
C	4.158217205	-2.865822074	1.840107106
C	3.184815369	-2.279996322	-1.292406862
C	3.368061515	0.080191729	-1.955989968
H	-3.123368056	-3.644827788	-2.115212287
H	-6.172765874	-2.659401165	0.772200095
H	-6.155096543	2.368158234	1.953847605
H	-3.080922181	4.520218786	-0.170755346
H	-6.725977890	0.430128456	2.785338005
H	-6.733341344	-1.288661917	2.381756342
H	-7.020967898	-0.080387001	1.103196870
H	-4.437444560	0.186587631	3.832919898
H	-3.092954791	-0.527564712	2.906382624
H	-4.451828328	-1.554373409	3.426102228
H	-5.242662364	-4.153855231	-0.959662463
H	-5.207212977	4.471739439	1.073592304
H	2.403455164	3.293442481	0.429769131
H	2.309375440	2.040003513	-0.814590096
H	1.219200111	1.988688528	0.574743345
H	2.891466435	2.873714308	2.659387456
H	1.971861784	1.372315772	2.865164764
H	3.711213730	1.428871020	3.268584619
H	4.716411105	3.037595706	1.027813979
H	5.475074760	1.461256100	1.304564401
H	4.891714231	1.902390238	-0.314771327
H	3.662814982	-0.337013819	-2.930750286
H	2.279726369	0.224735322	-1.974448725
H	3.862750374	1.051504593	-1.851002288
H	3.608466473	-2.559517668	-2.268530606
H	3.392721325	-3.090785693	-0.589487882
H	2.097714290	-2.203070786	-1.417164213
H	5.649501112	-1.259375289	-1.913791466
H	5.812566748	-0.081901702	-0.601524930
H	5.716240666	-1.818997405	-0.238990715
H	5.826261838	-1.460512758	3.330027433
H	6.044474657	-0.786007665	1.707061711
H	5.310007077	0.195353439	2.995510096
H	3.611311607	-1.968272632	4.260367913
H	2.940784674	-0.370492058	3.893783789
H	2.126528127	-1.833420440	3.296422370
H	4.564431363	-3.389351845	2.718487967
H	3.199860253	-3.335707447	1.588117569
H	4.857919506	-3.020210566	1.013411284
H	-0.559227801	-0.516355069	-3.873583444
H	0.960869191	-1.824658218	-5.332646296
H	1.554880758	-4.175461794	-4.739597070
H	0.633043416	-5.177386252	-2.647956804
H	-0.841953338	-3.847511107	-1.171712030
H	-0.828780731	4.352887296	0.682660867
H	0.601453085	6.194282998	-0.140271307
H	1.500070997	6.146946455	-2.468678104
H	0.912108971	4.239177857	-3.966188025
H	-0.560857183	2.419848765	-3.149108328
H	-0.680137731	-1.879020864	1.151852695
H	0.023390856	0.322716671	0.790605729
H	0.395822766	0.544773026	-1.775975786
H	1.289200617	-2.682756584	1.183994989

44

[$^{t}Bu_3PAu(H_3)$]

C	-0.737502439	2.067600023	1.980548870
C	-1.537588898	1.667745898	0.725331644
C	-1.280906697	2.774462393	-0.311787830
C	-3.031756258	1.646233237	1.081815773
P	-0.852167532	0.010679444	0.019109381
C	-1.493899686	-0.227555761	-1.778999783
C	-1.290651298	-1.691074110	-2.207976525
C	-1.482908256	-1.433980184	1.119454578
C	-1.268400605	-1.068230210	2.597962747
AU	1.541882450	0.034192706	-0.160197256
C	-2.963864740	-1.781220344	0.902846349
C	-0.628680247	-2.689845677	0.867815875
C	-2.965652215	0.147941384	-1.997586868
C	-0.603905643	0.616581573	-2.713658530
H	1.570103306	-1.523624231	-0.676753114
H	-1.046222398	3.086240100	2.260201788
H	-0.925047651	1.419249700	2.839293935
H	0.339644507	2.081419737	1.776400520
H	-1.515131175	3.740001545	0.160582016
H	-0.226520961	2.799279824	-0.616689119
H	-1.913650864	2.683304893	-1.200281913
H	-3.324739069	2.662606591	1.386735359
H	-3.670846642	1.356896251	0.241614205
H	-3.246471568	0.982955120	1.926947461
H	-1.446028792	-1.973354038	3.197728739
H	-0.239927850	-0.737004058	2.791941366
H	-1.963633605	-0.300297596	2.951425644
H	-1.007521344	-3.486609379	1.525978228
H	-0.677030756	-3.052429533	-0.161466803
H	0.424363915	-2.509152737	1.110412858
H	-3.244487343	-2.556257927	1.632613130
H	-3.629255598	-0.925961715	1.057142718
H	-3.154450914	-2.192077751	-0.094401416
H	-3.228046624	-0.085977356	-3.040765734
H	-3.645909539	-0.417795223	-1.351501568
H	-3.153633681	1.216660722	-1.849276254
H	-0.919003119	0.424282168	-3.750572461
H	-0.679411303	1.691715991	-2.533888840
H	0.451675288	0.324628972	-2.621086899
H	-1.503911153	-1.754115279	-3.285646235
H	-0.255895953	-2.017267845	-2.047981818
H	-1.969365817	-2.383790021	-1.700133364
H	3.134252903	0.026519163	-0.332099614
H	1.708925028	1.596048331	0.314219646

54

[(H)Al(NON)]

C	-1.028712	3.881870	2.112047
C	-1.328683	2.550681	2.452659
C	-2.554571	2.013260	2.026037
C	-3.450181	2.774100	1.277264
C	-3.149381	4.097070	0.954190
C	-1.934755	4.642420	1.378017
N	-0.422890	1.711626	3.128349
Al	0.014864	0.001236	2.469828
N	-0.261650	-1.710077	3.208066
C	-1.078628	-2.664182	2.572353
C	-2.350058	-2.270719	2.123006
C	-3.163624	-3.150559	1.411802
C	-2.733111	-4.451157	1.151027
C	-1.471425	-4.853279	1.597006
C	-0.647474	-3.973498	2.293427
C	0.375559	2.167782	4.172113
C	1.397383	1.296881	4.576118
C	2.200891	1.410970	5.694816
C	2.041735	2.587497	6.440394
C	1.088158	3.531653	6.045225
C	0.251950	3.338910	4.944425
O	1.462515	0.101360	3.806976
C	1.506202	-1.056449	4.633043
C	2.314383	-1.041771	5.754020
C	3.126320	0.232158	6.026999
C	2.264397	-2.191051	6.555245
C	1.404597	-3.238211	6.207132
C	0.557202	-3.177496	5.099660
C	0.572390	-2.038010	4.272110
C	3.614599	0.289634	7.475201
C	4.351847	0.265537	5.079720
H	-0.142761	-3.985932	4.898649
H	2.871644	-2.264041	7.455050
H	2.637547	2.759855	7.334271

H	-0.520957	4.067257	4.707774
H	4.195984	1.204531	7.645366
H	4.276755	-0.559066	7.687967
H	2.778306	0.268403	8.185495
H	4.922660	1.190137	5.239645
H	4.047250	0.225869	4.027160
H	5.004661	-0.593994	5.283081
H	1.369672	-4.125008	6.840933
H	0.969063	4.440317	6.636375
H	-0.068869	4.306720	2.403334
H	-1.680817	5.671357	1.119665
H	-3.851660	4.697559	0.376483
H	-4.393711	2.331612	0.956132
H	-2.811200	0.990259	2.309415
H	-2.707104	-1.265896	2.358434
H	-4.145212	-2.818887	1.071723
H	-3.371106	-5.144369	0.603336
H	-1.116271	-5.862929	1.386325
H	0.348829	-4.287070	2.602857
H	0.205563	-0.026635	0.900416

102

RC$^{cat}{}_{Au}$

C	-2.189873	2.266265	-0.483370
C	-3.078143	1.206359	-0.208735
C	-4.185483	1.235304	0.614256
C	-4.465429	2.473307	1.219742
C	-3.658407	3.578072	0.940684
C	-2.541587	3.499350	0.104962
C	-5.062854	-0.014009	0.736855
C	-4.206550	-1.263662	0.510153
C	-3.094236	-1.185823	-0.304066
O	-2.714707	0.032138	-0.882666
C	-4.507390	-2.540862	1.014808
C	-3.715969	-3.631976	0.650091
C	-2.589747	-3.502103	-0.165819
C	-2.209016	-2.229989	-0.643374
N	-1.134907	-1.851092	-1.425972
AL	-0.990887	0.063127	-1.912675
AU	1.138665	0.028978	-0.171268
P	1.629126	-0.091794	2.101685
C	2.063007	1.650726	2.762159
C	2.928136	2.369570	1.709248
C	-5.762873	-0.064312	2.100232
C	-6.133173	0.041547	-0.383722
N	-1.145263	1.942345	-1.325480
C	-0.132918	2.861188	-1.652799
C	0.430608	3.768467	-0.734067
C	1.484321	4.601510	-1.104115
C	2.016739	4.550065	-2.394148
C	1.475749	3.645555	-3.310319
C	0.416437	2.816950	-2.949497
C	-0.100580	-2.738001	-1.770248
C	0.549489	-2.551978	-3.007825
C	1.639714	-3.335578	-3.375360
C	2.112508	-4.340525	-2.528827
C	1.479384	-4.536083	-1.299645
C	0.394411	-3.748531	-0.920847
C	0.089692	-0.774338	3.018238
C	-1.164838	-0.082706	2.452839
C	3.119982	-1.274109	2.322305
C	2.934766	-2.479502	1.381632
C	-0.056645	-2.262027	2.663361
C	0.129641	-0.613844	4.543720
C	4.393099	-0.556357	1.843522
C	3.323888	-1.769913	3.759947
C	0.764864	2.466114	2.884781
C	2.782396	1.651311	4.117977
H	-1.954987	4.391872	-0.097668
H	-5.319674	2.581590	1.884771
H	-5.367116	-2.688871	1.664974
H	-2.016203	-4.384992	-0.435131
H	-6.414887	-0.943245	2.167894
H	-6.399648	0.817503	2.239564
H	-5.034708	-0.103133	2.920825
H	-6.773340	-0.850095	-0.338300
H	-5.660959	0.078858	-1.373825
H	-6.758680	0.936775	-0.264208
H	-3.907525	4.541832	1.387282
H	-3.983842	-4.625483	1.012931
H	3.082566	3.405345	2.048070
H	3.911876	1.914959	1.570832
H	2.421833	2.406379	0.735900
H	1.041804	3.515801	3.062673
H	0.170653	2.428301	1.963506
H	0.138986	2.144943	3.723180
H	2.915288	2.696611	4.436245
H	2.209834	1.138167	4.898653
H	3.778908	1.199490	4.064019
H	-2.050551	-0.613747	2.830803
H	-1.258714	0.968465	2.731843
H	-1.183443	-0.149993	1.358335
H	-1.036591	-2.603914	3.026813
H	-0.039900	-2.412624	1.577326
H	0.709361	-2.890936	3.128218
H	-0.768905	-1.093102	4.961005
H	1.002327	-1.095644	4.997793
H	0.111484	0.436541	4.854694
H	5.205987	-1.297038	1.820524
H	4.274876	-0.160009	0.826070
H	4.705930	0.254694	2.508510
H	4.235996	-2.385290	3.786183
H	3.454760	-0.951548	4.476399
H	2.496275	-2.401587	4.101427
H	3.853340	-3.084250	1.421013
H	2.099197	-3.124894	1.659137
H	2.779976	-2.161002	0.342056
H	0.059395	3.800907	0.287355
H	1.903802	5.289245	-0.367788
H	2.841557	5.203151	-2.679839
H	1.878930	3.586757	-4.322795
H	-0.001613	2.115159	-3.671040
H	0.186084	-1.774317	-3.679780
H	2.120766	-3.161087	-4.339309
H	2.961771	-4.958957	-2.820008
H	1.842804	-5.304565	-0.615202
H	-0.051955	-3.900190	0.058691
H	-1.159094	0.101886	-3.505820
H	0.888238	0.087239	-1.828968
C	2.933319	0.191885	-3.770933
C	2.401793	0.179110	-4.991933
H	3.189538	-0.731677	-3.249952
H	3.105035	1.125073	-3.232749
H	2.123826	1.100562	-5.506687
H	2.209065	-0.754256	-5.524261

102

TS$^{cat}{}_{Au}$

C	-2.316842	2.266457	-0.032248
C	-3.045889	1.076683	0.166158
C	-4.071767	0.869989	1.065042
C	-4.428969	1.990944	1.839402
C	-3.757885	3.205044	1.671748
C	-2.712628	3.362695	0.755933
C	-4.785638	-0.484051	1.143344
C	-3.950913	-1.572269	0.457466
C	-2.927422	-1.242325	-0.408014
O	-2.560637	0.072003	-0.660095
C	-4.201090	-2.950951	0.597416
C	-3.446647	-3.878813	-0.124267
C	-2.415407	-3.495898	-0.987468
C	-2.111782	-2.129881	-1.139556
N	-1.156983	-1.511563	-1.921860
AL	-0.799945	0.416825	-1.698911
AU	1.331259	0.201005	-0.273540
P	1.822118	-0.504704	1.980139
C	2.451889	0.984651	3.011882
C	3.351600	1.842549	2.099796
C	-5.051657	-0.862761	2.611281
C	-6.137611	-0.366313	0.397141
N	-1.326814	2.175981	-0.986471
C	-0.442906	3.265218	-1.172586

C	0.406740	3.701683	-0.142611
C	1.339573	4.711213	-0.366977
C	1.444236	5.304449	-1.628232
C	0.596985	4.885073	-2.655613
C	-0.342985	3.878574	-2.428527
C	-0.217798	-2.288696	-2.635446
C	-0.008528	-2.034009	-4.000059
C	0.973444	-2.722335	-4.712248
C	1.757926	-3.689521	-4.081097
C	1.547492	-3.961095	-2.726362
C	0.574674	-3.267317	-2.011129
C	0.235877	-1.220204	2.780926
C	-0.928651	-0.264678	2.461140
C	3.186696	-1.852540	1.900238
C	2.871425	-2.789692	0.718587
C	-0.094102	-2.545569	2.073440
C	0.310069	-1.452011	4.295085
C	4.523385	-1.176360	1.550687
C	3.350914	-2.685191	3.178298
C	1.257781	1.873887	3.393768
C	3.207392	0.606350	4.292814
H	-2.203739	4.320012	0.653123
H	-5.236404	1.919237	2.566341
H	-4.995433	-3.299213	1.255698
H	-1.850056	-4.246615	-1.536557
H	-5.582835	-1.819996	2.675427
H	-5.678698	-0.107108	3.099632
H	-4.112670	-0.948015	3.173125
H	-6.676723	-1.322835	0.433694
H	-5.976837	-0.098986	-0.655303
H	-6.761198	0.410125	0.860918
H	-4.058824	4.062352	2.275947
H	-3.669960	-4.941158	-0.014687
H	3.633726	2.752260	2.651882
H	4.272394	1.337384	1.797679
H	2.813848	2.147868	1.190999
H	1.653410	2.820256	3.791549
H	0.630311	2.111782	2.526726
H	0.628494	1.427563	4.170264
H	3.465646	1.531792	4.830659
H	2.601084	-0.010428	4.966216
H	4.144318	0.077014	4.089520
H	-1.872615	-0.796144	2.644361
H	-0.933875	0.641666	3.070378
H	-0.913685	0.034755	1.405577
H	-1.103947	-2.856779	2.378531
H	-0.109659	-2.423797	0.982599
H	0.596816	-3.352504	2.338831
H	-0.642032	-1.896374	4.624351
H	1.112484	-2.140958	4.579508
H	0.441956	-0.517877	4.852740
H	5.258727	-1.968562	1.345221
H	4.438035	-0.559883	0.645395
H	4.922428	-0.561281	2.362965
H	4.197077	-3.375780	3.038243
H	3.564857	-2.071872	4.060383
H	2.465237	-3.296403	3.386289
H	3.731809	-3.461032	0.575446
H	1.989885	-3.412637	0.886649
H	2.717560	-2.227424	-0.212430
H	0.330106	3.226566	0.834102
H	1.998674	5.028227	0.442915
H	2.180297	6.088727	-1.807336
H	0.665972	5.345630	-3.642103
H	-1.008233	3.545211	-3.225052
H	-0.629556	-1.284915	-4.490165
H	1.120902	-2.505469	-5.771349
H	2.523869	-4.228871	-4.638973
H	2.156877	-4.710360	-2.218220
H	0.429098	-3.467662	-0.950896
H	-0.174563	0.847373	-3.207430
H	1.602221	0.776882	-1.913977
C	2.285522	1.264676	-3.160711
C	1.237732	1.307298	-4.074748
H	3.005891	0.446831	-3.211034
H	2.658053	2.197941	-2.734769
H	0.760313	2.251127	-4.328281
H	1.078265	0.485159	-4.770482

102

PC$^{cat}{}_{Au}$

C	0.783901	-3.724995	1.024572
C	0.993376	-2.460041	1.607199
C	0.218938	-2.109428	2.728827
C	-0.727215	-2.988866	3.251589
C	-0.918973	-4.244524	2.673743
C	-0.157286	-4.602404	1.556985
N	1.854289	-1.497291	1.058389
AL	1.129640	0.227470	0.585146
AU	-1.189845	0.202835	-0.074076
P	-3.546451	0.163778	-0.601573
C	-4.423643	1.530465	0.419038
C	-4.457954	1.088916	1.892059
O	2.847125	0.310932	-0.464380
C	3.640568	-0.852774	-0.338856
C	4.905026	-0.860231	-0.894760
C	5.360329	0.412789	-1.621415
C	4.854606	1.596892	-0.783816
C	3.588497	1.493444	-0.238680
C	5.649716	-2.033934	-0.707028
C	5.098795	-3.086518	0.033483
C	3.838086	-3.006932	0.629069
C	3.061962	-1.840801	0.470997
C	2.968046	2.383975	0.651925
C	3.702979	3.556835	0.922144
C	4.965162	3.732502	0.349628
C	5.556958	2.772612	-0.480013
C	6.878328	0.451853	-1.799011
C	4.685178	0.458146	-3.015735
N	1.773791	1.939838	1.200952
C	0.856515	2.799445	1.821185
C	0.633140	4.128491	1.411297
C	-0.372305	4.892736	1.998516
C	-1.189128	4.358034	2.999404
C	-0.983700	3.039160	3.406957
C	0.028744	2.273159	2.833010
C	-3.819273	0.481132	-2.473154
C	-3.540784	1.965660	-2.761409
C	-4.257287	-1.554100	-0.130273
C	-3.777528	-2.581273	-1.169753
C	-2.738662	-0.303704	-3.244173
C	-5.210229	0.116815	-3.007416
C	-3.609129	-1.981202	1.201755
C	-5.785218	-1.626666	-0.015531
C	-3.537590	2.792826	0.385119
C	-5.846320	1.877099	-0.037626
H	3.469203	-3.827805	1.241221
H	6.653682	-2.124392	-1.117186
H	6.559767	2.935255	-0.870313
H	3.306642	4.302833	1.608391
H	7.176790	1.366596	-2.326489
H	7.212525	-0.398957	-2.405928
H	7.399421	0.418839	-0.833506
H	4.967423	1.381904	-3.538738
H	3.592057	0.427118	-2.932361
H	5.010003	-0.402944	-3.615357
H	5.685456	-3.995235	0.175382
H	5.519892	4.642452	0.582712
H	-4.027305	-3.584837	-0.793533
H	-4.263655	-2.464498	-2.143710
H	-2.688076	-2.537802	-1.302456
H	-3.889185	-3.026739	1.401720
H	-2.513161	-1.932108	1.144578
H	-3.931906	-1.380546	2.055508
H	-6.072131	-2.666734	0.206196
H	-6.170451	-1.003162	0.799235
H	-6.290920	-1.333353	-0.942602
H	-2.780267	0.001098	-4.301266
H	-1.735737	-0.074143	-2.857190
H	-2.877106	-1.386710	-3.203985
H	-3.512957	2.100254	-3.853269
H	-4.317245	2.629477	-2.367690
H	-2.567275	2.277161	-2.359572
H	-5.261163	0.389484	-4.073423

H	-5.414995	-0.957366	-2.936409
H	-6.011572	0.655057	-2.488560
H	-3.955716	3.523098	1.094724
H	-2.510448	2.567037	0.703825
H	-3.496796	3.269035	-0.597234
H	-6.263054	2.630119	0.649868
H	-5.866206	2.310947	-1.043890
H	-6.514940	1.008898	-0.024084
H	-4.792094	1.947137	2.494535
H	-5.155537	0.265234	2.075392
H	-3.458769	0.801896	2.246483
H	1.231258	4.549001	0.603909
H	-0.529492	5.917216	1.657105
H	-1.976712	4.961283	3.451040
H	-1.611555	2.601586	4.184398
H	0.198650	1.250278	3.174258
H	0.382200	-1.137124	3.197020
H	-1.314580	-2.690087	4.120852
H	-1.655565	-4.935218	3.084367
H	-0.308155	-5.573992	1.083776
H	1.345013	-4.003101	0.132771
C	0.945320	-2.028570	-2.623206
C	2.010681	-3.085417	-2.905243
H	0.090648	-2.120455	-3.308280
H	2.867488	-2.978001	-2.226633
H	2.389607	-3.008858	-3.934334
H	1.347463	-1.011732	-2.736494
H	1.609729	-4.101057	-2.775845
H	0.552996	-2.105314	-1.599463

96

RC_{Cu}

C	-0.004180	-2.312599	-2.512593
C	0.792319	-2.713856	-1.422138
C	0.623316	-4.022460	-0.927560
C	-0.294599	-4.889759	-1.514729
C	-1.077087	-4.480257	-2.599313
C	-0.927976	-3.182257	-3.089477
N	1.630136	-1.763285	-0.824307
AL	0.867635	-0.057384	-0.301381
N	1.502813	1.662767	-0.947642
C	0.588014	2.510204	-1.583863
C	-0.230197	1.972195	-2.598412
C	-1.219631	2.739595	-3.210354
C	-1.416095	4.068956	-2.834948
C	-0.617404	4.612314	-1.823836
C	0.365084	3.848080	-1.199945
C	2.855263	-2.097031	-0.269651
C	3.441271	-1.108276	0.536762
C	4.726530	-1.099709	1.045607
C	5.488123	-2.254727	0.814360
C	4.932794	-3.307606	0.078253
C	3.650053	-3.245200	-0.470578
O	2.638878	0.045721	0.692549
C	3.360046	1.235711	0.442386
C	4.643129	1.353988	0.944187
C	5.190612	0.175785	1.762173
C	5.326369	2.534196	0.615258
C	4.702227	3.485094	-0.200431
C	3.425102	3.294633	-0.733022
C	2.706861	2.118039	-0.433254
C	6.713294	0.232650	1.887597
C	4.564120	0.213163	3.178880
CU	-1.374095	-0.051898	0.386292
P	-3.559868	0.005848	0.864190
C	-3.851041	0.323060	2.733543
C	-2.804928	-0.508272	3.503142
C	-4.355132	1.418972	-0.157950
C	-3.402130	2.629156	-0.085916
C	-4.341240	-1.674471	0.367040
C	-3.675377	-2.114157	-0.952157
C	-4.381094	0.998577	-1.637133
C	-5.767328	1.832537	0.274163
C	-3.937215	-2.735658	1.404430
C	-5.866923	-1.675015	0.213299
C	-3.527961	1.792940	3.048847
C	-5.261244	0.002468	3.244770
H	3.003467	4.032088	-1.413295
H	6.339577	2.705551	0.973705
H	6.508187	-2.329125	1.186315
H	3.281274	-4.061476	-1.088731
H	7.078522	-0.615346	2.480498
H	7.018586	1.149833	2.406815
H	7.201315	0.208037	0.904762
H	4.919180	-0.643996	3.766992
H	3.469090	0.172122	3.132980
H	4.855593	1.139691	3.692007
H	5.242158	4.398012	-0.455894
H	5.532899	-4.201011	-0.100456
H	-2.852602	-0.228164	4.566869
H	-2.969395	-1.586272	3.434579
H	-1.789263	-0.293614	3.139050
H	-3.520559	1.910540	4.143144
H	-2.535631	2.078587	2.675937
H	-4.272362	2.488782	2.648706
H	-5.316646	0.261050	4.314023
H	-6.033020	0.580190	2.723283
H	-5.507944	-1.061227	3.153987
H	-3.755921	3.391341	-0.796494
H	-3.358120	3.090030	0.903258
H	-2.380850	2.352359	-0.385137
H	-4.663437	1.878810	-2.234335
H	-3.389193	0.672601	-1.978494
H	-5.111113	0.209460	-1.845098
H	-6.129583	2.616665	-0.409387
H	-6.478758	0.999910	0.231668
H	-5.787834	2.250621	1.287026
H	-4.237003	-3.720390	1.014825
H	-2.849383	-2.756794	1.554886
H	-4.429945	-2.600470	2.372634
H	-6.196525	-2.697670	-0.029192
H	-6.380682	-1.369754	1.132147
H	-6.202852	-1.023697	-0.601488
H	-3.994529	-3.143547	-1.176689
H	-3.944411	-1.488300	-1.806373
H	-2.579326	-2.118262	-0.865619
H	-0.059189	0.944069	-2.921598
H	-1.834254	2.295090	-3.994374
H	-2.183380	4.673594	-3.318662
H	-0.771171	5.644387	-1.504260
H	0.950348	4.276083	-0.387361
H	1.198823	-4.346413	-0.061045
H	-0.409645	-5.896667	-1.110052
H	-1.793558	-5.164744	-3.053646
H	-1.529656	-2.842304	-3.933544
H	0.130004	-1.308920	-2.919292
H	0.112681	2.753714	2.147906
H	-0.042688	2.169069	1.692675

96

TS^{I}_{Cu}

C	-2.626729	2.110404	0.344279
C	-3.309961	1.191715	-0.467123
C	-4.629286	1.252975	-0.874843
C	-5.328753	2.415813	-0.516804
C	-4.681112	3.402120	0.235850
C	-3.362073	3.266797	0.677217
C	-5.190457	0.039723	-1.631690
C	-4.621908	-1.207640	-0.939332
C	-3.305514	-1.155099	-0.522717
O	-2.564097	0.026062	-0.745065
C	-5.311920	-2.393810	-0.644199
C	-4.657499	-3.411697	0.059419
C	-3.344782	-3.283109	0.521323
C	-2.623039	-2.100936	0.257186
N	-1.379993	-1.707480	0.731031
AL	-0.732140	0.006330	0.121073
CU	1.222111	-0.035134	-1.227282
P	3.383462	0.001619	-0.766782
C	3.888875	-0.673962	0.962613
C	5.327006	-0.344798	1.387826
C	-6.719495	0.035513	-1.635128

C	-4.681401	0.080879	-3.095462
N	-1.369593	1.709790	0.777113
C	-0.514645	2.602145	1.446859
C	0.212415	2.143387	2.559150
C	1.122247	2.972689	3.214333
C	1.319018	4.284910	2.782654
C	0.603799	4.751110	1.674883
C	-0.296104	3.922908	1.009251
C	-0.541121	-2.633166	1.382583
C	0.059452	-2.279037	2.601835
C	0.944557	-3.146687	3.242105
C	1.241235	-4.389816	2.683307
C	0.650803	-4.751164	1.467995
C	-0.227087	-3.884646	0.821273
C	4.275986	-1.048327	-2.114918
C	5.740218	-1.395918	-1.815395
C	3.921289	1.837824	-0.910894
C	5.419924	2.054429	-1.158666
C	3.458882	-2.342718	-2.302171
C	4.211118	-0.314595	-3.464280
C	3.103104	2.469925	-2.053557
C	3.514910	2.589413	0.366980
C	2.921843	-0.117934	2.018960
C	3.683197	-2.197841	0.961063
H	-2.917605	4.032906	1.309852
H	-6.370059	2.546922	-0.804504
H	-6.350726	-2.519949	-0.942909
H	-2.894096	-4.074319	1.118149
H	-7.096926	-0.835711	-2.185078
H	-7.101844	0.931326	-2.140371
H	-7.125812	0.008374	-0.615884
H	-5.046466	-0.799030	-3.642342
H	-3.585419	0.085096	-3.138571
H	-5.051482	0.986800	-3.594390
H	-5.232759	4.301189	0.514277
H	-5.200044	-4.331185	0.284009
H	3.887580	-2.901932	-3.148029
H	3.469779	-2.997850	-1.427758
H	2.413924	-2.106526	-2.550086
H	4.575572	-1.005406	-4.239525
H	3.177806	-0.040170	-3.714100
H	4.842324	0.579389	-3.498787
H	6.150997	-1.940379	-2.679679
H	6.359306	-0.504462	-1.661533
H	5.847464	-2.048451	-0.942314
H	3.356659	3.539946	-2.107490
H	3.300178	2.023094	-3.030747
H	2.024159	2.390841	-1.852652
H	3.638056	3.666819	0.180213
H	2.464182	2.418234	0.630885
H	4.143469	2.333651	1.226154
H	5.614450	3.138076	-1.179506
H	6.040356	1.622785	-0.365349
H	5.754484	1.645250	-2.117943
H	3.794975	-2.558052	1.993939
H	2.670027	-2.469658	0.634732
H	4.416120	-2.728856	0.345577
H	5.519416	-0.832522	2.356155
H	6.080382	-0.705349	0.680732
H	5.475971	0.731360	1.533590
H	3.098354	-0.657428	2.961699
H	3.051713	0.948827	2.210046
H	1.880939	-0.285796	1.717941
H	0.039930	1.126808	2.916670
H	1.672170	2.590997	4.075603
H	2.023030	4.937155	3.299454
H	0.759109	5.769526	1.315495
H	-0.826312	4.287283	0.129571
H	-0.669573	-4.162885	-0.135544
H	0.885266	-5.714548	1.012859
H	1.928703	-5.070647	3.185545
H	1.398429	-2.849806	4.188602
H	-0.194704	-1.318326	3.052807
H	-0.015505	-0.031266	-2.044036
H	1.079510	-0.065788	-2.797828

96

INT_{Cu}

C	-2.541282	2.125368	0.309296
C	-3.311499	1.220267	-0.431901
C	-4.663293	1.293087	-0.707112
C	-5.308572	2.464704	-0.281456
C	-4.575905	3.444083	0.398742
C	-3.221508	3.292265	0.710828
C	-5.307496	0.087659	-1.409197
C	-4.677070	-1.169168	-0.790898
C	-3.325972	-1.126635	-0.509626
O	-2.602313	0.052849	-0.784769
C	-5.333241	-2.361600	-0.445381
C	-4.609889	-3.391230	0.166901
C	-3.255166	-3.271311	0.492927
C	-2.567113	-2.083911	0.175091
N	-1.267646	-1.711540	0.508460
AL	-0.724342	0.017063	-0.086267
CU	1.254658	0.033652	-1.539332
P	3.322765	-0.018240	-0.718943
C	3.622887	-0.717201	1.053920
C	5.009330	-0.424031	1.647779
C	-6.829400	0.090587	-1.256529
C	-4.949451	0.137524	-2.916140
N	-1.244022	1.717500	0.613698
C	-0.391037	2.631258	1.278829
C	0.183205	2.285630	2.509893
C	1.046022	3.166672	3.164482
C	1.333523	4.413716	2.609350
C	0.759997	4.768503	1.384583
C	-0.088033	3.885465	0.721070
C	-0.458924	-2.680818	1.157559
C	-0.060372	-2.489524	2.486839
C	0.727918	-3.441730	3.134611
C	1.119448	-4.602432	2.466762
C	0.727889	-4.798798	1.139440
C	-0.051582	-3.845196	0.487440
C	4.328585	-1.107424	-1.957440
C	5.723541	-1.529150	-1.474250
C	3.984700	1.784169	-0.778722
C	5.514071	1.906795	-0.843811
C	3.472697	-2.355317	-2.253630
C	4.490719	-0.375609	-3.300969
C	3.357931	2.479549	-2.001462
C	3.481283	2.553000	0.451939
C	2.558082	-0.144057	1.997760
C	3.393572	-2.235445	1.025645
H	-2.703201	4.055350	1.288808
H	-6.371635	2.609126	-0.464071
H	-6.397143	-2.484370	-0.638627
H	-2.742050	-4.075409	1.017807
H	-7.264808	-0.774051	-1.772867
H	-7.255851	0.992281	-1.713511
H	-7.130593	0.056382	-0.201609
H	-5.373159	-0.735819	-3.430180
H	-3.863530	0.135995	-3.070143
H	-5.361879	1.049466	-3.368507
H	-5.085147	4.352339	0.723743
H	-5.126618	-4.316083	0.426834
H	3.978362	-2.944584	-3.034161
H	3.328846	-3.002375	-1.384595
H	2.487635	-2.057250	-2.642065
H	4.919599	-1.092461	-4.017883
H	3.518707	-0.043278	-3.685310
H	5.174383	0.477860	-3.244650
H	6.206146	-2.099405	-2.283008
H	6.365640	-0.669507	-1.250520
H	5.694397	-2.179561	-0.594277
H	3.705156	3.524653	-2.014672
H	3.628030	2.014657	-2.952415
H	2.261125	2.486236	-1.931849
H	3.688085	3.621815	0.293144
H	2.399013	2.446301	0.589061
H	3.987756	2.258067	1.376535
H	5.773120	2.976916	-0.818968
H	6.011329	1.427362	0.006026
H	5.932634	1.493112	-1.766834
H	3.389308	-2.600220	2.062489

H	2.418631	-2.489714	0.590798
H	4.176012	-2.779487	0.488919
H	5.078117	-0.930542	2.623160
H	5.829534	-0.794426	1.025725
H	5.166511	0.645108	1.828120
H	2.618647	-0.676844	2.958366
H	2.672523	0.923973	2.190391
H	1.551590	-0.308228	1.588612
H	-0.064407	1.320950	2.954802
H	1.486103	2.878336	4.120009
H	2.002059	5.104053	3.124006
H	0.987405	5.735651	0.934550
H	-0.521865	4.154393	-0.242497
H	-0.355753	-3.990644	-0.549707
H	1.037997	-5.697893	0.605430
H	1.730869	-5.348609	2.974669
H	1.029597	-3.278088	4.169963
H	-0.390175	-1.591605	3.011173
H	-0.241886	0.086858	-1.798252
H	1.547970	0.102666	-3.070909

96

TS^{II}_{Cu}

C	-0.591913	-3.335198	-0.348131
C	0.147573	-2.450111	-1.142419
C	0.015076	-2.497120	-2.535012
C	-0.839849	-3.428179	-3.127680
C	-1.560401	-4.323055	-2.334597
C	-1.440008	-4.268891	-0.944222
N	1.080584	-1.555211	-0.533028
AL	0.829884	0.187912	0.133010
N	1.477807	1.841491	-0.518540
C	0.722477	2.899896	-1.090650
C	0.187363	2.768217	-2.377067
C	-0.590869	3.788540	-2.927010
C	-0.826726	4.958720	-2.205893
C	-0.278819	5.103714	-0.927887
C	0.487949	4.082734	-0.372328
C	2.339746	-2.091192	-0.261846
C	3.232972	-1.267812	0.431336
C	4.576422	-1.484580	0.658815
C	5.063901	-2.736084	0.249631
C	4.194540	-3.646622	-0.362219
C	2.855689	-3.347178	-0.633404
O	2.666413	-0.020398	0.791575
C	3.502569	1.068124	0.444340
C	4.861445	0.965062	0.666615
C	5.379936	-0.338710	1.293530
C	5.625992	2.067910	0.254585
C	4.992503	3.154612	-0.359841
C	3.620289	3.176183	-0.626682
C	2.823722	2.080100	-0.244075
C	6.882251	-0.513406	1.068218
C	5.092668	-0.310661	2.815822
CU	-1.371901	-0.567838	1.437375
P	-3.336997	-0.030617	0.500749
C	-3.683133	1.743830	1.171050
C	-2.900622	2.764248	0.330111
C	-3.558854	-0.009101	-1.411384
C	-3.681088	-1.447869	-1.932153
C	-4.658274	-1.231167	1.231492
C	-4.735203	-1.007115	2.751067
C	-4.751499	0.808533	-1.928311
C	-2.262891	0.548207	-2.015860
C	-6.067503	-1.119774	0.635854
C	-4.126403	-2.666079	1.051348
C	-5.157102	2.165647	1.221803
C	-3.079410	1.823181	2.588350
H	3.173866	4.016243	-1.156328
H	6.704955	2.076715	0.395611
H	6.109957	-2.997608	0.396405
H	2.222734	-4.064046	-1.153922
H	7.230101	-1.444875	1.532030
H	7.436020	0.310203	1.535941
H	7.130475	-0.540525	-0.000554
H	5.422578	-1.252016	3.275411
H	4.022646	-0.183645	3.020205
H	5.636381	0.521471	3.282981
H	5.595855	4.007916	-0.672316
H	4.580459	-4.620970	-0.664717
H	-4.792640	-3.350797	1.599079
H	-4.096780	-2.991529	0.008812
H	-3.116632	-2.753671	1.476078
H	-5.338972	-1.819812	3.183433
H	-3.735316	-1.051171	3.204101
H	-5.218022	-0.061907	3.020888
H	-6.728138	-1.819056	1.172591
H	-6.493282	-0.116141	0.743830
H	-6.099503	-1.395571	-0.423817
H	-3.168654	2.861759	2.944370
H	-3.578320	1.174651	3.311935
H	-2.009934	1.563135	2.579352
H	-2.958584	3.740281	0.835510
H	-1.839016	2.499676	0.254867
H	-3.302149	2.897770	-0.679362
H	-5.209926	3.204548	1.584701
H	-5.638305	2.134221	0.238320
H	-5.743476	1.550981	1.913123
H	-3.589111	-1.422186	-3.028792
H	-2.876593	-2.085805	-1.551805
H	-4.644351	-1.909816	-1.693521
H	-4.801891	0.695220	-3.022859
H	-5.705422	0.462939	-1.515822
H	-4.652632	1.878739	-1.717684
H	-2.345463	0.530902	-3.113967
H	-2.045732	1.573168	-1.714112
H	-1.408112	-0.088439	-1.743150
H	0.387588	1.856114	-2.940542
H	-1.009294	3.667391	-3.926859
H	-1.432650	5.756215	-2.636415
H	-0.461329	6.013878	-0.355325
H	0.903685	4.183134	0.630761
H	-0.492209	-3.274897	0.737204
H	-2.012575	-4.953192	-0.317354
H	-2.223105	-5.053894	-2.798793
H	-0.937725	-3.458057	-4.213384
H	0.600293	-1.808339	-3.145605
H	-0.079271	0.409863	1.480013
H	-1.220971	-1.533937	2.648415

96

PC'_{Cu}

C	-2.726971	2.172453	0.390228
C	-3.504374	1.192930	-0.245439
C	-4.845099	1.269619	-0.570947
C	-5.468226	2.498576	-0.297926
C	-4.726772	3.538410	0.274348
C	-3.380766	3.397666	0.623989
C	-5.498968	0.039714	-1.214234
C	-4.879505	-1.207928	-0.570796
C	-3.535931	-1.170188	-0.249592
O	-2.793373	0.001980	-0.461723
C	-5.536659	-2.418724	-0.297432
C	-4.823251	-3.480560	0.270110
C	-3.471603	-3.380249	0.610908
C	-2.782613	-2.175235	0.374443
N	-1.493987	-1.817127	0.747158
AL	-0.930246	-0.025556	0.369894
CU	1.356951	0.005190	-0.216128
P	3.501189	0.026234	-0.627788
C	4.512915	-0.150515	0.999504
C	6.013145	0.145498	0.876755
C	-7.021346	0.061099	-1.058726
C	-5.143903	0.034135	-2.723386
N	-1.455466	1.771522	0.774988
C	-0.483982	2.748160	1.092940
C	0.297907	2.618292	2.252236
C	1.300846	3.543187	2.540990
C	1.538092	4.621404	1.686519
C	0.762856	4.760703	0.531937
C	-0.232373	3.833552	0.233592
C	-0.547459	-2.826640	1.034562
C	0.284927	-2.718842	2.161135
C	1.270068	-3.674049	2.411488

C	1.440096	-4.760647	1.551591
C	0.610172	-4.881121	0.433646
C	-0.368462	-3.925347	0.173922
C	3.865435	-1.474116	-1.768983
C	5.336908	-1.895857	-1.860005
C	4.012578	1.652844	-1.509221
C	5.360542	1.593644	-2.239476
C	3.008845	-2.649320	-1.257924
C	3.343652	-1.161240	-3.181339
C	2.891909	2.022311	-2.500442
C	4.049777	2.792728	-0.478604
C	3.882705	0.779590	2.055101
C	4.317424	-1.578548	1.534671
H	-2.846503	4.217061	1.102810
H	-6.521618	2.647245	-0.527174
H	-6.594777	-2.536612	-0.522737
H	-2.958915	-4.216387	1.084142
H	-7.467568	-0.815028	-1.545442
H	-7.442440	0.949674	-1.545224
H	-7.318730	0.065221	-0.002210
H	-5.573105	-0.853844	-3.207322
H	-4.056938	0.018748	-2.873326
H	-5.547831	0.933564	-3.207919
H	-5.220726	4.490335	0.474126
H	-5.343573	-4.417927	0.471825
H	3.096293	-3.480923	-1.974205
H	3.308045	-3.022461	-0.276264
H	1.946208	-2.369786	-1.200494
H	3.390503	-2.088885	-3.771952
H	2.296247	-0.832176	-3.155195
H	3.942144	-0.408061	-3.704188
H	5.419521	-2.729051	-2.575311
H	5.982935	-1.086233	-2.218568
H	5.729847	-2.251792	-0.901259
H	3.133777	3.001442	-2.942426
H	2.777186	1.304778	-3.316495
H	1.925759	2.109171	-1.984333
H	4.153416	3.741299	-1.027460
H	3.121368	2.851369	0.103522
H	4.898781	2.718194	0.208683
H	5.573280	2.587917	-2.662660
H	6.187776	1.335616	-1.568837
H	5.357005	0.880670	-3.071306
H	4.735135	-1.620556	2.552191
H	3.253645	-1.843031	1.600325
H	4.835635	-2.335176	0.936397
H	6.489454	-0.046095	1.851065
H	6.508989	-0.490848	0.135365
H	6.211427	1.192187	0.619845
H	4.347058	0.555434	3.027866
H	4.036723	1.841232	1.849346
H	2.801890	0.602244	2.138151
H	0.107710	1.780673	2.922667
H	1.897272	3.421591	3.446292
H	2.320973	5.345032	1.914852
H	0.943803	5.592071	-0.150942
H	-0.823519	3.935670	-0.677035
H	-0.999648	-4.013330	-0.710760
H	0.734984	-5.720406	-0.252133
H	2.209634	-5.506907	1.750437
H	1.905015	-3.570453	3.292513
H	0.150533	-1.874350	2.836094
H	-0.016909	-0.008446	-1.154478
H	0.583296	-0.042392	1.251328

96

TS^{IV}_{Cu}

C	-0.275335	2.394565	1.944403
C	0.548542	2.724032	0.851706
C	0.343195	3.956970	0.209963
C	-0.650508	4.830487	0.651525
C	-1.462106	4.495617	1.737528
C	-1.266589	3.271571	2.381629
N	1.508315	1.792381	0.407053
AL	0.989818	0.005568	-0.037498
CU	-1.458861	-0.013720	0.097199
P	-3.694542	-0.013838	-0.244124
C	-4.018512	1.416581	-1.482875
C	-3.215756	2.639261	-0.993794
C	2.815223	2.185965	0.149085
C	3.638729	1.189939	-0.401746
C	5.004025	1.249199	-0.603046
C	5.615144	2.472833	-0.282018
C	4.838435	3.521729	0.220338
C	3.463422	3.399872	0.446369
O	2.944817	0.002996	-0.654482
C	3.644288	-1.174862	-0.375561
C	5.009982	-1.231961	-0.575227
C	5.700419	0.003856	-1.169155
C	5.626788	-2.445426	-0.227553
C	4.854838	-3.486847	0.297087
C	3.479088	-3.366963	0.519524
C	2.825085	-2.162837	0.196076
N	1.516133	-1.769598	0.444817
C	0.559350	-2.695170	0.907752
C	-0.272402	-2.343796	1.988155
C	-1.258043	-3.217149	2.445303
C	-1.437815	-4.460978	1.835196
C	-0.622518	-4.815544	0.758076
C	0.364537	-3.945195	0.296011
C	7.199876	0.011449	-0.865078
C	5.493244	-0.014060	-2.705941
C	-4.166315	-1.687599	-1.059742
C	-2.996947	-2.098867	-1.978086
C	-4.756193	0.247481	1.331079
C	-4.100052	-0.581814	2.453009
C	-4.237454	-2.774429	0.024657
C	-5.479321	-1.682311	-1.852172
C	-4.646868	1.715123	1.773410
C	-6.239227	-0.120402	1.197326
C	-3.413567	1.035163	-2.844691
C	-5.490395	1.800580	-1.677900
H	2.924218	-4.187484	0.971049
H	6.698617	-2.579744	-0.360271
H	6.686327	2.609068	-0.417887
H	2.904599	4.226964	0.880716
H	7.677899	0.889071	-1.317769
H	7.682373	-0.875642	-1.293880
H	7.390877	0.026540	0.215679
H	5.952237	0.876339	-3.156935
H	4.425814	-0.020833	-2.960483
H	5.958760	-0.911033	-3.136785
H	5.344396	-4.425575	0.560005
H	5.323534	4.468381	0.462238
H	-3.244968	3.409146	-1.780053
H	-3.609449	3.085057	-0.078156
H	-2.161120	2.381924	-0.814063
H	-3.449259	1.924402	-3.492131
H	-2.360678	0.735732	-2.750778
H	-3.965702	0.237225	-3.351472
H	-5.546868	2.607679	-2.425278
H	-6.096338	0.964868	-2.045698
H	-5.949185	2.178419	-0.757480
H	-5.090354	1.801092	2.777189
H	-3.602045	2.043580	1.839604
H	-5.191234	2.399976	1.115003
H	-4.614857	-0.357737	3.399988
H	-4.156225	-1.660294	2.286840
H	-3.040693	-0.311496	2.569086
H	-6.746667	0.111286	2.147117
H	-6.737548	0.452884	0.407015
H	-6.393070	-1.186257	0.997821
H	-5.666054	-2.700460	-2.228497
H	-6.338043	-1.394310	-1.234863
H	-5.441538	-1.016759	-2.721590
H	-3.207266	-3.104845	-2.373456
H	-2.851193	-1.429520	-2.829209
H	-2.054100	-2.149616	-1.413507
H	-4.337747	-3.747493	-0.479968
H	-3.318164	-2.811843	0.622191
H	-5.097751	-2.662497	0.692455
H	-0.108694	-1.386091	2.484397
H	-1.882265	-2.926096	3.291140
H	-2.206825	-5.145221	2.194169

H	-0.760377	-5.778136	0.263537
H	0.983648	-4.223066	-0.556958
H	0.957540	4.217214	-0.651999
H	-0.796746	5.779767	0.134068
H	-2.236109	5.182395	2.080751
H	-1.878053	3.002708	3.243993
H	-0.101764	1.451554	2.464833
H	-0.244442	-0.010069	-1.189375
H	0.631226	-0.013718	-1.930346

96

PC$_{Cu}$

C	0.643805	-2.574145	-3.069890
C	0.096339	-2.783838	-1.785350
C	0.627286	-3.844454	-1.021258
C	1.645555	-4.652014	-1.523584
C	2.179984	-4.427246	-2.793385
C	1.671261	-3.375738	-3.559572
N	-0.883760	-1.887054	-1.329386
AL	-0.861271	0.010292	-1.921236
N	-0.946220	1.893690	-1.295059
C	0.000019	2.828736	-1.745723
C	0.527297	3.872697	-0.957557
C	1.513784	4.721337	-1.456147
C	2.017920	4.554449	-2.747258
C	1.511910	3.519837	-3.537829
C	0.517383	2.676289	-3.050466
C	-1.905142	-2.275252	-0.477713
C	-2.773680	-1.236235	-0.084625
C	-3.836483	-1.317270	0.792500
C	-4.099865	-2.595310	1.315611
C	-3.328100	-3.682666	0.903389
C	-2.252381	-3.548133	0.021928
O	-2.448730	-0.025412	-0.702594
C	-2.802554	1.159038	-0.049639
C	-3.865104	1.189095	0.830693
C	-4.695941	-0.077508	1.059277
C	-4.157305	2.444953	1.391383
C	-3.410313	3.561491	1.012803
C	-2.334310	3.478843	0.125081
C	-1.963435	2.230870	-0.418185
C	-5.272040	-0.105538	2.481640
C	-5.860803	-0.075419	0.036211
CU	1.404445	0.096771	-0.595413
P	2.283053	0.034703	1.413739
C	0.948568	-0.618154	2.627120
C	0.726747	-2.110965	2.340591
C	3.786488	-1.154965	1.371023
C	4.958180	-0.458215	0.659219
C	2.855376	1.777185	1.957759
C	1.616202	2.613717	2.313792
C	4.251575	-1.649812	2.746604
C	3.410329	-2.355114	0.484647
C	3.824875	1.790630	3.146550
C	3.504274	2.460272	0.738340
C	1.271247	-0.425798	4.113483
C	-0.383351	0.075234	2.280224
H	-1.803013	4.383679	-0.155925
H	-4.979590	2.557541	2.095010
H	-4.919861	-2.747358	2.014452
H	-1.699912	-4.431599	-0.284783
H	-5.894726	-0.995868	2.629446
H	-5.914411	0.765873	2.655524
H	-4.472873	-0.107466	3.234089
H	-6.473447	-0.978869	0.160054
H	-5.477260	-0.055327	-0.992114
H	-6.494537	0.809104	0.187448
H	-3.673575	4.538364	1.421001
H	-3.570846	-4.677737	1.279165
H	3.718747	3.506584	1.004358
H	4.443976	1.996261	0.430538
H	2.821632	2.473847	-0.122072
H	1.932584	3.662539	2.415403
H	0.858474	2.570376	1.522697
H	1.158289	2.314030	3.261813
H	4.038526	2.838328	3.407982
H	3.404584	1.306027	4.035104
H	4.782276	1.312714	2.912678
H	-1.192622	-0.430433	2.827175
H	-0.413908	1.135248	2.539260
H	-0.600397	-0.020526	1.208030
H	-0.163070	-2.437599	2.898859
H	0.527311	-2.283877	1.276231
H	1.566483	-2.738003	2.657124
H	0.460945	-0.878947	4.705075
H	2.207451	-0.914151	4.405880
H	1.328458	0.630634	4.397740
H	5.743175	-1.210965	0.492222
H	4.661490	-0.066563	-0.323119
H	5.400065	0.352348	1.247045
H	5.142082	-2.281650	2.606120
H	4.529147	-0.829757	3.417958
H	3.493339	-2.264129	3.244980
H	4.305189	-2.982494	0.353649
H	2.624183	-2.981980	0.908284
H	3.077196	-2.034274	-0.512841
H	0.188417	4.003885	0.066316
H	1.903770	5.514933	-0.816633
H	2.790708	5.220448	-3.131436
H	1.885665	3.372656	-4.552247
H	0.109569	1.891646	-3.688437
H	0.233334	-1.778833	-3.692732
H	2.067928	-3.182883	-4.557488
H	2.977295	-5.062028	-3.180095
H	2.036526	-5.459856	-0.902764
H	0.267094	-4.021339	-0.011152
H	-1.331193	0.014419	-3.456661
H	0.916430	0.053411	-2.071418

96

PC$_{Cu}$-HH

C	1.851539	2.086863	1.035284
C	2.922672	1.577770	0.285335
C	3.541595	2.173588	-0.796797
C	3.118632	3.475592	-1.103062
C	2.106257	4.067996	-0.341506
C	1.464213	3.399992	0.703141
C	4.574359	1.345088	-1.571314
C	4.002777	-0.075769	-1.666139
C	3.369119	-0.586145	-0.549554
O	3.247949	0.231990	0.607177
C	4.014199	-0.907964	-2.795815
C	3.378329	-2.153290	-2.741955
C	2.698073	-2.600522	-1.607311
C	2.658001	-1.791486	-0.455494
N	1.935004	-1.970787	0.715832
AL	2.013489	-0.542408	1.941428
CU	-2.896725	-0.956577	0.716855
P	-2.936021	0.215553	-1.152680
C	-1.307703	0.070030	-2.154936
C	-1.128773	1.105722	-3.272440
C	4.849045	1.935696	-2.954517
C	5.897083	1.305845	-0.764975
N	1.263334	1.189790	1.918186
C	0.231461	1.573648	2.798546
C	-0.852747	0.700417	2.979023
C	-1.883341	1.005336	3.864936
C	-1.856673	2.199517	4.586150
C	-0.777663	3.073092	4.419926
C	0.261576	2.766245	3.543527
C	1.295325	-3.196918	1.003517
C	-0.015728	-3.179689	1.500070
C	-0.661435	-4.362270	1.856542
C	-0.011709	-5.588177	1.712667
C	1.294796	-5.615325	1.213829
C	1.947701	-4.434765	0.866521
C	-4.389323	-0.460799	-2.211609
C	-4.394529	-0.024757	-3.681819
C	-3.256244	2.062340	-0.725972
C	-3.785224	2.919747	-1.882239
C	-4.345224	-2.000353	-2.126403
C	-5.716573	-0.044255	-1.555795
C	-4.249311	2.095207	0.453493
C	-1.959675	2.690401	-0.190252

C	-0.141780	0.183831	-1.159531
C	-1.205885	-1.343837	-2.751144
H	0.637538	3.873760	1.227584
H	3.560946	4.018034	-1.936125
H	4.498684	-0.584002	-3.714621
H	2.166124	-3.549721	-1.623411
H	5.593421	1.329649	-3.485711
H	5.259413	2.948862	-2.861464
H	3.936856	1.982326	-3.563321
H	6.635624	0.684627	-1.289123
H	5.744010	0.889107	0.237921
H	6.299651	2.322336	-0.659889
H	1.783738	5.080592	-0.587153
H	3.389765	-2.790943	-3.626777
H	-5.250447	-2.398064	-2.610778
H	-3.478753	-2.438951	-2.627018
H	-4.342825	-2.337672	-1.079265
H	-6.530258	-0.583684	-2.063903
H	-5.745515	-0.322066	-0.493330
H	-5.923383	1.026856	-1.648916
H	-5.294510	-0.434434	-4.166651
H	-4.423098	1.064847	-3.796051
H	-3.526914	-0.406580	-4.231614
H	-4.358189	3.140264	0.783083
H	-5.243443	1.719793	0.199169
H	-3.866945	1.511593	1.303702
H	-2.208681	3.683030	0.215534
H	-1.531001	2.100787	0.629688
H	-1.196640	2.835517	-0.961522
H	-3.889518	3.957733	-1.529184
H	-3.103723	2.928887	-2.740413
H	-4.772026	2.594193	-2.229624
H	-0.184611	-1.472814	-3.140803
H	-1.365230	-2.115784	-1.985894
H	-1.901423	-1.512014	-3.579942
H	-0.185114	0.890320	-3.797786
H	-1.936303	1.074433	-4.011944
H	-1.055610	2.127428	-2.883189
H	0.795813	-0.004058	-1.703748
H	-0.060125	1.163885	-0.688052
H	-0.226862	-0.574841	-0.370225
H	-0.921867	-0.216313	2.386129
H	-2.715129	0.306561	3.962628
H	-2.665505	2.448520	5.273148
H	-0.736650	4.002172	4.990477
H	1.112150	3.440433	3.449181
H	2.975773	-4.462640	0.505120
H	1.817481	-6.566750	1.105773
H	-0.515881	-6.515790	1.984435
H	-1.685365	-4.309909	2.228842
H	-0.564034	-2.237348	1.579349
H	-2.965381	-1.765000	2.002953
H	2.330094	-1.031027	3.413957

102

RC^{cat}$_{Cu}$

C	2.113204732	-2.289863976	-0.445473815
C	2.972047522	-1.180685709	-0.289277084
C	4.167096902	-1.135158653	0.399457667
C	4.590187513	-2.352047274	0.964238394
C	3.827129722	-3.505038026	0.778584939
C	2.611385673	-3.496356153	0.088865390
C	4.990373193	0.156423593	0.445167090
C	4.106190900	1.367118802	0.129062826
C	2.915141171	1.207713970	-0.549180183
O	2.475689660	-0.055001492	-0.947217481
C	4.466250491	2.695412692	0.419876021
C	3.649756152	3.744595756	-0.003109449
C	2.440798229	3.530993418	-0.670769312
C	2.005273133	2.215727820	-0.934248415
N	0.858795306	1.751569645	-1.557744762
AL	0.748317653	-0.204019764	-1.927726296
CU	-1.287671198	-0.075021918	-0.273403349
P	-1.847846838	0.178544635	1.827259421
C	-2.349294659	-1.511053786	2.571447807
C	-3.170397006	-2.263993023	1.506479515
C	5.645794101	0.321224796	1.825899695
C	6.092537742	0.067098855	-0.639825183
N	0.950700502	-2.026459613	-1.150266101
C	-0.065933171	-2.979897310	-1.305556995
C	-0.391135099	-3.961447617	-0.344872720
C	-1.459916595	-4.833105454	-0.539326496
C	-2.251864812	-4.753009313	-1.686270534
C	-1.950325885	-3.780077755	-2.641952380
C	-0.874880051	-2.915163172	-2.461620818
C	-0.199007143	2.599177063	-1.919109378
C	-0.990314544	2.239171738	-3.032525269
C	-2.105922715	2.985477933	-3.400839546
C	-2.465885109	4.129479554	-2.684733174
C	-1.689979444	4.504061440	-1.586026999
C	-0.582470062	3.751969785	-1.200354229
C	-0.337725414	0.891147077	2.772792505
C	0.920987191	0.146017199	2.287713699
C	-3.328871228	1.391755373	1.927963746
C	-3.080462697	2.520871778	0.913027511
C	-0.146912321	2.354638048	2.343988088
C	-0.435354912	0.813999964	4.301604939
C	-4.603599719	0.673584850	1.454653718
C	-3.570878804	1.987608058	3.320502657
C	-1.077777350	-2.341568256	2.802637381
C	-3.135225403	-1.428839926	3.886598154
H	2.069167557	-4.427464888	-0.049287626
H	5.524727678	-2.405858238	1.519564195
H	5.393789324	2.911666734	0.946683370
H	1.853311988	4.383573839	-0.999387422
H	6.261065368	1.228220707	1.858418751
H	6.307001531	-0.525306258	2.045727180
H	4.886360047	0.385300633	2.616028987
H	6.701056779	0.981694768	-0.637588541
H	5.648085584	-0.050866568	-1.636512030
H	6.745493996	-0.794967845	-0.446835519
H	4.188733472	-4.451553758	1.182923292
H	3.963291089	4.771317714	0.190651074
H	-3.367385778	-3.280555855	1.879376013
H	-4.133602006	-1.796590377	1.289008821
H	-2.607971695	-2.364440465	0.567976931
H	-1.382376288	-3.375264213	3.024239364
H	-0.445494819	-2.368483795	1.907478300
H	-0.484757082	-1.982152722	3.649522976
H	-3.313877014	-2.453572692	4.247134412
H	-2.584652910	-0.893912000	4.668747823
H	-4.113689054	-0.951990976	3.764658122
H	1.806243928	0.674823479	2.670962218
H	0.978849520	-0.891717421	2.622000738
H	0.979654296	0.155800480	1.191295907
H	0.820577298	2.700977288	2.736281914
H	-0.110379129	2.448032363	1.251421726
H	-0.921986273	3.020517637	2.736819780
H	0.457991233	1.293563657	4.730191350
H	-1.313160557	1.339010905	4.693878324
H	-0.457145305	-0.218397491	4.668066029
H	-5.398855199	1.428656248	1.365324602
H	-4.469979414	0.217856173	0.464984209
H	-4.954197410	-0.092112509	2.153404557
H	-4.467758324	2.624403411	3.275927612
H	-3.746495077	1.219967954	4.082368741
H	-2.739866908	2.620354632	3.651252203
H	-3.982452970	3.149774882	0.866333164
H	-2.239155873	3.165064799	1.173371632
H	-2.894663356	2.126316184	-0.095901932
H	0.175405744	-4.023634401	0.580067291
H	-1.684128429	-5.575808604	0.228251897
H	-3.087315317	-5.437566606	-1.833434817
H	-2.551333985	-3.698449657	-3.548914191
H	-0.637573492	-2.181219296	-3.232103549
H	-0.706473085	1.366573499	-3.621107852
H	-2.692698997	2.672631543	-4.265925407
H	-3.333598319	4.719917730	-2.979369220
H	-1.959952944	5.386507273	-1.003452508
H	-0.032091574	4.046252209	-0.310267189
H	0.977571009	-0.369163144	-3.509811964
H	-1.010308878	-0.235511482	-1.790518541
C	-3.901804116	-0.349206242	-2.443232939
C	-4.106865784	-0.599388955	-3.735010182

H	-3.819722409	0.672021832	-2.067357050
H	-3.786908686	-1.157393869	-1.718432182
H	-4.173555989	-1.619524154	-4.116463708
H	-4.207161399	0.205796800	-4.464676196

102

TS^{cat}_{Cu}

C	-2.005407927	-2.288897055	0.291933626
C	-2.745031206	-1.148128637	-0.082344715
C	-3.749912401	-1.082712847	-1.025722773
C	-4.066606641	-2.301396952	-1.656169939
C	-3.382382254	-3.470045874	-1.312666662
C	-2.363653469	-3.486148485	-0.355051272
C	-4.490727687	0.231190308	-1.296175113
C	-3.706943221	1.419133204	-0.725842245
C	-2.700072450	1.227757490	0.199156356
O	-2.303799110	-0.034038932	0.619305894
C	-3.991357536	2.761831486	-1.040147704
C	-3.282933813	3.793970355	-0.420927810
C	-2.265591569	3.550848915	0.507085205
C	-1.928980947	2.224081964	0.833610304
N	-0.975060322	1.738616358	1.705785028
AL	-0.556578276	-0.198913392	1.738443462
CU	1.464285487	-0.148611938	0.274601032
P	2.092633403	0.189186674	-1.840203153
C	2.685446702	-1.444874636	-2.647312446
C	3.467491283	-2.232159288	-1.576945878
C	-4.713277000	0.414649370	-2.808427231
C	-5.864923481	0.169827372	-0.585615267
N	-1.047950346	-2.057244851	1.256890182
C	-0.158662076	-3.093700359	1.620233806
C	0.690553305	-3.701727192	0.679609039
C	1.629714314	-4.652014445	1.073015341
C	1.741939686	-5.016450998	2.417716596
C	0.896156064	-4.426202114	3.358999152
C	-0.049114789	-3.478924578	2.964547534
C	-0.077725794	2.626917139	2.338100346
C	0.099467807	2.551490350	3.729222434
C	1.048179237	3.345470129	4.372973142
C	1.828884129	4.245705456	3.645485364
C	1.649359992	4.340978012	2.262644510
C	0.709058104	3.542160094	1.616869810
C	0.621846354	0.904450453	-2.837891177
C	-0.625044428	0.072441312	-2.484799721
C	3.540440259	1.448572736	-1.801146980
C	3.201244252	2.516091315	-0.743335972
C	0.339457249	2.324041512	-2.318794691
C	0.809290618	0.941445626	-4.358883493
C	4.802684636	0.737638118	-1.285080399
C	3.848904110	2.129029376	-3.140236953
C	1.458654747	-2.300899660	-3.003148098
C	3.544339945	-1.273040407	-3.906885492
H	-1.848129005	-4.413677617	-0.110537210
H	-4.854198512	-2.340402655	-2.406997179
H	-4.776854929	3.002030053	-1.754885614
H	-1.735350364	4.379742664	0.972847830
H	-5.257973219	1.344629027	-3.011029035
H	-5.310993927	-0.410241520	-3.214637864
H	-3.757266096	0.446515626	-3.346372453
H	-6.423976523	1.099601091	-0.758041651
H	-5.735296963	0.038600056	0.496562020
H	-6.453542635	-0.674660345	-0.969245584
H	-3.652447119	-4.404809350	-1.806249638
H	-3.532660293	4.827423590	-0.665643917
H	3.694953428	-3.231466433	-1.979120295
H	4.414432726	-1.762859815	-1.299755644
H	2.870243296	-2.365352930	-0.663893330
H	1.810895955	-3.312752943	-3.253313443
H	0.758645106	-2.388242631	-2.163237452
H	0.913596195	-1.918194868	-3.872042681
H	3.777785210	-2.270871181	-4.309884164
H	3.023797342	-0.712485215	-4.691775990
H	4.497689450	-0.774418064	-3.701475638
H	-1.517450094	0.633188523	-2.796458902
H	-0.656850298	-0.904288565	-2.971819646
H	-0.695104063	-0.085449819	-1.400418231
H	-0.613960582	2.663756351	-2.749367478
H	0.222375593	2.332578191	-1.226979912
H	1.110368112	3.045983371	-2.607271464
H	-0.084026763	1.401568794	-4.809107547
H	1.677262432	1.536591063	-4.662990690
H	0.909701692	-0.061223184	-4.789524297
H	5.571012105	1.504021341	-1.102897006
H	4.615905526	0.223534240	-0.332294795
H	5.217613800	0.020601290	-2.000218435
H	4.723799786	2.784507218	-3.008115078
H	4.088183781	1.410559433	-3.932254126
H	3.020363502	2.759061530	-3.483212111
H	4.085097596	3.156804137	-0.601664142
H	2.367394793	3.159874411	-1.030989590
H	2.956004805	2.060363840	0.227025904
H	0.607191253	-3.410621913	-0.366327506
H	2.286602815	-5.102887682	0.327258867
H	2.481119720	-5.755452576	2.728232828
H	0.969910137	-4.707597037	4.410422726
H	-0.712099235	-3.014778550	3.694602736
H	-0.516912481	1.852953063	4.294349063
H	1.172004796	3.264855747	5.454066091
H	2.568833288	4.867946975	4.149607234
H	2.255901488	5.035825438	1.679338718
H	0.586111384	3.609269686	0.536914037
H	0.100288470	-0.412469696	3.313001955
H	1.635035427	-0.391305557	1.862703199
C	2.515313366	-0.648436979	3.085871394
C	1.536494658	-0.641240536	4.084422340
H	3.164533936	0.220053966	2.965292118
H	2.937856362	-1.599969824	2.758859659
H	1.189543467	-1.578249880	4.515902632
H	1.389340168	0.247588807	4.696442124

102

PC^{cat}_{Cu}

C	0.521393015	3.598232807	-1.644961832
C	0.692722908	2.242322893	-1.988567009
C	-0.122985628	1.709530049	-3.005714457
C	-1.066392708	2.500536482	-3.659419251
C	-1.217159404	3.845710937	-3.320561346
C	-0.417586564	4.384487935	-2.307391857
N	1.553510258	1.378233108	-1.298379016
AL	0.796640777	-0.222358258	-0.495106245
CU	-1.413122416	-0.045132093	0.252203770
P	-3.566744814	0.087306055	0.838133433
C	-4.468046513	-1.422229122	0.069876634
C	-4.547028199	-1.216320795	-1.452713075
O	2.561980900	-0.147920799	0.501334623
C	3.364574597	0.961429763	0.153539944
C	4.641949088	1.049133471	0.673272246
C	5.097947633	-0.080877167	1.606891600
C	4.558880036	-1.387478986	1.005542490
C	3.282636703	-1.362298935	0.474687241
C	5.397074644	2.158225618	0.262930578
C	4.842328473	3.069587808	-0.643681473
C	3.567156441	2.904826521	-1.189086387
C	2.778453893	1.799911129	-0.806639876
C	2.634375874	-2.386764279	-0.235008459
C	3.351767436	-3.599443761	-0.306826969
C	4.622918201	-3.690426593	0.265350113
C	5.241481905	-2.608882158	0.903372406
C	6.619010361	-0.112019331	1.758522397
C	4.454845698	0.135728428	3.000201596
N	1.436791488	-2.028381986	-0.834146787
C	0.506097859	-2.964154075	-1.298680532
C	0.273477321	-4.212625795	-0.686915562
C	-0.742841515	-5.048500864	-1.143046205
C	-1.562400379	-4.668838374	-2.210549761
C	-1.352274832	-3.428855092	-2.815261021
C	-0.330355233	-2.592030360	-2.371564700
C	-3.774553701	0.061974799	2.744991315
C	-3.483218158	-1.358750464	3.255638973
C	-4.303354342	1.713587153	0.135257841
C	-3.803075409	2.891616845	0.988419636
C	-2.664510082	0.954010747	3.337540551
C	-5.145714485	0.509799479	3.265698919
C	-3.687072331	1.925825571	-1.261587751

C	-5.833319087	1.765309382	0.049573538
C	-3.562790671	-2.654587889	0.275306994
C	-5.872249927	-1.704800951	0.616480545
H	3.195183521	3.607769550	-1.932314491
H	6.411297842	2.304744208	0.629463905
H	6.249882935	-2.714886289	1.298679505
H	2.935869112	-4.447826306	-0.846941792
H	6.918116324	-0.921549579	2.436139562
H	6.977214431	0.828644396	2.195210680
H	7.118446606	-0.261955881	0.792727141
H	4.738278962	-0.683093600	3.675225871
H	3.360580426	0.167940254	2.934392066
H	4.802981515	1.085436562	3.428515627
H	5.436974647	3.927933991	-0.959601610
H	5.162900635	-4.635050607	0.185723367
H	-4.077135255	3.823540212	0.471051471
H	-4.255302739	2.923379407	1.984906826
H	-2.709491205	2.878428381	1.090224895
H	-3.968862123	2.928304985	-1.618369072
H	-2.589169341	1.883144697	-1.225386204
H	-4.028869149	1.200304161	-2.003736505
H	-6.131990160	2.755501098	-0.329600535
H	-6.236252700	1.017479323	-0.642830669
H	-6.312942073	1.624259393	1.025058292
H	-2.657817548	0.821420099	4.430454271
H	-1.677482804	0.661178181	2.950801050
H	-2.805846299	2.017722056	3.132250294
H	-3.421705985	-1.318833154	4.353782889
H	-4.269817139	-2.074777469	2.996204192
H	-2.521770162	-1.733840498	2.879946510
H	-5.157670503	0.410949331	4.362680676
H	-5.359537839	1.558495404	3.031056132
H	-5.962096290	-0.104376230	2.868555734
H	-3.979682620	-3.490602711	-0.306549041
H	-2.544735612	-2.470275380	-0.097750460
H	-3.495040219	-2.975049799	1.317468339
H	-6.298103878	-2.561175282	0.070074503
H	-5.858990066	-1.972170212	1.679317756
H	-6.551982847	-0.855218259	0.484215875
H	-4.890095467	-2.159216922	-1.904914880
H	-5.255356190	-0.433370548	-1.742784260
H	-3.560567160	-0.985435158	-1.877400310
H	0.874601968	-4.510826421	0.171047369
H	-0.905625662	-6.006674427	-0.646685553
H	-2.355480867	-5.329156199	-2.561544928
H	-1.982445457	-3.110697481	-3.647000355
H	-0.151756340	-1.637327224	-2.869659908
H	0.010003077	0.664939413	-3.292372557
H	-1.683557650	2.061262311	-4.444336435
H	-1.950542009	4.466958310	-3.834876113
H	-0.535855165	5.430281683	-2.018935823
H	1.113962755	4.023041232	-0.835325017
C	0.783098977	2.589604657	2.263530921
C	1.788229375	3.738419404	2.239155169
H	-0.053595567	2.797691658	2.945018478
H	2.629477076	3.516603476	1.569034487
H	2.200490590	3.931416145	3.239706116
H	1.252657111	1.652581541	2.594800673
H	1.320493786	4.670174749	1.890043073
H	0.356616415	2.399196064	1.267978823

96

RC_{Ag}

C	0.155576701	-2.391706243	-2.408726688
C	1.027756747	-2.766590261	-1.367837966
C	0.909322890	-4.069426795	-0.844975062
C	-0.035385244	-4.955767144	-1.355776945
C	-0.895456095	-4.571594556	-2.389624773
C	-0.795064662	-3.280071326	-2.908055775
N	1.885402350	-1.794789257	-0.835695315
AL	1.129846061	-0.090004424	-0.304851885
N	1.714116215	1.635007469	-0.980773010
C	0.766393555	2.467079011	-1.589606245
C	-0.078622541	1.913201558	-2.572922220
C	-1.103240166	2.662591934	-3.148581510
C	-1.308673425	3.988871712	-2.767552466
C	-0.481389686	4.548432147	-1.788718860
C	0.536364273	3.802009474	-1.200852967
C	3.136822184	-2.105969776	-0.326751807
C	3.739294255	-1.102890034	0.448729380
C	5.041787090	-1.073835648	0.911252499
C	5.809515671	-2.220485467	0.659766100
C	5.242103726	-3.285898155	-0.048518163
C	3.939067434	-3.244700555	-0.549379597
O	2.927130774	0.041673768	0.625315584
C	3.623594533	1.240394401	0.346716862
C	4.921248826	1.378253514	0.803872639
C	5.514607622	0.210907150	1.605077853
C	5.575890968	2.566831511	0.447634098
C	4.911606466	3.504962139	-0.350744220
C	3.620217960	3.294079299	-0.839811033
C	2.929076429	2.109396271	-0.510138670
C	7.039995462	0.289482364	1.671127470
C	4.942937295	0.244625592	3.045191672
AG	-1.224612083	-0.101070366	0.408999334
P	-3.636804594	0.008535868	0.868357961
C	-3.969432728	0.251351059	2.742105355
C	-2.981267951	-0.652349558	3.507069396
C	-4.355523095	1.494562304	-0.108313579
C	-3.368741458	2.670957962	0.034472393
C	-4.463072359	-1.617717724	0.273008891
C	-3.791888974	-2.024918134	-1.054376886
C	-4.361321996	1.138893555	-1.604362582
C	-5.763464885	1.937776648	0.309539547
C	-4.117147587	-2.738123749	1.268231729
C	-5.984943371	-1.557322240	0.091882140
C	-3.599011803	1.693194425	3.127777638
C	-5.404554711	-0.036108112	3.201655951
H	3.165680730	4.022015710	-1.509063676
H	6.597963571	2.754176594	0.771069781
H	6.843474038	-2.278966951	0.994338749
H	3.558497112	-4.070256577	-1.147675658
H	7.440313402	-0.551390293	2.251395532
H	7.352312731	1.212380464	2.175824346
H	7.489348888	0.268811777	0.669958155
H	5.333745873	-0.604711079	3.621840756
H	3.847716974	0.186736825	3.042507308
H	5.240214578	1.177406245	3.543407728
H	5.429928460	4.424096359	-0.627728269
H	5.847491730	-4.172310529	-0.243410081
H	-3.062765695	-0.423360086	4.580812351
H	-3.177328449	-1.719474762	3.378449021
H	-1.945751198	-0.454555058	3.194719789
H	-3.616863428	1.766026201	4.225771421
H	-2.585932064	1.953691618	2.793371991
H	-4.303892488	2.434389287	2.737949971
H	-5.480646125	0.180207312	4.278978523
H	-6.140305435	0.590258972	2.684544501
H	-5.685972311	-1.085720247	3.060500897
H	-3.696326882	3.478906844	-0.636907278
H	-3.319146325	3.077898794	1.046900248
H	-2.353901391	2.381480817	-0.272893737
H	-4.595479018	2.054196046	-2.168532682
H	-3.374560313	0.790645512	-1.938130732
H	-5.115604180	0.388518555	-1.862780278
H	-6.084604278	2.759346961	-0.350083099
H	-6.500366565	1.131601654	0.219043276
H	-5.793066062	2.317184880	1.337268608
H	-4.437783262	-3.693838942	0.826920524
H	-3.034562972	-2.801305504	1.443272836
H	-4.629380497	-2.631081838	2.229815464
H	-6.345322804	-2.556308853	-0.200248671
H	-6.505451299	-1.272948183	1.013420916
H	-6.281725906	-0.860383531	-0.700066356
H	-4.156417740	-3.026008529	-1.331583496
H	-4.016147893	-1.348054400	-1.882215718
H	-2.699543687	-2.085994624	-0.949060135
H	0.096938054	0.887100116	-2.899825873
H	-1.738377148	2.206252161	-3.909191087
H	-2.104766998	4.579030824	-3.221646371
H	-0.640298485	5.578647179	-1.465873712
H	1.143428526	4.241148454	-0.410446173
H	1.544181412	-4.373626646	-0.013574708
H	-0.109690410	-5.957823676	-0.930056074

H	-1.633419527	-5.270647006	-2.783381312
H	-1.456422938	-2.959695724	-3.714327889
H	0.248768603	-1.392996402	-2.838294209
H	0.151465206	2.886474829	2.159788493
H	0.044323227	2.278915554	1.722601288

96

TS^{I}_{Ag}

C	0.386356888	3.883128168	0.932340558
C	0.678594775	2.621511194	1.482511710
C	0.025940027	2.241983278	2.666763111
C	-0.886891936	3.096185020	3.285796842
C	-1.161037394	4.350065427	2.739696519
C	-0.520198899	4.735679392	1.557732569
N	1.546591363	1.709839967	0.849938490
AL	0.931024891	0.006147722	0.186517575
N	1.546584113	-1.709414028	0.824676694
C	0.665860971	-2.616298224	1.438898426
C	-0.096985770	-2.184768980	2.538109056
C	-1.030364936	-3.028195865	3.139980619
C	-1.218406674	-4.326456080	2.664373097
C	-0.469866485	-4.764288339	1.567190004
C	0.455510648	-3.922329914	0.955629759
C	2.806839173	2.114282909	0.433185697
C	3.519396062	1.186039460	-0.340494475
C	4.849677084	1.249112978	-0.708626598
C	5.526483617	2.428765108	-0.360639201
C	4.845421296	3.429656010	0.342092580
C	3.516197350	3.290584965	0.750864247
O	2.787680038	0.010132235	-0.615910056
C	3.525327766	-1.162156247	-0.344095052
C	4.857609325	-1.212768937	-0.708885214
C	5.444039558	0.020371375	-1.412905258
C	5.544260896	-2.385828281	-0.359214198
C	4.871091581	-3.392800325	0.341988017
C	3.538332787	-3.268169115	0.743470402
C	2.815940653	-2.101367093	0.419888787
C	6.972567305	0.025796668	-1.362833197
C	4.987476971	0.019146056	-2.894385713
AG	-1.068848174	0.042102157	-1.317942243
P	-3.408052509	-0.012428932	-0.737289333
C	-3.957965314	-1.844349781	-0.881423150
C	-3.499171600	-2.607927993	0.371203929
C	-3.848175476	0.651385276	1.012480387
C	-3.641613418	2.175032501	1.014265486
C	-4.337151396	1.048324073	-2.051002487
C	-4.313606343	0.324484089	-3.407162098
C	-5.270392971	0.321563242	1.488101932
C	-2.842651406	0.085895972	2.027520514
C	-5.791795242	1.390362701	-1.700202016
C	-3.533363718	2.347921576	-2.258079986
C	-5.467127053	-2.051327352	-1.067914995
C	-3.196538745	-2.474637789	-2.063526316
H	3.072558415	-4.050795462	1.339492864
H	6.594915326	-2.509855212	-0.614082954
H	6.575114504	2.562922108	-0.618676745
H	3.041916406	4.068499004	1.346938573
H	7.367024849	0.911197908	-1.877028457
H	7.373800299	-0.856119525	-1.877805558
H	7.343527191	0.026922633	-0.329821100
H	5.370072554	0.914030819	-3.403685613
H	3.893740427	0.015330164	-2.976748022
H	5.376612256	-0.872373143	-3.404627159
H	5.412470385	-4.300709959	0.611695096
H	5.378457556	4.343580163	0.608206811
H	-4.011068384	2.921304030	-3.067272307
H	-3.496059363	2.987015549	-1.372694850
H	-2.503980613	2.122426393	-2.570365464
H	-4.699471052	1.022409528	-4.165473774
H	-3.289152900	0.051142746	-3.693200522
H	-4.948436095	-0.567244825	-3.430247721
H	-6.232906477	1.938062833	-2.547263124
H	-6.403587453	0.497219743	-1.529447148
H	-5.870484085	2.038669718	-0.820905760
H	-3.470746813	-3.539309936	-2.118660776
H	-3.425378622	-2.014998731	-3.027593630
H	-2.109704756	-2.417274302	-1.906265499
H	-3.629394107	-3.683547367	0.179430917
H	-2.438197004	-2.438947527	0.593581403
H	-4.091688149	-2.360766890	1.258226360
H	-5.668919625	-3.133576775	-1.086592648
H	-6.052579766	-1.620037262	-0.248495050
H	-5.836501773	-1.635396717	-2.011389504
H	-3.721280445	2.527841273	2.052582678
H	-2.638689356	2.448973044	0.659163061
H	-4.393179762	2.710521464	0.425872223
H	-5.425885388	0.802665568	2.466285162
H	-6.048159275	0.689140289	0.811630770
H	-5.415914763	-0.755395243	1.631352688
H	-2.986394552	0.615906322	2.981053498
H	-2.966026238	-0.982711093	2.212964721
H	-1.812608892	0.257242330	1.692657669
H	0.068109757	-1.179564301	2.929971841
H	-1.606287426	-2.668909587	3.993886713
H	-1.941647843	-4.989669182	3.138861236
H	-0.618318348	-5.770976507	1.173507110
H	1.013264423	-4.264308846	0.083975927
H	0.868476273	4.180341962	0.000784920
H	-0.736866443	5.707585941	1.111952327
H	-1.870125413	5.020455433	3.225659591
H	-1.380211065	2.780272506	4.205972906
H	0.261630007	1.272444369	3.108668460
H	0.252434289	0.069934982	-2.245763532
H	-0.949409113	0.095789453	-3.055977970

96

INT_{Ag}

C	-2.748833537	-2.175471585	-0.336386405
C	-3.588378516	-1.245136479	0.286679487
C	-4.964723110	-1.292794141	0.390210347
C	-5.568083903	-2.468501737	-0.084116406
C	-4.772204744	-3.474945320	-0.642155170
C	-3.387364175	-3.346704754	-0.787948553
C	-5.677374536	-0.063112871	0.972116745
C	-4.943896274	1.173353294	0.431753667
C	-3.570186570	1.102642877	0.320131396
O	-2.902307490	-0.082819615	0.698200536
C	-5.523864504	2.383520476	0.016996183
C	-4.706762085	3.405345537	-0.479158503
C	-3.323380447	3.259398832	-0.628252581
C	-2.711802372	2.048041965	-0.252091845
N	-1.380671298	1.663585077	-0.390454515
AL	-0.963095052	-0.085530823	0.214333023
AG	1.240103475	0.122672712	1.630582246
P	3.383204907	0.068261086	0.492406403
C	3.484798946	0.551558579	-1.371156224
C	4.768999885	0.110438893	-2.090456990
C	-7.160542356	-0.043218755	0.599471349
C	-5.541301938	-0.089394360	2.516028990
N	-1.416547011	-1.794230408	-0.485265283
C	-0.541041061	-2.747444942	-1.073428957
C	-0.020544198	-2.530616613	-2.354625972
C	0.828448336	-3.470318960	-2.942470227
C	1.153359944	-4.645248883	-2.264268828
C	0.631714940	-4.870895918	-0.987397965
C	-0.203435001	-3.927562721	-0.392451290
C	-0.483490107	2.686703985	-0.817296448
C	-0.265949229	2.924899959	-2.179963540
C	0.545877919	3.983378369	-2.589012352
C	1.152932313	4.810367599	-1.641757510
C	0.958166901	4.562953054	-0.281421083
C	0.144395083	3.506562081	0.130070515
C	4.541600471	1.253769630	1.477971565
C	5.892443507	1.547693011	0.809433924
C	4.012030231	-1.736273130	0.692233487
C	5.534166620	-1.910834392	0.591717119
C	3.771211145	2.569380824	1.705481137
C	4.808078243	0.673833002	2.877574895
C	3.527447834	-2.267067569	2.055077566
C	3.338676187	-2.618402893	-0.368583855
C	2.274325706	-0.049774327	-2.098611061
C	3.333763761	2.073261182	-1.497579675
H	-2.809986692	-4.130025835	-1.276214349
H	-6.647759579	-2.594980486	-0.035201480

H	-6.600902985	2.528669884	0.072773189
H	-2.725468526	4.061998195	-1.057558407
H	-7.651067489	0.837202717	1.033034558
H	-7.667935936	-0.928581812	1.002623656
H	-7.302947613	-0.022799297	-0.488669360
H	-6.017296378	0.801197089	2.947910015
H	-4.488779447	-0.103733091	2.824775802
H	-6.033260626	-0.985015577	2.918732638
H	-5.251166176	-4.385793018	-1.003739806
H	-5.166578417	4.346622349	-0.783216704
H	4.388681979	3.223034433	2.340746403
H	3.547136427	3.111093496	0.783371291
H	2.829690766	2.373327000	2.237517442
H	5.323827128	1.448827424	3.465099791
H	3.866650074	0.430297552	3.386974980
H	5.457584866	-0.207606554	2.858611727
H	6.476608557	2.188203841	1.488252360
H	6.477599885	0.638751020	0.630114230
H	5.792376464	2.088042484	-0.137430604
H	3.853108844	-3.314878371	2.147640171
H	3.922521998	-1.709589188	2.907310430
H	2.430470233	-2.247946345	2.121670606
H	3.546679730	-3.669684750	-0.120815208
H	2.249350292	-2.493099396	-0.372463423
H	3.718451897	-2.441497908	-1.379699926
H	5.761884674	-2.985696105	0.666264423
H	5.937895893	-1.553207842	-0.361291330
H	6.069546496	-1.408719947	1.404324813
H	3.199673467	2.315359451	-2.561997108
H	2.444692095	2.433354293	-0.968702115
H	4.207782279	2.626908449	-1.142132353
H	4.739442502	0.505878039	-3.117848188
H	5.675511634	0.496543314	-1.613812427
H	4.854881502	-0.978901625	-2.166147015
H	2.274331900	0.317877822	-3.136228884
H	2.276189579	-1.140220159	-2.124554144
H	1.337167398	0.278372101	-1.626796222
H	-0.297659019	-1.620946728	-2.888437430
H	1.229227057	-3.284698412	-3.939812582
H	1.811704380	-5.381512638	-2.725899610
H	0.887123128	-5.782779338	-0.446466665
H	-0.606900424	-4.094162030	0.606879730
H	-0.027456176	3.314354554	1.189945087
H	1.439741381	5.195637532	0.464979385
H	1.784114613	5.639363014	-1.962681080
H	0.702796746	4.164056060	-3.653133359
H	-0.756770261	2.282086174	-2.911743164
H	-0.433958074	-0.082246835	1.881343498
H	1.681773785	0.390732854	3.288052885

96

TS$^{II}_{Ag}$

C	-0.553498112	3.246596946	0.231782929
C	0.239733595	2.483261172	1.097641571
C	0.069682833	2.614988663	2.481880745
C	-0.861539709	3.518934425	2.992800469
C	-1.636737795	4.293147996	2.126599910
C	-1.489040327	4.145869606	0.746645089
N	1.257148620	1.632376228	0.568168400
AL	1.083336720	-0.044911221	-0.277488774
N	1.671641249	-1.776605980	0.204135807
C	0.892864057	-2.910420431	0.571737064
C	0.358168582	-3.016342017	1.860248627
C	-0.390619760	-4.137208023	2.224275074
C	-0.596804483	-5.170951337	1.310526432
C	-0.057679726	-5.074109148	0.025027635
C	0.678754294	-3.950841754	-0.344763334
C	2.543274175	2.171178122	0.536672364
C	3.514288694	1.415189152	-0.128482948
C	4.874416212	1.643561641	-0.177024234
C	5.307208839	2.838149360	0.420928654
C	4.369843746	3.688241846	1.018686237
C	3.008505463	3.377002732	1.094453376
O	2.991935843	0.227331934	-0.693770062
C	3.791005026	-0.902689566	-0.403680963
C	5.165832425	-0.790184854	-0.460792017
C	5.750416549	0.575912134	-0.850602363
C	5.881222212	-1.949768607	-0.121950488
C	5.185523702	-3.100282072	0.267567869
C	3.792135749	-3.137540125	0.378422114
C	3.042887511	-1.988639458	0.062264554
C	7.214992900	0.701026658	-0.428604050
C	5.647872760	0.743437111	-2.388220298
AG	-1.411973272	0.527189510	-1.605291060
P	-3.431853897	-0.155484005	-0.405660118
C	-3.789230600	-1.947922624	-1.011638348
C	-2.904570370	-2.926525338	-0.227042901
C	-3.458911117	-0.143714443	1.520338678
C	-3.572347872	1.303374800	2.018719842
C	-4.844342392	0.997712101	-1.032771563
C	-5.071920527	0.735735507	-2.531636612
C	-4.570048789	-0.981070044	2.170613769
C	-2.091104661	-0.647923300	2.004700157
C	-6.182657990	0.863067797	-0.294155391
C	-4.340866410	2.450986682	-0.936052837
C	-5.250517441	-2.405341920	-0.905639715
C	-3.326716781	-2.053779008	-2.478685599
H	3.289991575	-4.035575693	0.734300942
H	6.969093738	-1.956026461	-0.147389105
H	6.362617567	3.103121060	0.427369104
H	2.316495166	4.045503772	1.603819951
H	7.615007759	1.678844824	-0.724573610
H	7.822585466	-0.063294098	-0.929126559
H	7.334020351	0.587678134	0.656620703
H	6.032785216	1.729087163	-2.682527420
H	4.609818821	0.660816614	-2.732706013
H	6.241894126	-0.033267768	-2.888445718
H	5.751274682	-3.997169763	0.522994510
H	4.714225238	4.622249614	1.464335710
H	-5.091504005	3.105401199	-1.405801788
H	-4.193755411	2.793569433	0.090826015
H	-3.395844766	2.567491869	-1.484608247
H	-5.742355788	1.521430121	-2.912288001
H	-4.128620880	0.798653792	-3.090893737
H	-5.552310166	-0.228148203	-2.729686118
H	-6.913065853	1.532003012	-0.775917923
H	-6.588037282	-0.153711570	-0.337814192
H	-6.115878031	1.162539990	0.757362732
H	-3.457916910	-3.097267525	-2.805485910
H	-3.887079080	-1.412271590	-3.162577830
H	-2.260489159	-1.802473059	-2.576463268
H	-2.979393614	-3.914538380	-0.704964673
H	-1.848332287	-2.632243562	-0.254258267
H	-3.209920671	-3.047664462	0.816639382
H	-5.308963127	-3.457761203	-1.225656559
H	-5.635791537	-2.349698706	0.118254642
H	-5.917502611	-1.831239289	-1.557670895
H	-3.380861833	1.306477769	3.102456111
H	-2.821819783	1.950807723	1.553160629
H	-4.564718526	1.736614459	1.859086411
H	-4.512318656	-0.846259100	3.262248932
H	-5.570101943	-0.668173594	1.851572282
H	-4.464489521	-2.052592888	1.970378857
H	-2.062025897	-0.590504723	3.103947629
H	-1.878765506	-1.677879645	1.715010278
H	-1.286718716	-0.004937356	1.618673877
H	0.542962942	-2.214366754	2.575557442
H	-0.805882109	-4.204460481	3.230465540
H	-1.176622524	-6.048552398	1.597286466
H	-0.219317929	-5.874895328	-0.697441096
H	1.098222524	-3.865629743	-1.347739197
H	-0.416866030	3.141304047	-0.846414041
H	-2.100696600	4.735444352	0.063408180
H	-2.362402954	5.001582973	2.526848078
H	-0.982010609	3.619012481	4.072127696
H	0.689461754	2.014700825	3.149004645
H	0.296265269	0.004949517	-1.732790170
H	-1.592264272	1.431128718	-3.065573132

96

PC'$_{Ag}$

C	2.956125853	2.153546552	-0.312576139
C	3.662474157	1.190456451	0.426507764
C	4.974860210	1.260127616	0.853579663

C	5.643945745	2.464575041	0.580930208
C	4.973392899	3.486522051	-0.100048649
C	3.657158144	3.351912410	-0.551730075
C	5.553889091	0.038501602	1.581776311
C	4.980722966	-1.216455756	0.908338258
C	3.669661598	-1.169638969	0.475478966
O	2.928133604	0.012038485	0.622352045
C	5.654705294	-2.429111846	0.688126281
C	4.990073863	-3.480653686	0.047497254
C	3.676505887	-3.368033635	-0.418108351
C	2.972537237	-2.162005324	-0.233183781
N	1.735552856	-1.782674898	-0.731863117
AL	1.119991733	-0.013657115	-0.343894967
AG	-1.308686257	-0.035020437	0.522958597
P	-3.676664124	-0.001608145	0.599565263
C	-4.342634115	-1.658008814	-0.102969424
C	-5.797799912	-1.606515898	-0.589105852
C	7.083604774	0.041936552	1.558201701
C	5.069529575	0.070408294	3.054553330
N	1.713015873	1.754001805	-0.783945020
C	0.838752362	2.699051236	-1.363420932
C	0.191494185	2.401703869	-2.575264296
C	-0.720513472	3.294807115	-3.135459290
C	-0.997855947	4.510903409	-2.507982393
C	-0.358952676	4.817080373	-1.303664257
C	0.543190255	3.921278401	-0.732605852
C	0.877613906	-2.746207793	-1.306457270
C	0.296960574	-2.502637418	-2.562439265
C	-0.598544508	-3.412207718	-3.122509018
C	-0.927465400	-4.588937617	-2.446614372
C	-0.354713317	-4.841115614	-1.197250467
C	0.533392794	-3.929324403	-0.628650566
C	-4.372832110	0.274500626	2.366954791
C	-5.872529297	-0.011074535	2.525158415
C	-4.207840739	1.448801507	-0.540783206
C	-5.673058727	1.881139899	-0.399997628
C	-3.568524496	-0.609530468	3.339748627
C	-4.088573149	1.725642749	2.787177069
C	-3.278458294	2.643590147	-0.248774855
C	-3.931866577	1.062098086	-2.003839635
C	-3.419094870	-2.091223018	-1.257056921
C	-4.210643216	-2.752538878	0.968700391
H	3.187426877	4.154238880	-1.118285801
H	6.678222063	2.605870071	0.888525151
H	6.688327076	-2.554072010	1.004882637
H	3.211168042	-4.193213815	-0.954960322
H	7.476321102	-0.828476455	2.098514078
H	7.471625758	0.936635931	2.060890482
H	7.470820061	0.020833592	0.531372060
H	5.446245108	-0.810614683	3.592032801
H	3.973775590	0.068895385	3.111564671
H	5.441707782	0.976464511	3.552067608
H	5.501302226	4.418838605	-0.305907059
H	5.521535917	-4.419342842	-0.116090816
H	-3.865872937	-0.346782504	4.366364956
H	-3.747060870	-1.678979685	3.204997010
H	-2.490382233	-0.424437249	3.236687890
H	-4.326767788	1.821214828	3.857135902
H	-3.028163682	1.981993807	2.658504750
H	-4.700312043	2.455684315	2.247549097
H	-6.168192229	0.230806741	3.557712377
H	-6.488641796	0.596530994	1.853124426
H	-6.116268755	-1.065966342	2.356830114
H	-3.514550850	3.445709079	-0.963557759
H	-3.388707623	3.050245251	0.758967816
H	-2.223294103	2.376909869	-0.403828059
H	-4.072970595	1.962773798	-2.619908456
H	-2.894747154	0.728358513	-2.141788849
H	-4.613290399	0.293118884	-2.381932144
H	-5.875353199	2.673724493	-1.136864974
H	-6.373577054	1.061253895	-0.594054094
H	-5.892774949	2.295878600	0.590114847
H	-4.426676535	-3.719339272	0.489909229
H	-3.190599658	-2.803978064	1.372157318
H	-4.916984260	-2.631179442	1.796197713
H	-6.091244319	-2.615123914	-0.919244399
H	-6.493661859	-1.300235758	0.199955158

H	-5.928193803	-0.934469712	-1.444217750
H	-3.753215458	-3.077520121	-1.612925683
H	-3.429429269	-1.407280971	-2.108457800
H	-2.378293762	-2.198184077	-0.923650375
H	0.414453670	1.457165306	-3.070921091
H	-1.210760702	3.040371470	-4.076305893
H	-1.706082013	5.211838127	-2.950728690
H	-0.573356386	5.758543807	-0.795498807
H	1.022429732	4.157555085	0.217688359
H	0.964081060	-4.121654193	0.354455492
H	-0.609363464	-5.752338297	-0.653812454
H	-1.624703484	-5.301780055	-2.887657805
H	-1.038393142	-3.201354502	-4.098311959
H	0.559194368	-1.586511538	-3.091654918
H	-0.341152930	-0.038445767	-1.204927925
H	0.350649741	-0.008264412	1.267081099

96

TS^{IV}_{Ag}

C	-0.049930536	2.374686086	1.812784952
C	0.754709284	2.695500588	0.705872718
C	0.528657587	3.912646383	0.043910568
C	-0.476426943	4.778295699	0.475528201
C	-1.273247097	4.450146048	1.574790953
C	-1.051205510	3.243975426	2.242953095
N	1.729325729	1.772090167	0.264953355
AL	1.262164510	-0.004179418	-0.255276118
AG	-1.349757142	-0.009775007	-0.217022182
P	-3.830447102	-0.001439192	-0.287909666
C	-4.345853547	1.418762933	-1.471331999
C	-3.486124328	2.650962956	-1.122631210
C	3.053881364	2.171035648	0.132598876
C	3.930779747	1.186088372	-0.350325921
C	5.308843441	1.250590564	-0.415454159
C	5.881580133	2.472986155	-0.026120202
C	5.054798087	3.514406895	0.407969068
C	3.664686154	3.385082012	0.500400602
O	3.270794198	-0.001301545	-0.679533096
C	3.939814525	-1.175290621	-0.320970761
C	5.318489531	-1.230280060	-0.384499818
C	6.059878153	0.006721600	-0.912477758
C	5.900920341	-2.438258915	0.034094673
C	5.082465829	-3.475392187	0.493373627
C	3.691419609	-3.355101924	0.582193967
C	3.070352608	-2.155587948	0.184551475
N	1.741934895	-1.765158999	0.305701145
C	0.770672837	-2.689512677	0.751178515
C	-0.066026510	-2.348850585	1.828487779
C	-1.067395321	-3.218588244	2.257766414
C	-1.253022436	-4.448485342	1.622642144
C	-0.425061636	-4.796344236	0.552730341
C	0.576736498	-3.928203994	0.119020269
C	7.524256666	0.018198094	-0.467763943
C	6.000216694	-0.013282581	-2.461576105
C	-4.416033928	-1.682550073	-1.009544538
C	-3.411995638	-2.099500463	-2.104173905
C	-4.647590418	0.272860960	1.426274677
C	-3.829652295	-0.538425475	2.450244466
C	-4.299703914	-2.757748620	0.083159747
C	-5.840253257	-1.693769954	-1.580541298
C	-4.482542149	1.746061429	1.831270143
C	-6.132126973	-0.101874894	1.521048341
C	-3.954103251	1.026444284	-2.905878617
C	-5.832888161	1.794647314	-1.445376451
H	3.095845058	-4.170923548	0.988327511
H	6.980757087	-2.571032075	0.008394769
H	6.960366462	2.613484453	-0.054802807
H	3.062091757	4.205317532	0.886934099
H	8.040113877	0.897918842	-0.872452791
H	8.047136168	-0.867360960	-0.850070673
H	7.613325588	0.032493537	0.626225625
H	6.498672284	0.877619500	-2.867564600
H	4.962187410	-0.022404017	-2.817385479
H	6.506634800	-0.909893848	-2.844464037
H	5.545244519	-4.410212243	0.812869348
H	5.510169706	4.460294543	0.704727978
H	-3.646465176	3.414741759	-1.899100817

H	-3.734953690	3.100307987	-0.158778069
H	-2.415068471	2.401291198	-1.109638589
H	-4.084643511	1.910809439	-3.547625494
H	-2.900033834	0.722835416	-2.968544110
H	-4.579514871	0.227375418	-3.316726538
H	-6.008815758	2.587441380	-2.189499984
H	-6.482191187	0.949999489	-1.701155048
H	-6.147812309	2.188116594	-0.472540109
H	-4.771110931	1.840854685	2.889392454
H	-3.441575608	2.079829048	1.738130793
H	-5.124003136	2.421234761	1.255671333
H	-4.206402731	-0.308120786	3.458723502
H	-3.899163208	-1.619027503	2.303938875
H	-2.767261598	-0.259012619	2.410798731
H	-6.492383551	0.139677296	2.533587847
H	-6.747397807	0.459059483	0.808128751
H	-6.308129606	-1.171007392	1.360456426
H	-6.077235477	-2.716457752	-1.913935185
H	-6.591401986	-1.404608447	-0.836854355
H	-5.946373090	-1.036352682	-2.450728045
H	-3.673088286	-3.114732182	-2.440688976
H	-3.421199416	-1.445189699	-2.979132016
H	-2.387181597	-2.128761407	-1.706366549
H	-4.472814764	-3.737521364	-0.387555926
H	-3.295150098	-2.780960976	0.524996813
H	-5.040789538	-2.643258397	0.880820169
H	0.097013734	-1.397878192	2.337355167
H	-1.700408554	-2.935148892	3.099556152
H	-2.035748387	-5.129274773	1.958087600
H	-0.564567233	-5.750855423	0.043216814
H	1.207369909	-4.196469139	-0.728860946
H	1.136345224	4.165282460	-0.825335316
H	-0.642550286	5.715379061	-0.057847692
H	-2.056831423	5.130016607	1.910302445
H	-1.651923705	2.981729558	3.114826728
H	0.139609906	1.441035366	2.344174914
H	0.134110930	-0.022934451	-1.510505526
H	1.102499554	-0.028903768	-2.106263348

96

PC_{Ag}

C	0.408065765	-2.623233744	-3.330091452
C	-0.007223470	-2.746392612	-1.988219660
C	0.656473831	-3.694513776	-1.183923376
C	1.677124946	-4.487406631	-1.704033591
C	2.076237566	-4.356112926	-3.035858622
C	1.436134413	-3.411593562	-3.841560422
N	-0.992182653	-1.865271756	-1.510702441
AL	-1.000131551	0.025425886	-2.105884998
N	-1.093997161	1.897665968	-1.463776971
C	-0.138342815	2.830922746	-1.897905045
C	0.487186790	3.766910523	-1.049740982
C	1.492759176	4.606140932	-1.525286562
C	1.911975564	4.536552858	-2.855655848
C	1.306403308	3.608189821	-3.705695242
C	0.294456734	2.772955739	-3.238494672
C	-1.944886802	-2.261464829	-0.590544068
C	-2.822623451	-1.241975908	-0.171281468
C	-3.823397930	-1.336816620	0.774918278
C	-4.006255226	-2.608278944	1.346683275
C	-3.219164442	-3.678804180	0.917559983
C	-2.206962358	-3.530927748	-0.034157283
O	-2.571793211	-0.032627097	-0.828693759
C	-2.888292751	1.145081161	-0.142656476
C	-3.893062959	1.163058499	0.803777370
C	-4.698855928	-0.113463438	1.065586730
C	-4.142486727	2.408387585	1.407014208
C	-3.407680713	3.528170689	1.011630005
C	-2.385286786	3.456929253	0.061905809
C	-2.062603793	2.218701182	-0.531431600
C	-5.230240358	-0.144484341	2.504167239
C	-5.894292080	-0.136615076	0.078751836
AG	1.388843422	0.089222987	-0.629343089
P	2.180322352	0.022069167	1.614908802
C	0.723258127	-0.550667979	2.724807227
C	0.457261223	-2.038347138	2.445972013
C	3.631231392	-1.226419983	1.723590006
C	4.880362460	-0.593282648	1.088783826
C	2.776870537	1.755668928	2.170281256
C	1.550306967	2.645289679	2.426429816
C	3.962411607	-1.697058450	3.145971351
C	3.284860912	-2.441310327	0.844999048
C	3.657689777	1.753500593	3.426845247
C	3.534544120	2.399278463	0.992241369
C	0.938264350	-0.344729926	4.229821322
C	-0.547897201	0.190724292	2.269491536
H	-1.849623182	4.359052903	-0.222608230
H	-4.919020297	2.510564102	2.162245284
H	-4.774076151	-2.769089261	2.100569029
H	-1.629486655	-4.397428630	-0.345808716
H	-5.831745199	-1.045278094	2.674719591
H	-5.882518767	0.716349302	2.693374754
H	-4.409011590	-0.128050807	3.232482210
H	-6.487071996	-1.049864425	0.225581329
H	-5.542914283	-0.115238615	-0.960847046
H	-6.538157301	0.737888013	0.245033639
H	-3.636615056	4.496610647	1.458758247
H	-3.397727424	-4.669529904	1.338136459
H	3.806027446	3.426211129	1.280550410
H	4.455027914	1.874607080	0.725540740
H	2.898328913	2.463216016	0.098912674
H	1.901173210	3.681936002	2.538545476
H	0.849848024	2.621313303	1.583628982
H	1.013246540	2.378410325	3.341929397
H	3.882927352	2.797131870	3.694448323
H	3.157714309	1.292218008	4.286089693
H	4.615085826	1.245439376	3.269794966
H	-1.413442951	-0.265771238	2.771393685
H	-0.550773663	1.257292650	2.501822779
H	-0.700097934	0.076806309	1.188134195
H	-0.489030918	-2.313288795	2.934258897
H	0.332468928	-2.226343890	1.372171856
H	1.238617984	-2.695602932	2.840843226
H	0.065411456	-0.751446252	4.763058416
H	1.826444797	-0.865923386	4.603274507
H	1.018982281	0.713590468	4.500783278
H	5.649636914	-1.375706485	1.008662424
H	4.676171537	-0.225790839	0.073964521
H	5.302307984	0.222092060	1.684267104
H	4.839717997	-2.360102684	3.098306093
H	4.208526754	-0.868740883	3.819041135
H	3.144136504	-2.274154934	3.590914968
H	4.163678313	-3.102991309	0.811440560
H	2.443482514	-3.025986863	1.221817505
H	3.050513272	-2.142543055	-0.185610305
H	0.199504682	3.821041669	-0.002766633
H	1.961913624	5.315544407	-0.841400453
H	2.699002755	5.194800907	-3.223861297
H	1.618936444	3.536588378	-4.748579278
H	-0.184491383	2.062324528	-3.913037946
H	-0.099488593	-1.901810004	-3.971463369
H	1.733095657	-3.291468071	-4.884518348
H	2.874957571	-4.979036152	-3.438680186
H	2.175646035	-5.207607696	-1.053058488
H	0.390063710	-3.792613878	-0.134049012
H	-1.499986784	0.032019254	-3.631675103
H	0.790634335	0.081576668	-2.254866382

96

PC_{Ag}-HH

C 2.035475872 2.041348815 1.063815042
C 3.089459021 1.471278549 0.333505495
C 3.731911740 2.013100395 -0.763156141
C 3.363273934 3.323114617 -1.103221616
C 2.375272039 3.975924790 -0.359615992
C 1.704177823 3.360870685 0.699012441
C 4.729478665 1.124641168 -1.516997139
C 4.109042970 -0.277535775 -1.566111851
C 3.461218502 -0.732375165 -0.433631061
O 3.362134332 0.124909744 0.695496505
C 4.091655633 -1.144576404 -2.669178374
C 3.421766662 -2.369707707 -2.574734424
C 2.732631647 -2.761063249 -1.425070923
C 2.716043312 -1.912964780 -0.301318984

N 1.987257129 -2.032481834 0.874120080
AL 2.093236663 -0.565975985 2.050094881
AG -2.825365285 -0.896981696 0.632903666
P -2.688975999 0.404112039 -1.390595566
C -1.017122208 0.149903226 -2.292891214
C -0.691258027 1.185972692 -3.377066596
C 5.014613038 1.663040843 -2.919312017
C 6.055085518 1.062970269 -0.717194401
N 1.405564525 1.189574213 1.962932452
C 0.378064668 1.632766496 2.820402267
C -0.736757680 0.801129233 3.009920608
C -1.767931025 1.163883311 3.873018636
C -1.710112814 2.375870422 4.561831465
C -0.599860611 3.207653604 4.387910342
C 0.439648067 2.842856018 3.534319626
C 1.335484515 -3.239270516 1.213177159
C 0.025109718 -3.188541702 1.708073043
C -0.630808519 -4.348722713 2.116011757
C 0.008626364 -5.585158622 2.025479411
C 1.314946683 -5.645559478 1.528877988
C 1.977748182 -4.487071648 1.129826897
C -4.121688814 -0.146973462 -2.542765493
C -4.003067563 0.314818711 -4.000981737
C -2.901682759 2.262867619 -0.954032524
C -3.289663631 3.169234343 -2.129042252
C -4.202272767 -1.686046619 -2.493019075
C -5.450334189 0.359566539 -1.957321361
C -3.962058969 2.367077011 0.161192384
C -1.597421103 2.783241143 -0.330838860
C 0.092958898 0.157505435 -1.228679441
C -0.988202199 -1.257898628 -2.911654800
H 0.895665775 3.880895919 1.207343596
H 3.828803927 3.825704220 -1.948561256
H 4.582703698 -0.864143221 -3.598707541
H 2.175138224 -3.695547405 -1.408683633
H 5.732047287 1.014549057 -3.437182596
H 5.462181779 2.662820573 -2.860046327
H 4.099650321 1.724596425 -3.522709059
H 6.767678634 0.399949997 -1.225863285
H 5.893300174 0.682840254 0.298855344
H 6.494117091 2.067182718 -0.645725938
H 2.094679602 4.994249996 -0.631485491
H 3.413498839 -3.035300517 -3.438732232
H -5.099585701 -2.001351229 -3.047399186
H -3.340033831 -2.182020257 -2.945081683
H -4.300147385 -2.043581700 -1.457927669
H -6.270725193 -0.109151360 -2.521562233
H -5.563565965 0.069840262 -0.903788153
H -5.570937415 1.444226149 -2.042764448
H -4.902275655 -0.011151499 -4.546870474
H -3.936480525 1.404631396 -4.092497371
H -3.137932644 -0.127591939 -4.507367929
H -4.012382325 3.417220909 0.487963374
H -4.963797557 2.067585520 -0.156012108
H -3.678401813 1.757258924 1.030720447
H -1.794300607 3.788259647 0.072634401
H -1.266850560 2.154831980 0.505979081
H -0.778241516 2.875959689 -1.050342735
H -3.330414242 4.209242870 -1.769208697
H -2.560691980 3.129783040 -2.946248498
H -4.277719057 2.928778490 -2.536815047
H 0.042208383 -1.459810611 -3.241306706
H -1.253626754 -2.027547440 -2.174178810
H -1.642285362 -1.357554617 -3.784014490
H 0.264264889 0.907260218 -3.848381416
H -1.451786481 1.227086455 -4.164475520
H -0.565022580 2.193048390 -2.964269618
H 1.043975733 -0.084309566 -1.725408509
H 0.215640433 1.119431350 -0.729090661
H -0.088934908 -0.609574071 -0.463809485
H -0.825100642 -0.128087001 2.440220808
H -2.624364985 0.497267828 3.980901189
H -2.519333137 2.670507306 5.230029091
H -0.534624486 4.149815588 4.934196701
H 1.313957165 3.485066165 3.434597237
H 3.005015540 -4.539207249 0.768730314
H 1.829229252 -6.605369973 1.462885001
H -0.503900513 -6.495825259 2.336102745
H -1.654632346 -4.272236515 2.484508232
H -0.512908302 -2.238073329 1.741415722
H -3.004457086 -1.778090155 2.034832749
H 2.374309424 -1.016260434 3.541742224

102

RC^{cat}_{Ag}

C	1.983514463	-2.282410898	-0.471060297
C	2.884034872	-1.206732545	-0.331369471
C	4.020867854	-1.165090745	0.450549109
C	4.320965953	-2.342714662	1.158237932
C	3.501106207	-3.463750024	1.014777464
C	2.354273635	-3.457076917	0.216430503
C	4.901783761	0.088396218	0.424931862
C	4.032646942	1.314373015	0.125966054
C	2.889354069	1.163611019	-0.633474830
O	2.498442624	-0.098310140	-1.095533792
C	4.342925955	2.630733390	0.509931843
C	3.524968116	3.683762710	0.094527771
C	2.364207159	3.479874883	-0.655557181
C	1.978793656	2.170868572	-1.013160993
N	0.872476202	1.721552876	-1.709339262
AL	0.739963850	-0.221641362	-2.094982971
AG	-1.307978326	-0.059355268	-0.197997772
P	-1.705001529	0.229819678	2.129852144
C	-2.061523015	-1.463677889	2.949481232
C	-2.978096911	-2.267808584	2.006918440
C	5.659246575	0.257741561	1.747320680
C	5.923207101	-0.068642243	-0.730825613
N	0.905367866	-2.029439150	-1.295604936
C	-0.114761887	-2.970984848	-1.509072266
C	-0.650597960	-3.796060677	-0.499525590
C	-1.716446525	-4.653615501	-0.765089122
C	-2.289126523	-4.709938375	-2.037466439
C	-1.776616209	-3.887781811	-3.043564351
C	-0.706830366	-3.034535582	-2.786690884
C	-0.188079230	2.568881724	-2.068349244
C	-0.884375515	2.293246476	-3.263553564
C	-2.005774447	3.031346953	-3.632190807
C	-2.466752860	4.076020007	-2.828212467
C	-1.787837344	4.361288583	-1.641425047
C	-0.669767612	3.621250686	-1.262373511
C	-0.130253349	1.006437484	2.901861653
C	1.096545620	0.298857015	2.296411328
C	-3.203268765	1.401623394	2.357653602
C	-3.101306297	2.522859514	1.305405795
C	-0.035638180	2.469969845	2.441968480
C	-0.063285623	0.948869803	4.433053307
C	-4.491835620	0.630483308	2.026983014
C	-3.326114803	2.014035828	3.758988857
C	-0.743066948	-2.249653366	3.045593127
C	-2.694511458	-1.378957875	4.344677523
H	1.757965688	-4.361088100	0.121136263
H	5.199225443	-2.393661309	1.798418921
H	5.228506314	2.837129753	1.107407010
H	1.765742353	4.332756031	-0.965427589
H	6.310091284	1.139382317	1.710038557
H	6.304907290	-0.608403571	1.935586453
H	4.966909555	0.368450029	2.592025975
H	6.563852709	0.821779240	-0.791284401
H	5.409232562	-0.191973956	-1.692636339
H	6.554126192	-0.951583115	-0.559309300
H	3.764383246	-4.382880744	1.540322532
H	3.798574635	4.705085904	0.363412969
H	-3.084225860	-3.283455633	2.417767686
H	-3.980080763	-1.843645389	1.908783772
H	-2.539430467	-2.361286891	1.004339099
H	-0.986489376	-3.288133251	3.314969767
H	-0.207177455	-2.270574257	2.088466306
H	-0.071161040	-1.859345195	3.816434198
H	-2.794717544	-2.400338965	4.742750956
H	-2.080373711	-0.807274121	5.049386713
H	-3.697611574	-0.939132087	4.323493398
H	1.998362719	0.860050089	2.581857448
H	1.225643854	-0.733620740	2.626773881
H	1.045277067	0.302849795	1.199831269
H	0.956389038	2.852396732	2.723434643

H	-0.116445899	2.549294988	1.350822271
H	-0.786503575	3.116619522	2.907172531
H	0.852299218	1.466191199	4.758177021
H	-0.913248456	1.448332831	4.911073721
H	-0.007162611	-0.077796164	4.811112249
H	-5.319121521	1.354800068	1.997249920
H	-4.436183583	0.150617283	1.040392203
H	-4.744278169	-0.126204100	2.776400198
H	-4.243154967	2.621764702	3.794652876
H	-3.400433779	1.253637853	4.544411290
H	-2.487491331	2.677316514	3.997863810
H	-4.022352019	3.123153430	1.354429257
H	-2.256151959	3.195887303	1.463388198
H	-3.019046451	2.116857626	0.287536032
H	-0.246069311	-3.746675936	0.508510593
H	-2.112185288	-5.276169360	0.039182712
H	-3.122602476	-5.382599782	-2.240475894
H	-2.210641491	-3.913520495	-4.044412934
H	-0.309553074	-2.400536034	-3.579835074
H	-0.530197457	1.485003831	-3.904016036
H	-2.521102929	2.788827880	-4.562982361
H	-3.341374844	4.657608265	-3.120106525
H	-2.139730717	5.164235569	-0.991469214
H	-0.182067089	3.845076209	-0.316521805
H	0.978806687	-0.389179464	-3.673361638
H	-1.035794037	-0.227135549	-1.899679791
C	-3.392075978	-0.503520274	-3.571414137
C	-3.153147363	-0.578613807	-4.879191329
H	-3.577127409	0.452395476	-3.079302651
H	-3.382341168	-1.391341326	-2.937928838
H	-2.945594006	-1.531088300	-5.370185650
H	-3.142454702	0.310016835	-5.513021063

102

TS^{cat}_{Ag}

C	-2.081325318	-2.308903186	0.291743474
C	-2.861818174	-1.182396466	-0.036599919
C	-3.829892817	-1.103236199	-1.017830596
C	-4.067940736	-2.291693556	-1.733166939
C	-3.349333584	-3.450708161	-1.427796156
C	-2.366831831	-3.480565904	-0.434380697
C	-4.608261272	0.200467710	-1.225033828
C	-3.790237165	1.385181860	-0.698690746
C	-2.824379914	1.185333945	0.268172826
O	-2.500903430	-0.084124559	0.740393194
C	-3.997657561	2.723310325	-1.082990411
C	-3.260809800	3.747363194	-0.482871149
C	-2.287396934	3.497375863	0.488295564
C	-2.024188470	2.171128561	0.880960662
N	-1.113055744	1.682728541	1.794365074
AL	-0.746313999	-0.254665290	1.873816137
AG	1.417083676	-0.115950847	0.386124017
P	1.990522010	0.271201600	-1.966443447
C	2.596021407	-1.347958491	-2.798308737
C	3.463974096	-2.104067403	-1.772683054
C	-4.958150107	0.394658762	-2.708012787
C	-5.918967857	0.117336188	-0.402201533
N	-1.158747163	-2.095209682	1.293521368
C	-0.220157521	-3.102767256	1.613347369
C	0.658930309	-3.628719452	0.650535136
C	1.644987141	-4.545605705	1.006870361
C	1.777657789	-4.955333535	2.336592613
C	0.906005457	-4.443669357	3.299806564
C	-0.086821293	-3.530906656	2.942309945
C	-0.172872275	2.549635520	2.395243107
C	-0.006509917	2.525139713	3.788934919
C	0.981863547	3.293791815	4.403374043
C	1.817383630	4.113147246	3.641874406
C	1.652640035	4.154084861	2.254738625
C	0.672338467	3.380066039	1.638784804
C	0.433378780	0.915446072	-2.879734847
C	-0.759727785	0.051201839	-2.432444924
C	3.387481210	1.585683457	-2.039163887
C	3.078059115	2.668467547	-0.987995996
C	0.132010444	2.336655359	-2.376113853
C	0.522345098	0.928235740	-4.410688779
C	4.702863788	0.930040207	-1.586322171
C	3.589346117	2.246423808	-3.408584950
C	1.384088152	-2.250904497	-3.080330822
C	3.375904103	-1.155861844	-4.106128898
H	-1.818990419	-4.398104950	-0.225218571
H	-4.818975080	-2.317156448	-2.520431574
H	-4.743041318	2.968060385	-1.837413421
H	-1.729290867	4.319164097	0.933582064
H	-5.535406274	1.315452414	-2.853334037
H	-5.576668194	-0.434539679	-3.071445326
H	-4.052463804	0.448907921	-3.326022539
H	-6.498840423	1.043043123	-0.520228785
H	-5.700139381	-0.021055337	0.664528592
H	-6.527131043	-0.731135164	-0.744706832
H	-3.561059810	-4.365682725	-1.983166258
H	-3.451134685	4.779719239	-0.780283957
H	3.726593373	-3.084560151	-2.198758145
H	4.395856154	-1.588586242	-1.527170305
H	2.912327358	-2.279638141	-0.838385039
H	1.759664814	-3.249110400	-3.350614687
H	0.740991023	-2.363729554	-2.199200805
H	0.772414175	-1.892513827	-3.914229843
H	3.615437354	-2.148127384	-4.519173049
H	2.793862495	-0.614661477	-4.860792869
H	4.324314465	-0.628469294	-3.958058646
H	-1.688131709	0.557220788	-2.732359903
H	-0.770326122	-0.950792405	-2.865981373
H	-0.779688614	-0.051430486	-1.339254288
H	-0.860422025	2.629491096	-2.748981211
H	0.084037177	2.372045146	-1.280043080
H	0.853945071	3.078691231	-2.732442177
H	-0.413477735	1.348884674	-4.810124034
H	1.347502336	1.547271827	-4.779416833
H	0.630931997	-0.077759642	-4.830875167
H	5.453579472	1.725225315	-1.464441384
H	4.590776481	0.429379727	-0.614796568
H	5.099758696	0.212461410	-2.310987122
H	4.445592361	2.935783549	-3.343963804
H	3.805983224	1.520152340	-4.199521305
H	2.718644978	2.838726977	-3.712235932
H	3.944739428	3.344033368	-0.924423805
H	2.203500961	3.274287378	-1.236192123
H	2.917110717	2.230693231	0.007098896
H	0.560857892	-3.300909935	-0.383235098
H	2.322566375	-4.933605596	0.244427864
H	2.553506064	-5.667785664	2.618270839
H	0.996637675	-4.760074831	4.339957790
H	-0.768474666	-3.126303842	3.690070874
H	-0.661635547	1.885725526	4.379758383
H	1.095614053	3.255446859	5.487764529
H	2.588901744	4.714817875	4.123178090
H	2.302843349	4.783752880	1.644923663
H	0.560408487	3.402083194	0.556135970
H	-0.205036275	-0.481540297	3.438205883
H	1.635164217	-0.391069292	2.197894472
C	2.336630411	-0.606936827	3.397547240
C	1.358694748	-0.696368258	4.393669358
H	2.957998889	0.291406590	3.366380014
H	2.854384495	-1.523028935	3.102728894
H	0.990547787	-1.661604807	4.732825971
H	1.087096085	0.171080528	4.991000533

102

PC^{cat}_{Ag}

C	0.755079616	3.643976609	-1.575002826
C	0.890600334	2.288538298	-1.935226493
C	0.016863836	1.773349962	-2.912738672
C	-0.950380506	2.580345282	-3.509482670
C	-1.067590045	3.924449582	-3.153175687
C	-0.208612048	4.446211219	-2.180708531
N	1.768377399	1.406849382	-1.289498371
AL	1.025017649	-0.194902028	-0.480211693
AG	-1.309222637	-0.013561552	0.272657057
P	-3.700094958	0.092152034	0.821076800
C	-4.539214724	-1.453889329	0.054092510
C	-4.571415064	-1.280565415	-1.473978436
O	2.812311929	-0.137387117	0.473781326
C	3.616937650	0.964576251	0.106866597

C	4.908807974	1.038450157	0.592234171
C	5.378018380	-0.095718884	1.513663993
C	4.809961911	-1.396873196	0.927627301
C	3.520698501	-1.358770590	0.429417706
C	5.663005199	2.141422690	0.164783720
C	5.093158496	3.060962486	-0.724076535
C	3.801805740	2.910429905	-1.234436186
C	3.013009885	1.812443914	-0.833194297
C	2.844658260	-2.376056055	-0.263454480
C	3.546560430	-3.596464791	-0.352419478
C	4.830456054	-3.701003432	0.188013760
C	5.476542778	-2.625520730	0.809032657
C	6.902197000	-0.142455345	1.624677891
C	4.774220830	0.129735622	2.923240955
N	1.635743812	-2.006209526	-0.833228684
C	0.683761624	-2.937276090	-1.265736587
C	0.449737251	-4.172177769	-0.628574238
C	-0.590183697	-5.000130264	-1.044512914
C	-1.432326544	-4.624045537	-2.095532882
C	-1.218724907	-3.397980745	-2.726518073
C	-0.172835816	-2.569799659	-2.323682957
C	-3.979569857	0.101545021	2.718487933
C	-3.672223096	-1.301152252	3.267822767
C	-4.451877863	1.683801589	0.056620734
C	-4.009407995	2.890330050	0.901575376
C	-2.919212440	1.033793170	3.339932707
C	-5.381484593	0.522766425	3.178060027
C	-3.801654085	1.889062704	-1.325669366
C	-5.979890790	1.694383435	-0.077658001
C	-3.621092477	-2.666178913	0.312987337
C	-5.956370761	-1.754529168	0.558704755
H	3.416115981	3.619474818	-1.964736156
H	6.688001577	2.276906734	0.504671018
H	6.493712722	-2.741992554	1.178199534
H	3.107116288	-4.440581771	-0.880537527
H	7.210879250	-0.955144481	2.294107490
H	7.281547915	0.794278173	2.051720290
H	7.374125662	-0.297388090	0.645916848
H	5.065671348	-0.691786987	3.591492018
H	3.679014896	0.175422070	2.886183519
H	5.144987195	1.075654546	3.340567944
H	5.687653815	3.914059692	-1.054059318
H	5.357980411	-4.651517161	0.095953279
H	-4.286312404	3.805198640	0.356162487
H	-4.497346994	2.931267549	1.880716445
H	-2.919999442	2.905385774	1.041859676
H	-4.115478995	2.870346942	-1.713481914
H	-2.705075546	1.894798843	-1.256120752
H	-4.090655788	1.133205643	-2.060053839
H	-6.291535501	2.670761049	-0.481233444
H	-6.340916257	0.926343137	-0.770965980
H	-6.486301391	1.555127250	0.884249474
H	-2.961965024	0.925597296	4.434656148
H	-1.907946659	0.756310237	3.009043219
H	-3.075413700	2.089030455	3.103834095
H	-3.652571157	-1.238617837	4.366373435
H	-4.430500977	-2.041944728	2.994347145
H	-2.688170553	-1.659106376	2.936218515
H	-5.433474858	0.443767243	4.275460584
H	-5.611692749	1.561411983	2.915841214
H	-6.166257409	-0.118910153	2.761717009
H	-4.017681501	-3.524210470	-0.250743285
H	-2.599585123	-2.479706404	-0.048016994
H	-3.567178326	-2.955608233	1.364978381
H	-6.348148196	-2.626460440	0.011578857
H	-5.973449209	-2.006639148	1.625178246
H	-6.646424271	-0.919783212	0.391488515
H	-4.875726829	-2.240741111	-1.917304053
H	-5.288879671	-0.522203193	-1.804125280
H	-3.577263249	-1.033917358	-1.870944356
H	1.068330528	-4.465148964	0.218786582
H	-0.753403742	-5.948292152	-0.529397488
H	-2.245719881	-5.276497831	-2.413373003
H	-1.865278130	-3.083696919	-3.547131948
H	0.006628957	-1.625717770	-2.841113208
H	0.120186795	0.729130574	-3.212661112
H	-1.613286355	2.153961301	-4.263678485
H	-1.820330903	4.557838247	-3.622607261
H	-0.298693838	5.491142559	-1.879256990
H	1.393384445	4.056123786	-0.794117637
C	1.057175757	2.595702702	2.263974426
C	2.136236633	3.674786741	2.312670765
H	0.233373083	2.812251965	2.959133524
H	2.961233233	3.442463499	1.625798862
H	2.560795105	3.773228302	3.321823486
H	1.462346235	1.609623619	2.531803742
H	1.731949526	4.656617287	2.027237534
H	0.621964331	2.500381678	1.259368243
